# Intersection of Performance, Interpretability, and Fairness in Neural Prototype Tree for Chest X-Ray Pathology Detection: Algorithm Development and Validation Study

**DOI:** 10.2196/59045

**Published:** 2024-12-05

**Authors:** Hongbo Chen, Myrtede Alfred, Andrew D Brown, Angela Atinga, Eldan Cohen

**Affiliations:** 1 Department of Mechanical and Industrial Engineering University of Toronto Toronto, ON Canada; 2 St Michael's Hospital Toronto, ON Canada; 3 Sunnybrook Health Sciences Centre Toronto, ON Canada

**Keywords:** explainable artificial intelligence, deep learning, chest x-ray, thoracic pathology, fairness, interpretability

## Abstract

**Background:**

While deep learning classifiers have shown remarkable results in detecting chest X-ray (CXR) pathologies, their adoption in clinical settings is often hampered by the lack of transparency. To bridge this gap, this study introduces the neural prototype tree (NPT), an interpretable image classifier that combines the diagnostic capability of deep learning models and the interpretability of the decision tree for CXR pathology detection.

**Objective:**

This study aimed to investigate the utility of the NPT classifier in 3 dimensions, including performance, interpretability, and fairness, and subsequently examined the complex interaction between these dimensions. We highlight both local and global explanations of the NPT classifier and discuss its potential utility in clinical settings.

**Methods:**

This study used CXRs from the publicly available Chest X-ray 14, CheXpert, and MIMIC-CXR datasets. We trained 6 separate classifiers for each CXR pathology in all datasets, 1 baseline residual neural network (ResNet)–152, and 5 NPT classifiers with varying levels of interpretability. Performance, interpretability, and fairness were measured using the area under the receiver operating characteristic curve (ROC AUC), interpretation complexity (IC), and mean true positive rate (TPR) disparity, respectively. Linear regression analyses were performed to investigate the relationship between IC and ROC AUC, as well as between IC and mean TPR disparity.

**Results:**

The performance of the NPT classifier improved as the IC level increased, surpassing that of ResNet-152 at IC level 15 for the Chest X-ray 14 dataset and IC level 31 for the CheXpert and MIMIC-CXR datasets. The NPT classifier at IC level 1 exhibited the highest degree of unfairness, as indicated by the mean TPR disparity. The magnitude of unfairness, as measured by the mean TPR disparity, was more pronounced in groups differentiated by age (chest X-ray 14 0.112, SD 0.015; CheXpert 0.097, SD 0.010; MIMIC 0.093, SD 0.017) compared to sex (chest X-ray 14 0.054 SD 0.012; CheXpert 0.062, SD 0.008; MIMIC 0.066, SD 0.013). A significant positive relationship between interpretability (ie, IC level) and performance (ie, ROC AUC) was observed across all CXR pathologies (*P*<.001). Furthermore, linear regression analysis revealed a significant negative relationship between interpretability and fairness (ie, mean TPR disparity) across age and sex subgroups (*P*<.001).

**Conclusions:**

By illuminating the intricate relationship between performance, interpretability, and fairness of the NPT classifier, this research offers insightful perspectives that could guide future developments in effective, interpretable, and equitable deep learning classifiers for CXR pathology detection.

## Introduction

### Challenges in Chest X-Ray Analysis

The chest X-ray (CXR) is a standard imaging procedure for screening, diagnosing, and monitoring a range of critical thoracic conditions, including but not limited to cardiac, pulmonary, and respiratory diseases [1,2]. More than 2 billion CXRs are obtained globally each year, making it one of the most frequently performed radiographic tests [3]. However, interpreting CXRs poses substantial challenges, evidenced by research highlighting substantial interobserver variability among radiologists, leading to inconsistent image analyses [4,5]. In addition, a global shortage of radiologists has been reported. For instance, the United Kingdom only has 8.5 radiologists per 100,000 population, and 96% of the radiology departments reported that they were not able to meet their diagnostic reporting requirements within contracted hours [6,7]. Similarly, in Canada, understaffing of radiologists has not only caused considerable delays in delivering diagnostic results but also led to burnout in up to 72% of radiologists [8,9]. In response to these challenges, substantial research has been invested in developing deep learning classifiers aimed at enhancing the efficiency and accuracy of CXR analysis [4,10-12]. The findings from these studies suggested that deep learning classifiers can reach competitive performance in detecting common CXR pathologies [4,10].

### Adoption of Artificial Intelligence

The adoption of deep learning classifiers for detecting CXR pathologies typically involves a human–artificial intelligence (AI) collaborative approach, wherein the classifier serves as a decision support tool, and the radiologist makes the final judgment [13]. Establishing trust in deep learning–based technologies is a pivotal factor for the successful implementation of human-AI collaboration [14]. Distrust in deep learning–based technologies impedes their adoption and may result in the major loss of opportunities [14,15]. Prior studies found that transparency is a critical element in building trust and promoting the adoption of deep learning–based technologies [14,16]. In the context of CXR pathology detection, transparency means the classifier’s prediction can be explained in a manner that the radiologist can understand and reconstruct the classifier’s reasoning. However, a predominant challenge with most deep learning classifiers is their nontransparent nature, which can obscure the rationale behind their decision-making processes [17,18]. To address this issue, explainable AI (XAI) methods are frequently used to provide explanations of these classifiers’ behavior [4,19,20]. The transparency offered by XAI methods not only helps establish trust in deep learning–based technologies but can also greatly enhance the diagnostic performance of clinicians in medical imaging tasks [21-23].

### The Role of XAI

In the realm of XAI, explanations are generally categorized into 2 main types: post hoc and intrinsic. Post hoc explanations are generated by applying additional XAI tools after the classifier is trained. In contrast, the intrinsic explanations are directly derived from the internal architecture of interpretable classifiers, such as feature weights in the logistic regression [24]. In the context of the CXR pathology classification, class activation maps and integrated gradients, along with their variants, are frequently used to provide post hoc explanations for nontransparent classifiers [23,25]. These tools generate explanations by highlighting the region of the CXR that is most important for the prediction of the classifier. However, previous studies have shown that post hoc explanations can be imprecise due to their reliance on approximations of the classifier’s behavior [11,20,23,26-28]. On the contrary, intrinsic explanations can more precisely explain the classifier’s behavior because these explanations originate directly from the classifier’s internal decision-making process [29]. However, the dilemma arises because conventional interpretable classifiers such as logistic regression and decision trees do not match the predictive performance of more complex, nontransparent classifiers. Balancing the need for accurate explanations with competitive performance remains a critical challenge in CXR pathology detection.

The neural prototype tree (NPT) is one of the most popular interpretable image classifiers, which addresses the performance limitations of the conventional interpretable classifier by combining the expressiveness of the deep learning model with the interpretability of the decision tree [30]. The architecture of the NPT comprises a convolutional neural network followed by a decision tree [30], revealing its decision-making process with a tree-structure explanation. While the NPT presents a promising innovation in integrating interpretability with deep learning capabilities, its practical utility in CXR pathology detection needs to be justified with competitive performance, particularly in comparison with nontransparent deep learning classifiers.

### The Intersection of Performance, Interpretability, and Fairness

Besides interpretability and performance, fairness is another important dimension when considering adopting deep learning–based diagnostic tools for detecting CXR pathologies [31,32]. Deep learning–based diagnostic tools are recognized as potential sources that worsen health inequity through algorithmic bias. In the clinical sense, algorithmic bias can appear as disparities in performance attributed to sex, race, ethnicity, language, socioeconomic status, and other identities that are not indexed to clinical need [33]. For instance, a previous study has highlighted that the state-of-the-art deep learning classifiers for detecting CXR pathologies have a higher false negative rate for Hispanic female patients [34]. The algorithmic bias can lead to unequal access to medical treatment and raises serious ethical concerns. Therefore, it is imperative to comprehensively evaluate the fairness dimension of the NPT classifier to ensure it equitably benefits patients from diverse backgrounds.

The decision tree component of NPT provides transparency in the decision-making process of the classifier. The tree component imposes a constraint on the NPT’s expressivity, which refers to its capacity to model complex patterns and relationships. Increasing the size of the tree can enhance the NPT’s expressivity; however, a larger tree leads to a more complex decision-making process, which reduces the classifier’s interpretability and can impact its performance and fairness. Investigating the relationship between interpretability, performance, and fairness will provide the basis for future studies to better align these 3 dimensions within the NPT classifier for CXR pathology detection.

### Study Objectives

In this study, we systematically trained NPT classifiers with varying sizes of the decision tree component to determine if the interpretable classifier NPT can achieve comparable performance to nontransparent deep learning classifiers for detecting CXR pathologies [12]. Each NPT classifier undergoes a comprehensive evaluation across 3 critical dimensions: performance, interpretability, and fairness. Subsequently, we investigated the intricate relationship among these 3 dimensions. Furthermore, we highlighted both local and global explanations of the NPT classifier and discussed its potential utility in clinical settings.

## Methods

### Data Source

In this study, we used 3 publicly available CXR datasets: Chest X-ray 14 [12], CheXpert [35], and MIMIC-CXR [36]. The Chest X-ray 14 is one of the largest publicly available CXR datasets composed of 112,120 posteroanterior and anteroposterior view CXRs, partitioned into 14 classes. These 15 classes include 14 prevalent CXR pathologies along with no finding class. The dataset was extracted from the clinical Picture Archiving and Communication System database at the National Institutes of Health Clinical Center [12]. CheXpert, on the other hand, contains 224,316 CXRs from 65,401 patients who underwent radiographic examinations at Stanford Health Care between October 2002 and July 2017. The MIMIC-CXR dataset comprises 377,110 CXRs from 65,379 patients evaluated at the Beth Israel Deaconess Medical Center Emergency Department between 2011 and 2016. The labeling of all datasets was performed using natural language processing techniques applied to the corresponding radiology reports. The demographics of the datasets are presented in Table 1.

**Table 1:** Description of chest X-ray (CXR) datasets, Chest X-ray 14, CheXpert, and MIMIC-CXR. The number of CXRs, patients, and the proportion of patients per subgroups of sex, age, and race are presented. The race subgroups include White, Asian, Black, Hispanic, Native American, and others. Age subgroups are categorized into 4 groups: 0 to 25, 26 to 50, 51 to 75, and >75 years. Data for race are only available for CheXpert and MIMIC-CXR.

Attribute			Chest X-ray 14		CheXpert		MIMIC-CXR
CXRs, n			112,120		224,316		377,110
Patients, n			30,805		65,240		65,379
**Sex, n (%)**							
	Female	13,403 (43.51)		29,019 (44.48)		34,252 (52.39)	
	Male	17,402 (56.49)		36,221 (55.52)		31,127 (47.61)	
**Age (y), n (%)**							
	0-25	3891 (12.63)		3197 (4.9)		5230 (8)	
	26-50	12,611 (40.94)		15,514 (23.78)		18,208 (27.85)	
	51-75	13,548 (43.98)		30,656 (46.99)		28,937 (44.26)	
	>75	755 (2.45)		15,873 (24.33)		13,004 (19.89)	
**Race, n (%)**							
	Asian	—^a^		7105 (10.89)		2373 (3.63)	
	Black	—		3164 (4.85)		10,918 (16.7)	
	Hispanic	—		1461 (2.24)		4112 (6.29)	
	Native American	—		1050 (1.61)		157 (0.24)	
	White	—		36,985 (56.69)		42,085 (64.37)	
	Other	—		15,475 (23.72)		5734 (8.77)	

^a^Race data not available for this dataset.

### Ethical Considerations

The datasets used in this study were collected with institutional review board approval from their respective institutions: Chest X-ray 14 was approved by the National Institutes of Health Clinical Center (Bethesda, MD), CheXpert received approval from Stanford Hospital (Stanford, CA), and MIMIC-CXR was approved by the Beth Israel Deaconess Medical Center (Boston, MA) [12,35,36]. As all datasets were fully deidentified [12,35,36], individual patient consent was not required, and this study was exempted from further institutional review board review [37]. To obtain access to these datasets, the authors completed the necessary training courses and signed the corresponding data use agreements, ensuring the appropriate use of the data in accordance with relevant policies and regulations. The lead author completed the CITI Data or Specimens Only Training course (certification number 62353094) to access the MIMIC-CXR dataset. The Chest X-ray 14 and CheXpert datasets did not require specific training.

### NPT Architecture and Inference Logic

**Figure 1:**
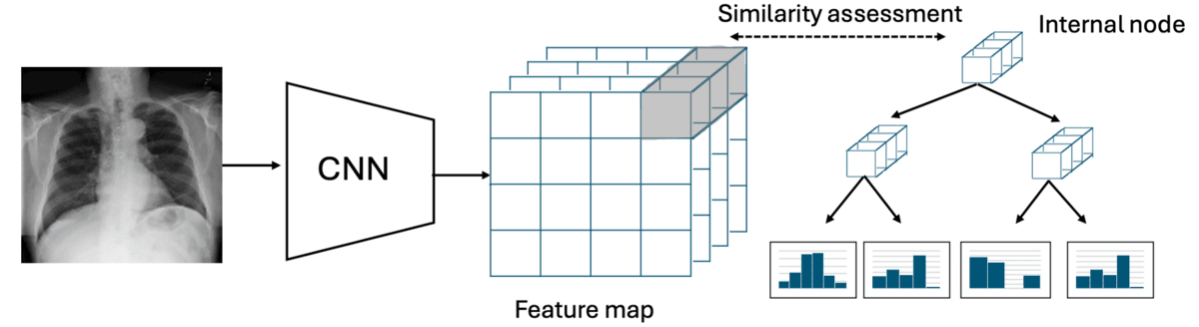
Overview of the neural prototype tree architecture. Chest x-ray images are passed through a pretrained convolutional neural network (CNN), generating feature maps that capture high-level image representations. These feature maps are used as input to a prototype decision tree, where each internal node contains a prototype representing a discriminative patch learned from the training data. The presence of a prototype within an input image’s feature map determines its routing through the decision tree, ultimately arriving at the leaf node to produce the final prediction.

NPT [30] is an interpretable classifier composed of a CNN followed by a prototype decision tree. The architecture and inference logic of the NPT is shown in Figure 1. During training, input images are first passed through a pretrained CNN, such as a residual neural network (ResNet), which extracts a set of latent feature maps. These feature maps capture high-level representations of the image and serve as input to the decision tree component of the NPT. At each internal node of the decision tree, there is a trainable prototype, representing a characteristic part of the training images. These prototypes are initialized as random tensors and refined throughout training. The decision-making at each node is based on the similarity between the most similar image patch in the input feature map of the image and the learned prototype at that node. If the prototype is sufficiently present in the image based on the Euclidean distance, the decision path moves to the right; otherwise, it moves to the left. The training objective is to minimize the cross-entropy loss between the predicted class distribution and the true class label. Both the CNN weights and the prototypes are optimized through backpropagation to ensure accurate classification. By the end of the training, each prototype represents a discriminative patch learned from the training data that is crucial for making classification decisions. The prototypes are then upsampled using bicubic interpolation, enabling visualization in the original image space.

### Classifier Development and Comparison

ResNet [38] is a commonly used CNN architecture for medical imaging tasks [39], which enables training deeper neural networks using residual blocks and skip connections. ResNet has demonstrated exceptional performance in various medical imaging studies [4,40], making it an ideal candidate for comparison with the NPT classifier in CXR pathology detection due to its established accuracy and widespread adoption in the field. We started with training a ResNet-152 classifier for each CXR pathology. The performance of trained classifiers was then compared with recent studies. Upon confirming that the ResNet-152 could reach comparable performance with previous studies, we adopted it as our performance benchmark. This benchmarking laid the groundwork for comparing the interpretable NPT classifiers against nontransparent deep models (ie, ResNet-152). Subsequently, we trained NPT classifiers with different numbers of internal nodes, compared their performance with the benchmark, and investigated whether the NPT classifier could achieve similar performance. We used ResNet-152 as the convolutional backbone of NPT to facilitate this comparison. The ResNet-152 was chosen due to its superior performance in CXR pathology classification compared with other convolutional neural network architectures [41].

Consistent with previous studies [4,42,43], we treated each CXR pathology as an individual binary classification task, and for each CXR pathology, we assigned positive to CXRs with the condition and negative to all others. We combined the nonpositive labels within CheXpert into an aggregate “negative” label similar to previous studies [34]. Anticipating scenarios where a patient’s CXR may exhibit multiple pathologies, we acknowledged the possibility of chaining binary classifiers for multipathology detection using a binary relevance approach [44]; however, applying NPTs to a multilabel classification approach would dramatically increase the tree size due to the numerous prototypes required to achieve optimal performance for detecting all pathologies. This expansion would lead to a large number of internal and leaf nodes, which poses a challenge for interpretability. The dataset for each CXR pathology was randomly split into training (70%), validation (10%), and testing (20%) datasets with no individual patient’s X-rays shared across datasets.

For each CXR pathology, 5 NPT classifiers were independently trained on the anteroposterior and posteroanterior views. The decision was made in consideration of the NPT’s inference process, which relies on the presence of prototype image patches within a CXR. As such, we wanted to ensure that the decision-making process matched the provided explanations derived from a specific view of CXRs. In addition, a single ResNet classifier was trained using a combined dataset of both anteroposterior and posteroanterior views for each pathology. The NPT classifiers varied in the number of internal nodes by adjusting the tree depth. All classifiers were optimized using the AdamW optimizer [42]. The data preprocessing procedures include resizing the input CXR to a dimension of 224×224×3 and normalization based on the mean and SD of images in the ImageNet dataset [45]. Additionally, we used data augmentation techniques, including random horizontal flip, random affine, and random crop [46-48]. Hyperparameters, including learning rate, batch size, and degree of random affine, were selected based on the model’s area under the receiver operating characteristic curve (ROC AUC) score on the validation dataset. The number of epochs was determined by using an early stopping condition, where training was terminated if the validation loss did not decrease for 10 epochs. To ensure robustness, each classifier was trained 5 times, with the dataset being randomly reshuffled each time. The classifiers’ performance measures were reported with their means and 95% CIs based on these 5 runs.

### Performance, Interpretability, and Fairness Measurement

The performance of trained classifiers was evaluated using ROC AUC. ROC AUC is a widely used metric for evaluating a classifier’s performance. It is computed by plotting the true positive rate (TPR) against the false positive rate and calculating the area under the curve. The metric provides a comprehensive evaluation of the classifier’s overall performance, capturing its proficiency in distinguishing positive and negative classes across various classification thresholds.

The interpretability of classifiers was quantified by the interpretation complexity (IC), which refers to the count of decision thresholds present in a model [49]. For tree-based classifiers, IC corresponds to the number of internal nodes, each internal node assesses the presence of a prototype image patch [49]. A lower IC value indicates a more interpretable classifier [50,51]. Intuitively, increasing the number of nodes leads to more decision-making steps that involve determining the presence of more prototype image patches in a CXR. This will increase the complexity of the model and decrease the ability to comprehend the model’s rationale for making a prediction. In this study, we adjusted the tree depth of the NPT classifier to control the number of nodes, thus achieving various levels of interpretability as delineated by IC. To be specific, we trained NPT classifiers with tree depths ranging from 1 to 5, corresponding to an IC of 1, 3, 7, 15, and 31.

To evaluate the classifiers’ fairness dimension, we used equality of opportunity as our fairness criterion [52]. According to this criterion, a classifier is considered fair if the TPR (ie, recall) is the same across subgroups defined by distinct protected attributes (ie, sex, age, and race). We focused on evaluating the fairness of the NPT classifier with respect to patient demographics, including age and sex for the Chest X-ray 14 dataset and age, sex, and race for the CheXpert dataset. The sex categories include male and female, while the age groups are divided into 4 intervals: 0 to 25, 26 to 50, 51 to 75, and >75 years. The race groups for the CheXpert dataset include White, Asian, Native American, Hispanic, and Black. The bias on the subgroup level is quantified with the TPR disparity [34,53,54]. For groups differentiated by sex, the TPR disparity is computed as the difference between the model’s TPR on the group’s CXRs and the complementary group’s CXRs [54]. When considering subgroups differentiated by age and race, the TPR disparity is computed as the difference between the TPR of a specific age group and the median TPR across all subgroups [34]. The TPR disparity values can range from −1 to 1, with negative values indicating the classifier is biased against a particular subgroup. Subsequently, we identified favorable and unfavorable subgroups based on the frequency of positive or negative TPR disparity across all pathologies. For instance, if male patients had positive TPR disparity in 10 out of 14 pathologies, it would be categorized as a favored group. We quantified the NPT classifier’s degree of fairness using the mean TPR disparity [34]. For NPT classifiers with different ICs, the mean TPR disparity was computed by first determining the largest TPR disparity at the subgroup level (ie, sex, age, and race) and subsequently averaging these values across all CXR pathologies. A higher mean TPR disparity indicates a greater potential for unfair diagnosis of certain subgroups by the classifier.

### The Intersection Between Performance, Interpretability, and Fairness

A tree with a greater IC (ie, a larger number of internal nodes) can express more complex relationships, potentially leading to improved performance. Nonetheless, as the IC increases, the decision-making process becomes more complicated, potentially diminishing the classifier’s interpretability. To investigate the relationship between interpretability and performance, we conducted simple linear regression to determine the ability of NPT’s IC to predict its ROC AUC in detecting 5 CXR pathologies.

Previous studies have suggested that simplifying the model (ie, reducing the number of internal nodes) to enhance interpretability may adversely impact the model’s fairness [55,56]. To further understand the relationship between interpretability and fairness, we performed simple linear regression to assess the ability of IC to predict the mean TPR disparity across sex, age, and race-differentiated subgroups. The linear regression model parameters were estimated using the ordinary least squares method. Each linear regression analysis was performed using 25 data points from 5 separate runs for each of the 5 NPT classifiers with different ICs. We used RStudio (version 4.2.1; The R Foundation) for conducting linear regressions. To control the false discovery rate, we applied the Benjamini-Hochberg correction to adjust the *P* values obtained from statistical analyses.

### Local and Global Explanation Generation

The NPT classifier [30] can provide both local and global explanations. The global explanation is directly derived from the decision tree architecture. The explanation exposes the potential paths that an input CXR might undertake, providing a holistic perspective into the classifier’s decision-making mechanism for detecting pathologies. The process for generating local explanations commences with navigating the input image through the decision tree, wherein the presence of prototype image patches within the image influences its path through the decision tree until it reaches a leaf node. These leaf nodes carry probability distributions over classes and lead to the final prediction. In this study, we first presented an example of a global explanation for the NPT classifier trained to detect atelectasis. Then, we presented an example of a local explanation for an atelectasis CXR. We subsequently discussed the potential utility and implications of these explanations.

## Results

### Performance Comparison Between NPT and ResNet-152

**Figure 2:**
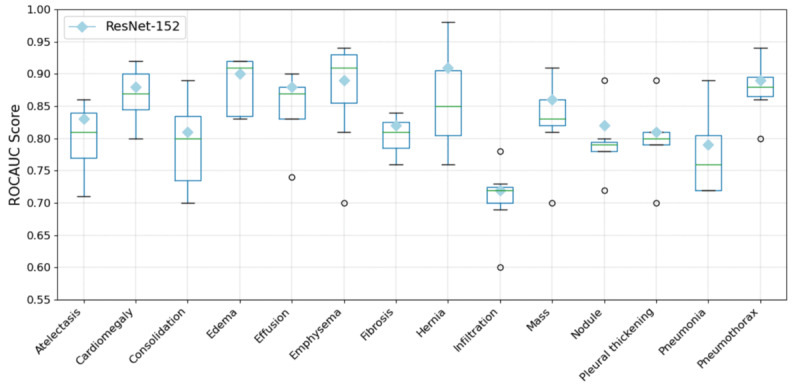
Box plot comparing the area under the receiver operating characteristic curve (ROC AUC) performance of the residual neural network (ResNet)–152 classifier with recent studies on the Chest X-ray 14 dataset. The diamond symbol represents the ROC AUC of the ResNet-152 classifier. The plot visually demonstrates how the performance of ResNet-152 aligns closely with the median performance of recent studies across 14 pathologies.

Before evaluating the performance of the NPT classifier against ResNet-152, we first benchmarked the trained ResNet-152 model against established studies to ensure it reached a competitive performance level. A detailed comparison of the results is provided in Multimedia Appendix 1. The box plot in Figure 2 provides a visual comparison between the performance of the ResNet-152 classifier and results from recent studies. The diamond markers represent the ROC AUC scores of the ResNet-152 classifier across various pathologies. ResNet-152 exhibits competitive performance, surpassing the median performance of recent studies in 12 out of the 14 pathologies within the Chest X-ray 14 dataset.

**Figure 3:**
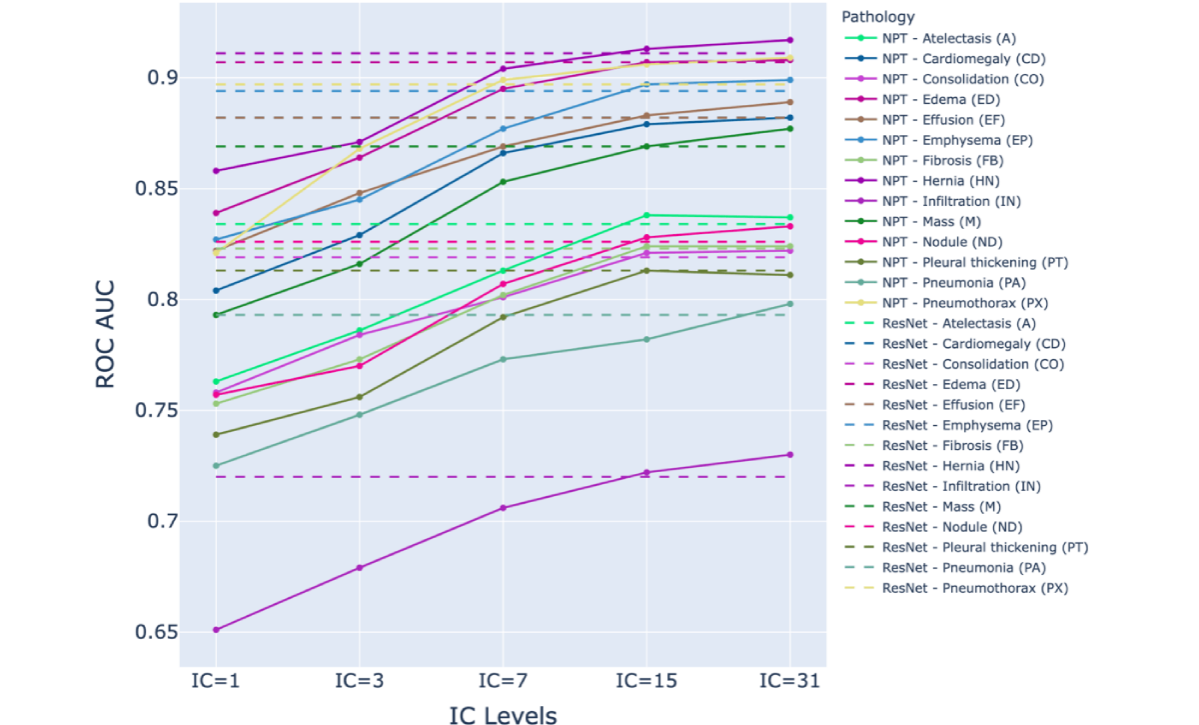
Comparison of area under the receiver operating characteristic curve (ROC AUC) performance between residual neural network (ResNet)–152 (dashed lines) and neural prototype tree (NPT) classifiers (solid lines) across varying IC levels for different pathologies in the Chest X-ray 14 dataset. As the interpretation complexity (IC) level increases, the NPT performance generally improves, with several pathologies surpassing ResNet-152's performance at IC levels 15 and 31. The dashed lines represent ResNet-152 performance, while colored solid lines represent NPT performance for each pathology.

**Figure 4:**
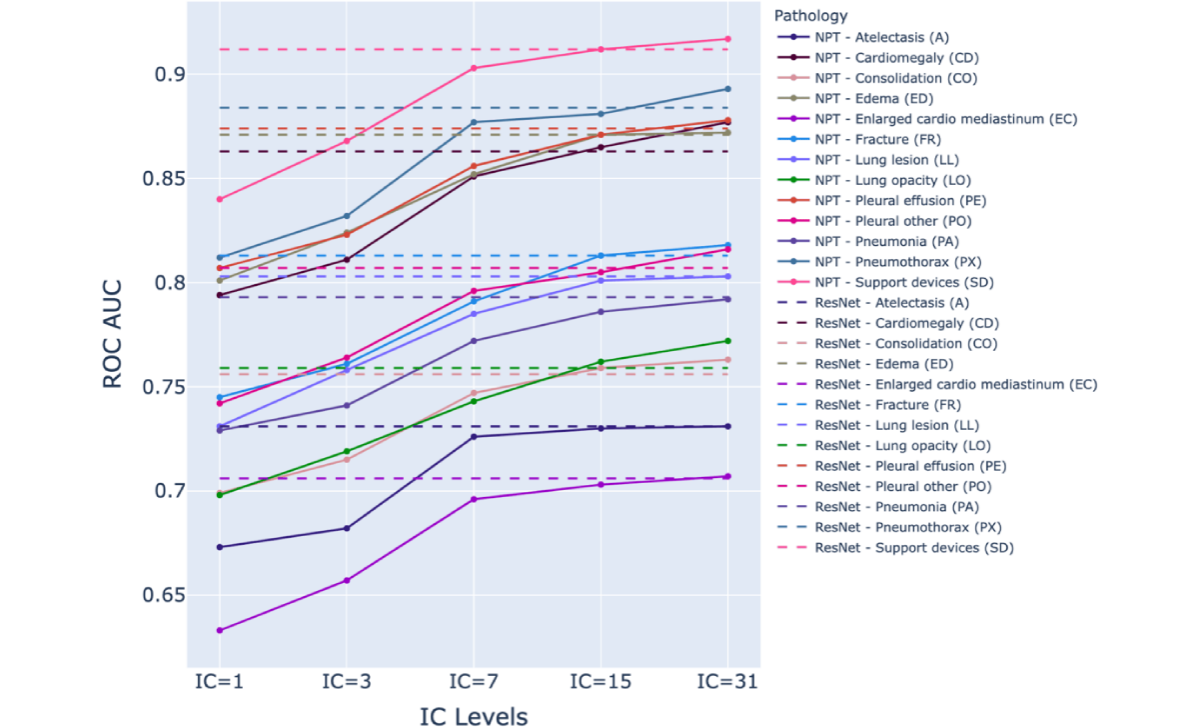
Comparison of area under the receiver operating characteristic curve (ROC AUC) performance between residual neural network (ResNet)–152 (dashed lines) and neural prototype tree (NPT) classifiers (solid lines) across varying interpretation complexity (IC) levels for different pathologies in the CheXpert dataset. As the IC level increases, the NPT performance generally improves, with several pathologies surpassing ResNet-152’s performance at IC levels 15 and 31. The dashed lines represent ResNet-152 performance, while colored solid lines represent NPT performance for each pathology.

**Figure 5:**
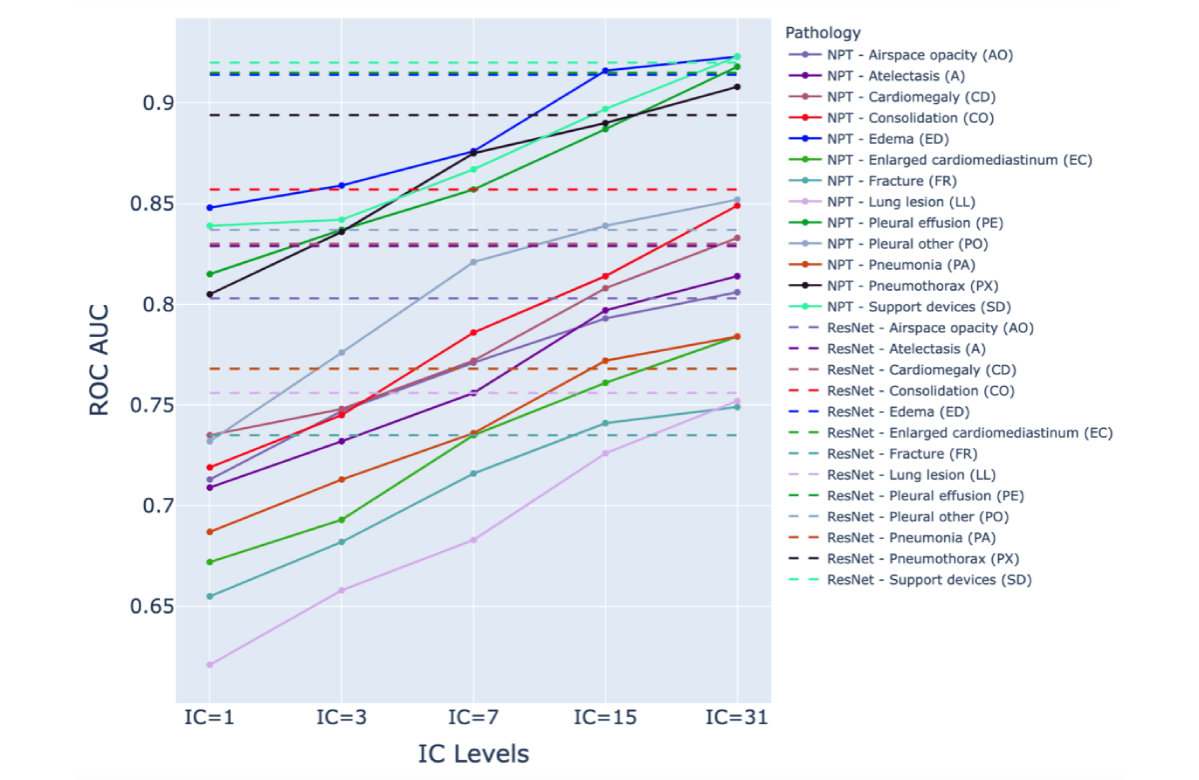
Comparison of area under the receiver operating characteristic curve (ROC AUC) performance between residual neural network (ResNet)–152 (dashed lines) and neural prototype tree (NPT) classifiers (solid lines) across varying interpretation complexity (IC) levels for different pathologies in the MIMIC-chest x-ray (CXR) dataset. As the IC level increases, the NPT performance generally improves, with several pathologies surpassing ResNet-152’s performance at IC levels 15 and 31. The dashed lines represent ResNet-152 performance, while colored solid lines represent NPT performance for each pathology.

**Figure 6:**
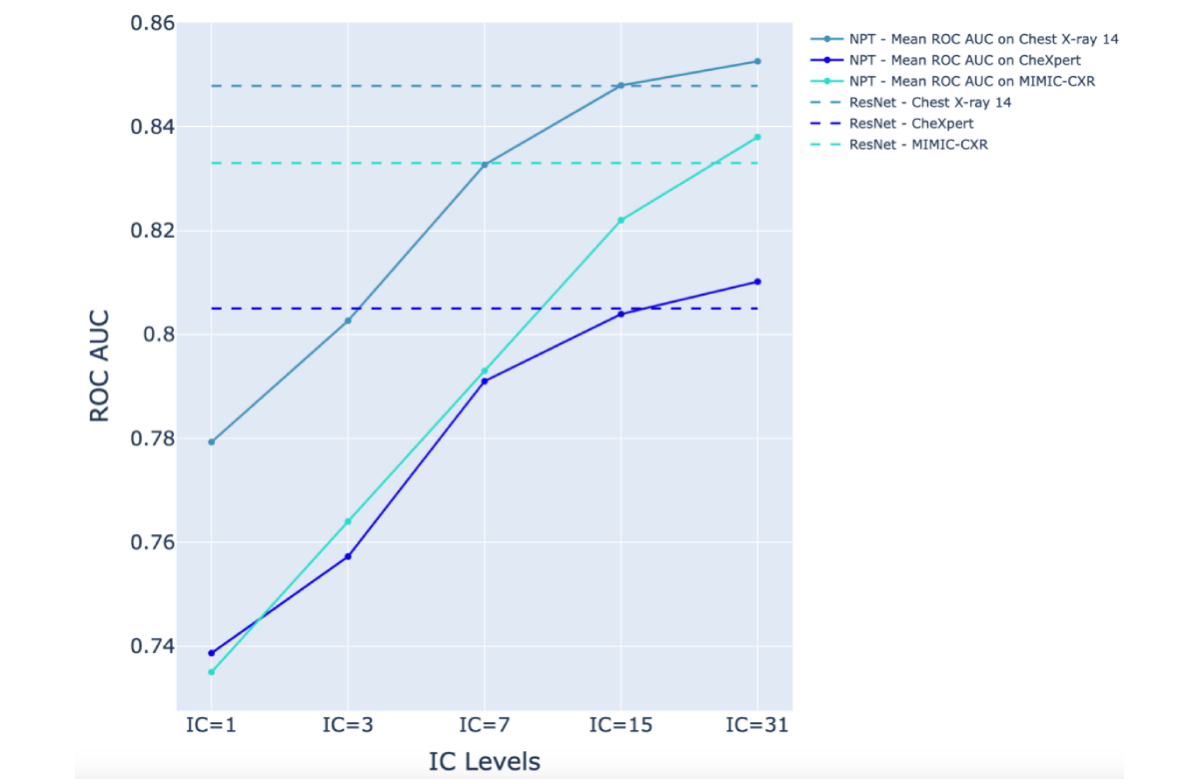
Comparison of mean area under the receiver operating characteristic curve (ROC AUC) performance between residual neural network (ResNet)–152 (dashed lines) and neural prototype tree (NPT) classifiers (solid lines) across varying IC levels for the Chest X-ray 14, CheXpert, and MIMIC-CXR datasets. As the interpretation complexity (IC) level increases, NPT performance improves, surpassing ResNet-152 at IC level 15 for the Chest X-ray 14 dataset and IC level 31 for the CheXpert and MIMIC-chest x-ray (CXR) datasets.

The ROC AUC performance of the ResNet-152 and NPT classifiers across various IC levels in detecting pathologies is presented in Multimedia Appendix 2. Figures 3-5 illustrate the NPT performance as a function of IC level for the Chest X-ray 14, CheXpert, and MIMIC-CXR datasets, respectively. The results show that the performance of the NPT classifiers generally improved with increasing IC levels, eventually surpassing the performance of ResNet-152 at IC levels 15 or 31 for most pathologies. This pattern was consistently observed across 3 datasets. The mean ROC AUC of ResNet-152 and NPT classifiers across all pathologies in Chest X-ray 14, CheXpert, and MIMIC-CXR are presented in Table 2. Figure 6 illustrates the mean ROC AUC values of ResNet-152 and NPT classifiers across different IC levels for all pathologies in 3 datasets. In the Chest X-ray 14 dataset, the NPT classifier outperformed ResNet-152 at IC level 15, while in the CheXpert and MIMIC-CXR datasets, this outperformance occurred at IC level 31.

**Table 2:** The mean area under the receiver operating characteristic curve (ROC AUC) performance of residual neural network (ResNet)–152 and neural prototype tree (NPT) classifiers across varying interpretation complexity (IC) levels for all pathologies in the Chest X-ray 14, CheXpert, and MIMIC-CXR datasets. As the IC level increases, NPT performance improves, surpassing ResNet-152 at IC level 15 for the Chest X-ray 14 dataset and IC level 31 for the CheXpert and MIMIC-CXR datasets.

Dataset	ResNet-152, mean (SD)	NPT (IC=1), mean (SD)	NPT (IC=3), mean (SD)	NPT (IC=7), mean (SD)	NPT (IC=15), mean (SD)	NPT (IC=31), mean (SD)
Chest X-Ray 14	0.847 (0.054)	0.779 (0.036)	0.803 (0.054)	0.833 (0.049)	0.848 (0.061)	0.853 (0.057)
CheXpert	0.805 (0.062)	0.739 (0.042)	0.757 (0.039)	0.791 (0.053)	0.804 (0.048)	0.810 (0.063)
MIMIC-CXR	0.833 (0.065)	0.735 (0.052)	0.759 (0.071)	0.789 (0.056)	0.819 (0.054)	0.838 (0.061)

We performed 14 linear regression analyses on the Chest X-ray 14 dataset and 13 linear regression analyses on both the CheXpert and MIMIC-CXR datasets, with each analysis corresponding to a distinct pathology. These analyses aimed to assess the influence of IC levels on the ROC AUC of NPT classifiers. To account for the false discovery rate, we applied the Benjamini-Hochberg correction to adjust the *P* values. The detailed results of these analyses are provided in Multimedia Appendix 3. A statistically significant positive association (*P*<.001) was observed between IC levels and ROC AUC across all pathologies in each dataset. The results highlight a tradeoff between model performance and interpretability, wherein an increase in IC levels improved performance but simultaneously led to a reduction in interpretability.

### Fairness Assessment of NPT Classifiers

The TPR disparity of NPT classifiers across various demographic attributes for classifying CXR pathologies is presented in Multimedia Appendix 4. A summary of the fairness assessment of NPT classifiers at different IC levels is provided in Table 3.

**Figure 7:**
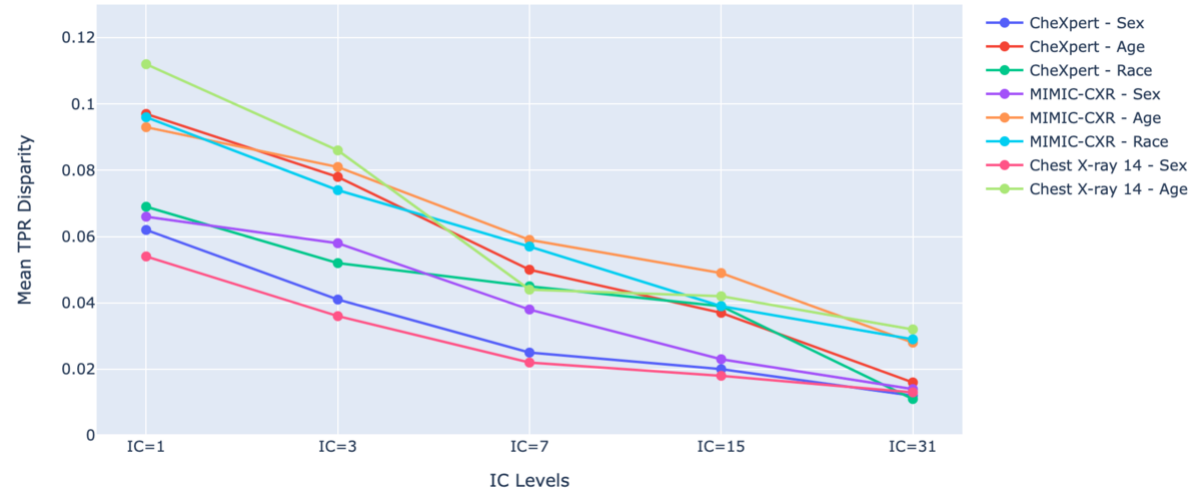
Mean true positive rate (TPR) disparity across varying interpretation complexity (IC) levels for different demographic attributes (sex, age, and race) in the CheXpert, MIMIC-chest x-ray (CXR), and Chest X-ray 14 datasets. As IC levels increase, the mean TPR disparity decreases consistently across all demographic groups, indicating improved fairness in classification at higher IC levels.

**Figure 8:**
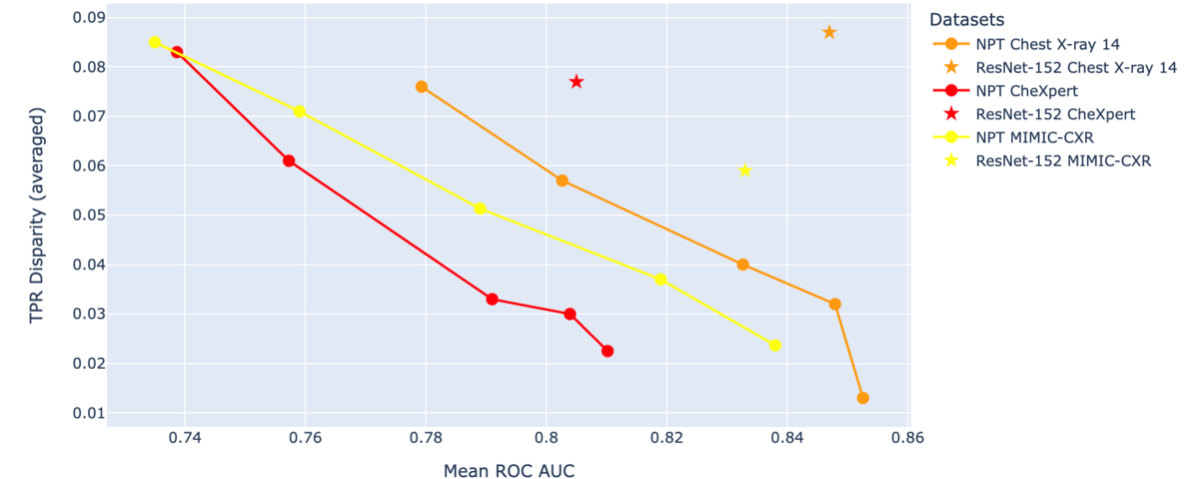
The relationship between true positive rate (TPR) disparity (averaged across demographic attributes) and mean area under the receiver operating characteristic curve (ROC AUC) across all pathologies for neural prototype tree (NPT) and residual neural network (ResNet)–152 classifiers, evaluated on the Chest X-ray 14, CheXpert, and MIMIC-chest x-ray (CXR) datasets. The figure revealed an inverse relationship between the mean TPR disparity and the mean ROC AUC for NPT classifiers, indicating improved fairness with higher performance of NPT classifiers.

Male patients are frequently classified as favorable (ie, exhibiting more positive TPR disparities across pathologies compared with other groups), particularly at lower IC levels (IC=1 and IC=3). The 26-50- and 51-75-year age groups were found to be favorable across different IC levels, while the 0-25-year age group was consistently found as unfavorable. Regarding racial groups, White individuals were found to be favorable across all IC levels, whereas Black and Hispanic individuals tended to exhibit negative TPR disparities and were frequently found to be unfavorable across different IC levels. The highest mean TPR disparities were consistently observed at IC level 1 across all datasets. Figure 7 presents the mean TPR disparity across various IC levels for different demographic attributes (sex, age, and race) in the CheXpert, MIMIC-CXR, and Chest X-ray 14 datasets. The results showed a consistent decrease in mean TPR disparity across all groups and datasets as IC levels increased, indicating a reduction in disparity with higher IC levels. To investigate the tradeoff between fairness and performance, we calculated the average TPR disparity across demographic attributes for Chest X-ray 14, MIMIC-CXR, and CheXpert datasets. These values were then plotted against the mean ROC AUC in Figure 8. Figure 8 revealed an inverse relationship between the TPR disparity and the mean ROC AUC. ResNet-152 classifiers consistently exhibited higher TPR disparity compared with NPT classifiers when achieving similar mean ROC AUC across 3 datasets.

We conducted linear regression analyses to examine the effect of IC levels on mean TPR disparity across demographic attributes, specifically sex and age in the Chest X-ray 14 dataset and sex, age, and race in the CheXpert, MIMIC-CXR datasets. A detailed statistical analysis is provided in Multimedia Appendix 5. The results indicated a statistically significant negative relationship (*P*<.001) between IC levels and mean TPR disparity for all demographic attributes in all datasets.

**Table 3:** Overview of true positive rate (TPR) disparities across different demographic attributes (sex, age, and race) in the CheXpert, MIMIC-CXR, and Chest X-ray 14 datasets. The table identifies the “favorable” and “unfavorable” subgroups, defined as those with more positive or negative TPR disparities, respectively, when compared to other groups across pathologies. The mean TPR disparity is calculated by averaging the largest disparities associated with each attribute (sex, age, and race) across all pathologies. Both datasets list the most frequent favorable and unfavorable subgroups for each interpretation complexity (IC) level, illustrating the disparity patterns across demographics.

IC level and attribute				Sex		Age (y)		Race
**Chest X-ray 14**								
	**IC=1**							
		Favorable	Male		26-50		—^a^	
		Pathologies with higher TPR, n (%)	14 (100)		14 (100)		—	
		Unfavorable	Female		0-25		—	
		Pathologies with lower TPR, n (%)	14 (100)		14 (100)		—	
		TPR disparity, mean (SD)	0.054 (0.012)		0.112 (0.015)		—	
	**IC=3**							
		Favorable	Male		51-75, >75		—	
		Pathologies with higher TPR, n (%)	14 (100)		10 (71)		—	
		Unfavorable	Female		0-25		—	
		Pathologies with lower TPR, n (%)	14 (100)		14 (100)		—	
		TPR disparity, mean (SD)	0.036 (0.009)		0.086 (0.013)		—	
	**IC=7**							
		Favorable	Male		26-50		—	
		Pathologies with higher TPR, n (%)	8 (57)		11 (79)		—	
		Unfavorable	Female		0-25		—	
		Pathologies with lower TPR, n (%)	8 (57)		14 (100)		—	
		TPR disparity, mean (SD)	0.022 (0.007)		0.044 (0.009)		—	
	**IC=15**							
		Favorable	Male		51-75		—	
		Pathologies with higher TPR, n (%)	11 (79)		10 (71)		—	
		Unfavorable	Female		0-25		—	
		Pathologies with lower TPR, n (%)	11 (79)		12 (86)		—	
		TPR disparity, mean (SD)	0.018 (0.004)		0.042 (0.011)		—	
	**IC=31**							
		Favorable	Female		>75		—	
		Pathologies with higher TPR, n (%)	8 (57)		11 (79)		—	
		Unfavorable	Male		0-25		—	
		Pathologies with lower TPR, n (%)	8 (57)		14 (100)		—	
		TPR disparity, mean (SD)	0.013 (0.003)		0.032 (0.005)		—	
**CheXpert**								
	**IC=1**							
		Favorable	Male		26-50		White	
		Pathologies with higher TPR, n (%)	13 (100)		13 (100)		12 (92)	
		Unfavorable	Female		0-25		Hispanic	
		Pathologies with lower TPR, n (%)	13 (100)		13 (100)		11 (85)	
		TPR disparity, mean (SD)	0.062 (0.008)		0.097 (0.010)		0.069 (0.013)	
	**IC=3**							
		Favorable	Male		51-75		White	
		Pathologies with higher TPR, n (%)	13 (100)		11 (85)		12 (92)	
		Unfavorable	Female		0-25		Black	
		Pathologies with lower TPR, n (%)	13 (100)		13 (100)		13 (100)	
		TPR disparity, mean (SD)	0.041 (0.009)		0.078 (0.013)		0.052 (0.011)	
	**IC=7**							
		Favorable	Female		51-75		White	
		Pathologies with higher TPR, n (%)	7 (54)		9 (69)		13 (100)	
		Unfavorable	Male		0-25		Hispanic	
		Pathologies with lower TPR, n (%)	7 (54)		13 (100)		9 (69)	
		TPR disparity, mean (SD)	0.025 (0.005)		0.050 (0.014)		0.045 (0.008)	
	**IC=15**							
		Favorable	Male		51-75		White	
		Pathologies with higher TPR, n (%)	12 (92)		11 (85)		9 (69)	
		Unfavorable	Female		0-25		Black	
		Pathologies with lower TPR, n (%)	12 (92)		10 (77)		11 (85)	
		TPR disparity, mean (SD)	0.020 (0.004)		0.037 (0.008)		0.039 (0.006)	
	**IC=31**							
		Favorable	Male		51-75		White	
		Pathologies with higher TPR, n (%)	13 (100)		12 (92)		10 (77)	
		Unfavorable	Female		0-25		Black	
		Pathologies with lower TPR, n (%)	13 (100)		13 (100)		10 (77)	
		TPR disparity, mean (SD)	0.012 (0.002)		0.016 (0.004)		0.011 (0.003)	
**MIMIC-CXR**								
	**IC=1**							
		Favorable	Male		26-50		White	
		Pathologies with higher TPR, n (%)	13 (100)		13 (100)		12 (92)	
		Unfavorable	Female		0-25		Hispanic, Asian, Black	
		Pathologies with lower TPR, n (%)	13 (100)		13 (100)		10 (77)	
		TPR disparity, mean (SD)	0.066 (0.013)		0.093 (0.017)		0.096 (0.021)	
	**IC=3**							
		Favorable	Male		51-75		White	
		Pathologies with higher TPR, n (%)	13 (100)		10 (77)		11 (85)	
		Unfavorable	Female		0-25		Black	
		Pathologies with lower TPR, n (%)	13 (100)		12 (92)		11 (85)	
		TPR disparity, mean (SD)	0.058 (0.012)		0.081 (0.015)		0.074 (0.016_	
	**IC=7**							
		Favorable	Male		26-50, 51-75		White	
		Pathologies with higher TPR, n (%)	8 (62)		8 (62)		13 (100)	
		Unfavorable	Female		0-25		Black	
		Pathologies with lower TPR, n (%)	8 (62)		11 (85)		12 (92)	
		TPR disparity, mean (SD)	0.038 (0.009)		0.059 (0.013)		0.057 (0.014)	
	**IC=15**							
		Favorable	Male		51-75, >75		White	
		Pathologies with higher TPR, n (%)	7 (54)		8 (62)		11 (85)	
		Unfavorable	Female		0-25		Black	
		Pathologies with lower TPR, n (%)	7 (54)		11 (85)		10 (77)	
		TPR disparity, mean (SD)	0.023 (0.006)		0.049 (0.009)		0.039 (0.005)	
	**IC=31**							
		Favorable	Male		51-75		White	
		Pathologies with higher TPR, n (%)	9 (69)		9 (69)		9 (69)	
		Unfavorable	Female		0-25		Native American	
		Pathologies with lower TPR, n (%)	9 (69)		8 (62)		11 (85)	
		TPR disparity, mean (SD)	0.014 (0.002)		0.028 (0.008)		0.029 (0.007)	

^a^Race data not available for this dataset.

### Local and Global Explanations of the NPT Classifier

**Figure 9:**
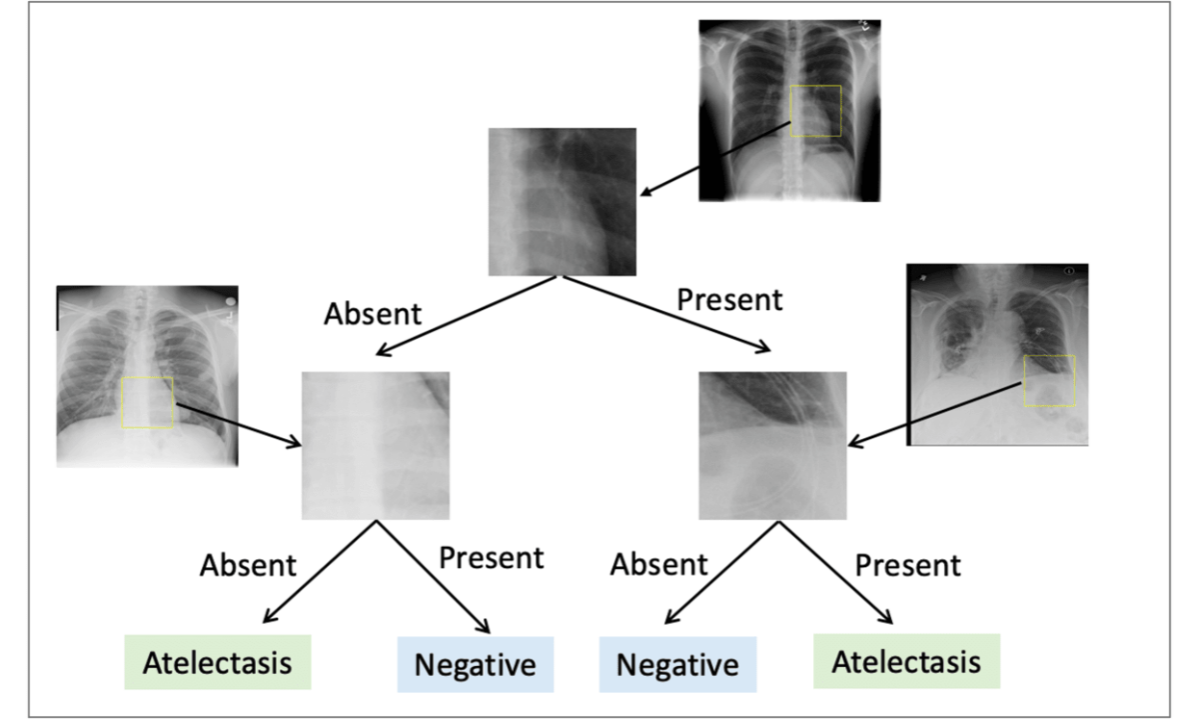
Global explanation of the neural prototype tree (NPT) classifier’s decision-making process for detecting atelectasis (interpretation complexity=3). The diagram illustrates the NPT classifier’s decision-making logic, starting from the root node, where it assesses the presence or absence of discriminative prototypes in the chest x-ray associated with atelectasis. This evaluation progresses through internal nodes, ultimately leading to the final classification at the leaf nodes.

**Figure 10:**
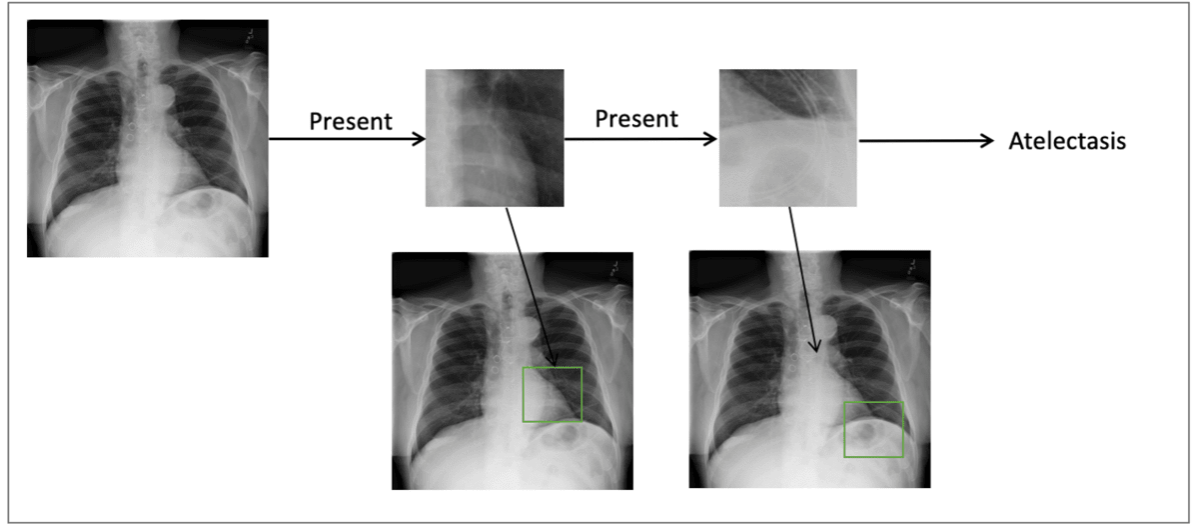
Local explanation of the neural prototype tree (NPT) classifier’s decision-making process for detecting atelectasis in a chest x-ray (CXR). The NPT classifier starts by identifying the most relevant region in the input CXR and comparing it to internal prototype images that capture key features of atelectasis. This culminates in a final positive classification for atelectasis.

Figure 9 presents an example of a global explanation of the NPT classifier (IC=3) for detecting atelectasis. The pathways from the root node to the leaf node reveal the NPT classifier’s decision-making mechanism in detecting atelectasis. At each internal node, the NPT classifier identified the presence of specific signs linked to atelectasis within the input CXR. It then decided on the subsequent pathway, ultimately leading to the final classification at a leaf node. Figure 10 presents an example of a local explanation of the NPT classifier for a sample CXR indicating atelectasis. The NPT classifier started with locating the nearest matching patch in the input CXR to the internal node’s prototype image patch. Following this, the detection of relevant signs of atelectasis within the CXR guided the CXR to the rightmost leaf node, resulting in a positive prediction for atelectasis. We present more examples of NPT classifiers’ global explanation for detecting CXR pathologies in Multimedia Appendix 6.

## Discussion

### Principal Findings

Deep learning classifiers have attracted substantial interest due to their exceptional performance in detecting CXR pathologies [4,57]. However, incorporating such systems within clinical contexts requires diligent evaluation due to their direct impact on patient care. Regulatory frameworks for AI have been proposed worldwide, such as the Artificial Intelligence Act in Europe [58] and the New Generation Artificial Intelligence Ethics Specification in China [59]. These regulations underscore the paramount importance of interpretability and fairness. In the context of CXR pathology detection, post hoc XAI tools are frequently used to enhance interpretability for the nontransparent deep learning classifiers. However, these tools may not accurately describe the classifier’s behavior and provide unfaithful explanations [60]. In response to this issue, we used an interpretable classifier, NPT [30], for CXR pathology detection and subsequently assessed its utility in 3 dimensions, including performance, interpretability, and fairness. To the best of our knowledge, this is the first time that NPT has been used for detecting CXR pathologies. Furthermore, we investigated the intricate relationship between the NPT classifier’s performance, interpretability, and fairness in the context of CXR pathology detection.

In this study, we have shown that the NPT classifier can achieve competitive performance similar to the baseline classifier (ie, ResNet-152) and recent studies for classifying CXR pathologies in Chest X-ray 14 dataset [4,12,20,61-63], suggesting the potential of using the NPT classifier instead of nontransparent deep learning classifiers. The linear regression analysis revealed that the IC was positively correlated with ROC AUC for all CXR pathologies in this study (*P*<.001), indicating a tradeoff between interpretability and performance. As the IC increases, the decision-making process is more complex and leads to decreased interpretability, which may present challenges for clinicians in understanding and tracing the model’s reasoning. This tradeoff highlights the necessity to find a balance between the performance and interpretability of NPT. Therefore, choosing an appropriate IC level (ie, the number of internal nodes) is essential to maintain this balance, ensuring that the NPT classifier is not only effective in detecting CXR pathologies but also remains interpretable for clinical use.

Furthermore, we have observed biases in NPT classifiers across subgroups differentiated by sex, age, and race. The NPT classifiers with an IC level of 1 exhibited the highest level of unfairness indicated by mean TPR disparity for all demographic attributes in the Chest X-ray 14, CheXpert, and MIMIC-CXR datasets. The magnitude of unfairness, quantified with mean TPR disparity, was found to be more pronounced in groups differentiated by age (Chest X-ray 14 0.112, SD 0.015; CheXpert 0.097, SD 0.010; MIMIC-CXR 0.093, SD 0.017) rather than sex (Chest X-ray 14 0.054, SD 0.012; CheXpert 0.062, SD 0.008; MIMIC-CXR 0.066, SD 0.013). This observation aligns with the study by Seyyed-Kalantari et al [34] on identifying fairness gaps in state-of-the-art deep learning classifiers for CXR pathology detection. The linear regression analysis demonstrated a significant negative relationship between IC and mean TPR disparity for both age and sex-differentiated subgroups (*P*<.001) in Chest X-ray 14 and age, sex, and race-differentiated subgroups (*P*<.001) in the CheXpert and MIMIC-CXR datasets, highlighting the tension between interpretability and fairness. This conflict can be attributed to the fact that the tree with a low IC level has a limited capacity to capture the nuances useful for smaller subgroups within the dataset [55]. It is imperative for future studies to devise strategies that reconcile the tension between interpretability and fairness within NPT classifiers, ensuring that the drive for interpretability does not inadvertently perpetuate or amplify demographic biases for detecting CXR pathologies. Various bias reduction techniques have been proposed, such as diversifying training datasets [31,64], detecting and mitigating shortcut learning [65,66], and applying fairness-aware machine-learning techniques [67]; however, the application of these techniques in the context of interpretable CXR classifiers remain unexplored and warrant further investigation.

The global explanation derived from the NPT classifier offers a transparent and traceable decision-making process. This enables radiologists to assess whether the NPT classifier is effectively using relevant signs for detecting certain CXR pathology. Understanding the classifier’s behavior before deployment can help establish trust, facilitate adoption, and mitigate the risk of exposing patients to a poorly trained classifier. The proposed approach addresses a critical limitation of post hoc XAI tools, which is the lack of a convenient and reliable method for assessing the overall quality of generated explanations [26,68,69]. The local explanation provided by the NPT classifier consists of a series of questions about the presence of different signs for certain CXR pathology in the input CXR. In the event of misclassification, the responsible prototype image patch can be easily traced to facilitate error analysis and enable users to pinpoint the factors contributing to misclassifications, aiding in the refinement and improvement of the NPT classifier’s performance. The explanations for deep learning classifiers are most effective when they resonate with the mental model of the radiologists [70]. By providing explanations that mirror the hierarchical reasoning used in CXR diagnosis, such as differential diagnosis pathways, explanations can become intuitive extensions of the reasoning by radiologists [71]. This congruence can lead to a higher degree of trust and a smoother integration into clinical practice. It should also be noted that the explanations provided herein are intended for illustrative purposes only and have not undergone thorough clinical evaluation by radiologists for diagnostic use. As part of ongoing research, future work will focus on conducting comprehensive clinical evaluations to assess the diagnostic utility and validity of these explanations.

### Comparison With Prior Work

Numerous studies have investigated deep learning classifiers for CXR pathology detection [4,12,20,42,72], but their lack of transparency often limits their applicability in clinical settings [16,73,74]. Previous work has used post hoc XAI techniques for explaining the prediction of CXR pathology classifiers [4,75]; however, post hoc XAI techniques only approximate the behavior of the model and, therefore, may not provide faithful explanations [60,76]. Research into the use of interpretable classifiers for CXR pathology detection remains limited. Sun et al [77] proposed a novel interpretable image classifier, which can provide local counterfactual explanations. However, their approach cannot generate global explanations for the classifier’s decision-making mechanism. Yan et al [78] proposed a vision-language model, offering concept-based explanations. While innovative, their approach demands substantial effort from radiologists for concept creation and verification and does not provide global explanations. In contrast, this study used the interpretable model NPT, which could provide both local and global explanations [30]. Furthermore, the relationship between interpretability and fairness has not been well understood, nor has it been investigated in the context of CXR pathology detection. Doshi-Velez and Kim [51] suggested that enhanced model interpretability facilitates analysis, aiding in assessing the fairness dimension. Conversely, Kleinberg and Mullainathan [55] and Agarwal [56] found that simpler, more interpretable models might intensify biases against disadvantaged groups. We conducted a thorough investigation of the relationship between performance, interpretability, and fairness in CXR pathology detection using the interpretable NPT model. This study has shown that interpretability is negatively correlated with performance and fairness. The empirical evidence sheds light on the intricate balances and connections among these critical dimensions in a comprehensive manner.

### Limitations

This study has several limitations that should be acknowledged. First, the datasets used in this study were automatically labeled using natural language processing techniques, which might lead to some mistakes in the labels [12]. Future research should consider addressing this limitation by using additional validation methods or incorporating expert reviews to validate the accuracy and quality of the CXR labels. Second, this study did not incorporate the PadChest dataset due to its substantial size, which exceeded our current computational resources [79]. Additionally, the VinDr-CXR dataset was excluded because of the rarity of certain pathologies, with some conditions represented by only a few hundred CXRs [80]. This limited sample size would have hindered a comprehensive analysis of the fairness dimension of the NPT classifiers across specific demographic groups. Future studies should consider incorporating more CXR datasets to enhance the generalizability of the findings in this study. Thirdly, while this study focuses on the tradeoffs between interpretability, fairness, and performance in interpretable NPT classifiers, incorporating vision transformers as the backbone of NPTs [81] and using self-supervised methods such as Dino-v2 for pretraining [82] may further enhance classifier performance in CXR pathology classification [83]. Future research should investigate these approaches to assess whether the relationships between performance, interpretability, and fairness shift under these conditions. Fourth, while we have presented both the global and local explanation of the NPT classifier and discussed their potential utility in aiding diagnostic procedures, their clinical significance needs to be established in a rigorous user study with radiologists. Such a study would involve evaluating whether the NPT accurately learns clinically relevant and causal features that align with a diagnostic process of the radiologist, as well as identifying potential failure modes of the global explanation pathways from a medical perspective. Fifth, the transferability of the NPT classifier across diverse clinical environments and unseen data distributions requires evaluation, as previous studies have highlighted the importance of this factor for ensuring its robustness and successful deployment in real-world settings [84,85]. Finally, it is crucial to investigate the appropriate way of integrating the NPT classifier into the workflow of radiologists. This involves conducting usability studies to better understand how the NPT classifier can effectively complement and enhance the existing diagnostic process [86].

### Conclusions

In this study, we have comprehensively investigated the NPT classifier’s performance, interpretability, and fairness dimensions in CXR pathology detection. Our findings demonstrated that the NPT classifier not only achieved competitive performance comparable to nontransparent deep learning classifiers but also offered the added benefit of providing faithful global and local explanations for its decision-making process. The traceability and interpretability provided by NPT classifiers represent considerable advancement toward enhancing transparency in the application of deep learning classifiers for CXR pathology detection. By shedding light on the complex relationship between performance, interpretability, and fairness in the NPT classifier, this research offers critical insights that could guide future advancement in effective, interpretable, and equitable deep learning innovations for CXR pathology detection.

## Appendix

Multimedia Appendix 1 Establishing a baseline for chest x-ray pathology detection with residual neural network-152.

Multimedia Appendix 2 Per-label performance of residual neural network-152 and neural prototype tree on chest x-ray 14, MIMIC-chest x-ray, and CheXpert.

Multimedia Appendix 3 Linear regression for investigating the impact of interpretation complexity level on area under the receiver operating characteristic curve.

Multimedia Appendix 4 Per-label true positive rate disparity for neural prototype tree classifiers with different interpretation complexity level.

Multimedia Appendix 5 Linear regression for investigating the impact of interpretation complexity level on mean true positive rate disparity.

Multimedia Appendix 6 Global explanations of neural prototype tree classifiers.

## Abbreviations

AI: artificial intelligence
CXR: chest X-ray
IC: interpretation complexity
NPT: neural prototype tree
ResNet: residual neural network
ROC AUC: area under the receiver operating characteristic curve
TPR: true positive rate
XAI: explainable artificial intelligence

## Data Availability

The data sets analyzed during this study are available in the PhysioNet repository (MIMIC-CXR [36]), the Stanford AIMI repository (CheXpert [35]), and the National Institutes of Health Clinical Center repository (Chest X-Ray 14 [12]).
