# Supplementary material for: Intersection of Performance, Interpretability, and Fairness in Neural Prototype Tree for Chest X-Ray Pathology Detection: Algorithm Development and Validation Study
Source: JMIR Form Res. 2024 Dec 5;8:e59045. doi: 10.2196/59045 (PMC11659703; doi:10.2196/59045)
Supplement: Multimedia Appendix 1 [file formative_v8i1e59045_app1.docx]

## Multimedia Appendix-1: Establishing a Baseline for CXR Pathology Detection with ResNet-152

Table 1 presents the ResNet-152’s ROC AUC on detecting CXR pathologies on the test dataset of Chest X-ray 14, along with the results of recent studies on the same dataset. The results have suggested that the performance of the ResNet-152 classifier, as measured by ROC AUC, aligns closely with the median performance across recent studies. The ResNet-152 outperformed the median performance of recent studies on twelve out of fourteen pathologies. Consequently, we select ResNet-152 as our baseline model and as the CNN backbone for our interpretable NPT model. By comparing our NPT model with the baseline ResNet-152, we can study the impact of interpretability on performance while eliminating the variation attributed to different CNN configurations.

Table 1. ResNet-152’s ROC AUC performance in detecting pathologies on the Chest X-ray 14 test dataset, compared to recent studies. The table presents ROC AUC values for fourteen pathologies (with abbreviations provided in brackets), highlighting ResNet-152’s performance alongside the performance of recent studies.Top of Form

Bottom of Form

| **Pathology** | **ResNet-152** | **[1]** | **[2]** | **[3]** | **[4]** | **[5]** | **[6]** | **[7]** |
| --- | --- | --- | --- | --- | --- | --- | --- | --- |
| Atelectasis (A) | 0.83 | 0.86 | 0.84 | 0.84 | 0.81 | 0.77 | 0.77 | 0.71 |
| Cardiomegaly (CD) | 0.88 | 0.83 | 0.92 | 0.88 | 0.92 | 0.87 | 0.86 | 0.80 |
| Consolidation(CO) | 0.82 | 0.89 | 0.81 | 0.86 | 0.80 | 0.74 | 0.73 | 0.70 |
| Edema(ED) | 0.91 | 0.92 | 0.91 | 0.92 | 0.92 | 0.84 | 0.83 | 0.83 |
| Effusion (EF) | 0.88 | 0.90 | 0.88 | 0.87 | 0.88 | 0.83 | 0.83 | 0.74 |
| Emphysema (EP) | 0.89 | 0.70 | 0.93 | 0.91 | 0.90 | 0.94 | 0.93 | 0.81 |
| Fibrosis (FB) | 0.82 | 0.81 | 0.78 | 0.84 | 0.79 | 0.83 | 0.82 | 0.76 |
| Hernia (HN) | 0.91 | 0.85 | 0.83 | 0.90 | 0.98 | 0.91 | 0.78 | 0.76 |
| Infiltration (IN) | 0.72 | 0.72 | 0.73 | 0.78 | 0.72 | 0.71 | 0.69 | 0.60 |
| Mass (M) | 0.87 | 0.91 | 0.87 | 0.85 | 0.83 | 0.83 | 0.81 | 0.70 |
| Nodule (ND) | 0.83 | 0.89 | 0.79 | 0.80 | 0.78 | 0.79 | 0.78 | 0.72 |
| Pleural Thickening (PT) | 0.81 | 0.80 | 0.81 | 0.89 | 0.81 | 0.79 | 0.79 | 0.70 |
| Pneumonia (PA) | 0.79 | 0.85 | 0.72 | 0.89 | 0.76 | 0.72 | 0.72 | 0.76 |
| Pneumothorax (PX) | 0.90 | 0.94 | 0.86 | 0.91 | 0.88 | 0.88 | 0.87 | 0.80  Top of Form  Bottom of Form |

1. Rajpurkar P, Irvin J, Ball RL, Zhu K, Yang B, Mehta H, Duan T, Ding D, Bagul A, Langlotz CP, Patel BN, Yeom KW, Shpanskaya K, Blankenberg FG, Seekins J, Amrhein TJ, Mong DA, Halabi SS, Zucker EJ, Ng AY, Lungren MP. Deep learning for chest radiograph diagnosis: A retrospective comparison of the CheXNeXt algorithm to practicing radiologists. PLOS Medicine Public Library of Science; 2018 Nov 20;15(11):e1002686. doi: 10.1371/journal.pmed.1002686

2. Cohen JP, Bertin P, Frappier V. Chester: A Web Delivered Locally Computed Chest X-Ray Disease Prediction System. arXiv.org. 2019. Available from: https://arxiv.org/abs/1901.11210v3 [accessed Jul 3, 2023]

3. Nie W, Zhang C, Song D, Bai Y, Xie K, Liu A. Instrumental Variable Learning for Chest X-ray Classification. arXiv.org. 2023. Available from: https://arxiv.org/abs/2305.12070v1 [accessed Jul 3, 2023]

4. Seyyed-Kalantari L, Zhang H, McDermott MBA, Chen IY, Ghassemi M. Underdiagnosis bias of artificial intelligence algorithms applied to chest radiographs in under-served patient populations. Nat Med Nature Publishing Group; 2021 Dec;27(12):2176–2182. doi: 10.1038/s41591-021-01595-0

5. Ouyang X, Karanam S, Wu Z, Chen T, Huo J, Zhou XS, Wang Q, Cheng J-Z. Learning Hierarchical Attention for Weakly-Supervised Chest X-Ray Abnormality Localization and Diagnosis. IEEE Transactions on Medical Imaging 2021 Oct;40(10):2698–2710. doi: 10.1109/TMI.2020.3042773

6. Ye W, Yao J, Xue H, Li Y. Weakly Supervised Lesion Localization With Probabilistic-CAM Pooling. arXiv; 2020. Available from: http://arxiv.org/abs/2005.14480 [accessed Jun 28, 2023]

7. Wang X, Peng Y, Lu L, Lu Z, Bagheri M, Summers RM. ChestX-ray8: Hospital-Scale Chest X-Ray Database and Benchmarks on Weakly-Supervised Classification and Localization of Common Thorax Diseases. :10.
