# Supplementary material for: Intersection of Performance, Interpretability, and Fairness in Neural Prototype Tree for Chest X-Ray Pathology Detection: Algorithm Development and Validation Study
Source: JMIR Form Res. 2024 Dec 5;8:e59045. doi: 10.2196/59045 (PMC11659703; doi:10.2196/59045)
Supplement: Multimedia Appendix 2 [file formative_v8i1e59045_app2.docx]

## Multimedia Appendix-2: Per-label Performance of ResNet-152 and NPT on Chest X-ray 14, MIMIC-CXR, and CheXpert

The ROC AUC performance of ResNet-152 and NPT classifiers, across varying levels of IC, is presented in Table 1 and Table 2. The results indicate a consistent trend: as the IC level increases, the performance of the NPT classifier improves. For PA view chest X-rays in the Chest X-ray 14 dataset, the NPT surpassed the performance of ResNet-152 at IC=15 for five pathologies and at IC=31 for seven pathologies. In the CheXpert dataset, the NPT outperformed ResNet-152 at IC=15 for four pathologies and at IC=31 for six pathologies. In the MIMIC-CXR dataset, the NPT outperformed ResNet-152 at IC=15 for four pathologies and at IC=31 for five pathologies. Similarly, for AP view chest X-rays in Chest X-ray 14, the NPT outperformed ResNet-152 at IC=15 for seven pathologies and at IC=31 for six pathologies. In CheXpert, the NPT exceeded ResNet-152's performance at IC=15 for six pathologies, and at IC=31 for six pathologies. In MIMIC-CXR, the NPT outperformed ResNet-152’s performance at IC=15 for four pathologies, and at IC=31 for five pathologies.

Table 1. Performance comparison (ROC AUC) of ResNet-152 and NPT classifiers across different interpretation complexity (IC) levels for detecting various pathologies in Chest X-ray 14, CheXpert and MIMIC-CXR datasets (PA view). Performance generally improves for NPT as IC increases, with IC=15 and IC=31 frequently surpassing ResNet-152 in multiple pathologies. Bolded values indicate IC level where NPT outperforms ResNet-152.

| **Chest X-ray 14 (PA view)** | **ResNet-152** | **NPT_IC=1** | **NPT_IC=3** | **NPT_IC=7** | **NPT_IC=15** | **NPT_IC=31** |
| --- | --- | --- | --- | --- | --- | --- |
| **Pathology** |  |  |  |  |  |  |
| Atelectasis (A) | 0.834 | 0.763 ± 0.005 | 0.786 ± 0.003 | 0.813 ± 0.004 | 0.832 ± 0.002 | **0.837 ± 0.003** |
| Cardiomegaly (CD) | 0.882 | 0.804 ± 0.004 | 0.829 ± 0.003 | 0.866 ± 0.003 | 0.879 ± 0.002 | **0.882 ± 0.002** |
| Consolidation (CO) | 0.819 | 0.758 ± 0.005 | 0.784 ± 0.004 | 0.801 ± 0.002 | **0.821 ± 0.004** | 0.822 ± 0.003 |
| Edema (ED) | 0.907 | 0.839 ± 0.003 | 0.864 ± 0.002 | 0.895 ± 0.003 | 0.907 ± 0.004 | 0.908 ± 0.003 |
| Effusion (EF) | 0.882 | 0.822 ± 0.004 | 0.848 ± 0.002 | 0.869 ± 0.002 | **0.883 ± 0.003** | 0.889 ± 0.003 |
| Emphysema (EP) | 0.894 | 0.827 ± 0.005 | 0.845 ± 0.004 | 0.877 ± 0.002 | **0.897 ± 0.002** | 0.899 ± 0.003 |
| Fibrosis (FB) | 0.823 | 0.753 ± 0.004 | 0.773 ± 0.003 | 0.802 ± 0.003 | 0.822 ± 0.003 | **0.824 ± 0.002** |
| Hernia (HN) | 0.911 | 0.858 ± 0.003 | 0.871 ± 0.002 | 0.904 ± 0.003 | 0.913 ± 0.003 | **0.917 ± 0.002** |
| Infiltration (IN) | 0.72 | 0.651 ± 0.005 | 0.679 ± 0.003 | 0.706 ± 0.002 | **0.722 ± 0.002** | 0.730 ± 0.003 |
| Mass (M) | 0.869 | 0.793 ± 0.004 | 0.816 ± 0.003 | 0.853 ± 0.003 | 0.869 ± 0.002 | **0.877 ± 0.002** |
| Nodule (ND) | 0.826 | 0.757 ± 0.003 | 0.770 ± 0.004 | 0.807 ± 0.003 | **0.828 ± 0.002** | 0.833 ± 0.002 |
| Pleural Thickening (PT) | 0.813 | 0.739 ± 0.005 | 0.756 ± 0.004 | 0.792 ± 0.003 | 0.810 ± 0.003 | **0.811 ± 0.003** |
| Pneumonia (PA) | 0.793 | 0.725 ± 0.005 | 0.748 ± 0.003 | 0.773 ± 0.003 | 0.782 ± 0.003 | **0.798 ± 0.002** |
| Pneumothorax (PX) | 0.897 | 0.821 ± 0.003 | 0.868 ± 0.002 | **0.899 ± 0.003** | 0.906 ± 0.003 | 0.909 ± 0.002 |
| **CheXpert (PA view)** |  |  |  |  |  |  |
| **Pathology** |  |  |  |  |  |  |
| Atelectasis (A) | 0.731 | 0.673 ± 0.005 | 0.682 ± 0.004 | 0.726 ± 0.003 | 0.730 ± 0.002 | **0.731 ± 0.002** |
| Cardiomegaly (CD) | 0.863 | 0.794 ± 0.004 | 0.811 ± 0.003 | 0.851 ± 0.003 | **0.865 ± 0.002** | 0.877 ± 0.002 |
| Consolidation (CO) | 0.756 | 0.699 ± 0.005 | 0.715 ± 0.004 | 0.747 ± 0.003 | **0.759 ± 0.002** | 0.763 ± 0.003 |
| Edema (ED) | 0.871 | 0.801 ± 0.004 | 0.824 ± 0.003 | 0.852 ± 0.002 | 0.871 ± 0.003 | 0.872 ± 0.002 |
| Enlarged Cardio Mediastinum (EC) | 0.706 | 0.633 ± 0.005 | 0.657 ± 0.004 | 0.696 ± 0.003 | 0.703 ± 0.002 | 0.707 ± 0.002 |
| Fracture (FR) | 0.813 | 0.745 ± 0.005 | 0.761 ± 0.003 | 0.791 ± 0.002 | 0.813 ± 0.003 | **0.818 ± 0.003** |
| Lung Lesion (LL) | 0.803 | 0.731 ± 0.005 | 0.758 ± 0.004 | 0.785 ± 0.003 | 0.801 ± 0.002 | **0.803 ± 0.003**  Top of Form  Bottom of Form |
| Lung Opacity (LO) | 0.759 | 0.698 ± 0.005 | 0.719 ± 0.004 | 0.743 ± 0.003 | **0.762 ± 0.002** | 0.772 ± 0.003 |
| Pleural Effusion (PE) | 0.874 | 0.807 ± 0.004 | 0.823 ± 0.003 | 0.856 ± 0.002 | 0.871 ± 0.003 | **0.878 ± 0.002** |
| Pleural Other (PO) | 0.807 | 0.742 ± 0.004 | 0.764 ± 0.003 | 0.796 ± 0.002 | 0.805 ± 0.002 | **0.816 ± 0.003** |
| Pneumonia (PA) | 0.793 | 0.729 ± 0.005 | 0.741 ± 0.003 | 0.772 ± 0.003 | 0.786 ± 0.003 | 0.792 ± 0.003 |
| Pneumothorax (PX) | 0.884 | 0.812 ± 0.003 | 0.832 ± 0.002 | 0.877 ± 0.003 | **0.881 ± 0.002** | 0.893 ± 0.002 |
| Support Devices (SD) | 0.912 | 0.840 ± 0.004 | 0.868 ± 0.003 | 0.903 ± 0.002 | 0.912 ± 0.003 | **0.917 ± 0.003** |
| **MIMIC-CXR (PA view)** | | | | | | |
| **Pathology** |  |  |  |  |  |  |
| Airspace Opacity (AO) | 0.803 | 0.713 ± 0.002 | 0.747 ± 0.005 | 0.771 ± 0.004 | 0.793 ± 0.003 | **0.806 ± 0.002** |
| Atelectasis (A) | 0.829 | 0.709 ± 0.002 | 0.732 ± 0.001 | 0.756 ± 0.004 | 0.797 ± 0.003 | 0.814 ± 0.004 |
| Cardiomegaly (CD) | 0.830 | 0.735 ± 0.001 | 0.748 ± 0.005 | 0.772 ± 0.004 | 0.808 ± 0.002 | **0.833 ± 0.002** |
| Consolidation (CO) | 0.857 | 0.719 ± 0.002 | 0.745 ± 0.002 | 0.786 ± 0.003 | 0.814 ± 0.003 | 0.849 ± 0.002 |
| Edema (ED) | 0.914 | 0.848 ± 0.003 | 0.859 ± 0.002 | 0.876 ± 0.002 | **0.916 ± 0.002** | 0.923 ± 0.003 |
| Enlarged Cardio Mediastinum (EC) | 0.768 | 0.672 ± 0.004 | 0.693 ± 0.002 | 0.735 ± 0.003 | 0.761 ± 0.003 | **0.784 ± 0.001** |
| Fracture (FR) | 0.735 | 0.655 ± 0.003 | 0.682 ± 0.002 | 0.716 ± 0.001 | **0.741 ± 0.005** | 0.749 ± 0.005 |
| Lung Lesion (LL) | 0.756 | 0.621 ± 0.004 | 0.658 ± 0.002 | 0.683 ± 0.001 | 0.726 ± 0.004 | 0.749 ± 0.003 |
| Pleural Effusion (PE) | 0.915 | 0.815 ± 0.001 | 0.837 ± 0.003 | 0.857 ± 0.001 | 0.887 ± 0.005 | **0.918 ± 0.002** |
| Pleural Other (PO) | 0.837 | 0.732 ± 0.004 | 0.776 ± 0.002 | 0.821 ± 0.003 | **0.839 ± 0.003** | 0.852 ± 0.002 |
| Pneumonia (PA) | 0.768 | 0.687 ± 0.005 | 0.713 ± 0.004 | 0.736 ± 0.005 | **0.772 ± 0.005** | 0.784 ± 0.003 |
| Pneumothorax (PX) | 0.894 | 0.805 ± 0.005 | 0.836 ± 0.001 | 0.875 ± 0.002 | 0.890 ± 0.001 | **0.908 ± 0.002** |
| Support Devices (SD) | 0.920 | 0.839 ± 0.003 | 0.842 ± 0.002 | 0.867 ± 0.004 | 0.897 ± 0.002 | 0.913 ± 0.002 |

Table 2. Performance comparison (ROC AUC) of ResNet-152 and NPT classifiers across different interpretation complexity levels for detecting various pathologies in Chest X-ray 14, CheXpert, and MIMIC-CXR datasets (AP view). The NPT classifier demonstrates improved performance as the IC level increases, frequently matching or exceeding ResNet-152's performance, particularly at IC=15 and IC=31. Bolded values indicate IC level where NPT outperforms ResNet-152.

| **Chest X-ray 14 (AP view)** | **ResNet-152** | **NPT_IC=1** | **NPT_IC=3** | **NPT_IC=7** | **NPT_IC=15** | **NPT_IC=31** |
| --- | --- | --- | --- | --- | --- | --- |
| **Pathology** |  |  |  |  |  |  |
| Atelectasis (A) | 0.834 | 0.748 ± 0.005 | 0.767 ± 0.004 | 0.807 ± 0.003 | 0.834 ± 0.003 | **0.836 ± 0.002** |
| Cardiomegaly (CD) | 0.882 | 0.797 ± 0.004 | 0.829 ± 0.003 | 0.851 ± 0.003 | **0.885 ± 0.002** | 0.889 ± 0.003 |
| Consolidation (CO) | 0.819 | 0.746 ± 0.005 | 0.763 ± 0.004 | 0.784 ± 0.002 | 0.810 ± 0.003 | 0.818 ± 0.002 |
| Edema (ED) | 0.907 | 0.821 ± 0.003 | 0.846 ± 0.002 | 0.886 ± 0.003 | 0.892 ± 0.003 | **0.908 ± 0.002** |
| Effusion (EF) | 0.882 | 0.818 ± 0.004 | 0.831 ± 0.003 | 0.860 ± 0.003 | **0.883 ± 0.003** | 0.885 ± 0.002 |
| Emphysema (EP) | 0.894 | 0.817 ± 0.005 | 0.832 ± 0.004 | 0.854 ± 0.003 | 0.891 ± 0.002 | **0.896 ± 0.003** |
| Fibrosis (FB) | 0.823 | 0.743 ± 0.005 | 0.765 ± 0.003 | 0.805 ± 0.003 | **0.826 ± 0.002** | 0.835 ± 0.002 |
| Hernia (HN) | 0.911 | 0.837 ± 0.003 | 0.869 ± 0.002 | 0.891 ± 0.003 | **0.915 ± 0.003** | 0.921 ± 0.002 |
| Infiltration (IN) | 0.72 | 0.669 ± 0.005 | 0.673 ± 0.003 | 0.703 ± 0.003 | **0.723 ± 0.002** | 0.732 ± 0.003 |
| Mass (M) | 0.869 | 0.782 ± 0.004 | 0.806 ± 0.003 | 0.847 ± 0.003 | 0.869 ± 0.003 | **0.873 ± 0.002** |
| Nodule (ND) | 0.826 | 0.753 ± 0.003 | 0.771 ± 0.004 | 0.809 ± 0.003 | **0.828 ± 0.002** | 0.834 ± 0.002 |
| Pleural Thickening (PT) | 0.813 | 0.725 ± 0.005 | 0.754 ± 0.004 | 0.793 ± 0.003 | **0.816 ± 0.003** | 0.819 ± 0.003 |
| Pneumonia (PA) | 0.793 | 0.728 ± 0.005 | 0.748 ± 0.003 | 0.772 ± 0.003 | 0.788 ± 0.003 | **0.803 ± 0.002** |
| Pneumothorax (PX) | 0.897 | 0.826 ± 0.003 | 0.895 ± 0.002 | 0.879 ± 0.003 | 0.896 ± 0.003 | **0.906 ± 0.002** |
| **CheXpert (AP view)** |  |  |  |  |  |  |
| **Pathology** |  |  |  |  |  |  |
| Atelectasis (A) | 0.731 | 0.663 ± 0.005 | 0.689 ± 0.004 | 0.711 ± 0.003 | **0.735 ± 0.003** | 0.741 ± 0.002 |
| Cardiomegaly (CD) | 0.863 | 0.787 ± 0.004 | 0.805 ± 0.003 | 0.853 ± 0.003 | **0.865 ± 0.002** | 0.869 ± 0.002 |
| Consolidation (CO) | 0.756 | 0.698 ± 0.005 | 0.700 ± 0.004 | 0.736 ± 0.003 | **0.758 ± 0.002** | 0.762 ± 0.003 |
| Edema (ED) | 0.871 | 0.802 ± 0.004 | 0.823 ± 0.003 | 0.864 ± 0.002 | 0.870 ± 0.003 | **0.873 ± 0.002** |
| Enlarged Cardio Mediastinum (EC) | 0.706 | 0.637 ± 0.005 | 0.652 ± 0.004 | 0.695 ± 0.003 | **0.709 ± 0.002** | 0.711 ± 0.002 |
| Fracture (FR) | 0.813 | 0.741 ± 0.005 | 0.767 ± 0.003 | 0.796 ± 0.002 | **0.814 ± 0.003** | 0.816 ± 0.003 |
| Lung Lesion (LL) | 0.803 | 0.734 ± 0.005 | 0.752 ± 0.004 | 0.786 ± 0.003 | 0.801 ± 0.002 | **0.807 ± 0.003**  Top of Form  Bottom of Form |
| Lung Opacity (LO) | 0.759 | 0.697 ± 0.005 | 0.718 ± 0.004 | 0.747 ± 0.003 | 0.759 ± 0.002 | **0.761 ± 0.003** |
| Pleural Effusion (PE) | 0.874 | 0.809 ± 0.004 | 0.823 ± 0.003 | 0.852 ± 0.002 | **0.877 ± 0.003** | 0.879 ± 0.002 |
| Pleural Other (PO) | 0.807 | 0.746 ± 0.004 | 0.765 ± 0.003 | 0.796 ± 0.002 | 0.804 ± 0.002 | **0.819 ± 0.003** |
| Pneumonia (PA) | 0.793 | 0.721 ± 0.005 | 0.748 ± 0.003 | 0.773 ± 0.003 | 0.786 ± 0.003 | **0.795 ± 0.003** |
| Pneumothorax (PX) | 0.884 | 0.815 ± 0.003 | 0.832 ± 0.002 | 0.868 ± 0.003 | 0.879 ± 0.002 | 0.883 ± 0.002 |
| Support Devices (SD) | 0.912 | 0.843 ± 0.004 | 0.862 ± 0.003 | 0.893 ± 0.002 | 0.907 ± 0.003 | **0.912 ± 0.003** |
| **MIMIC-CXR (AP view)** |  |  |  |  |  |  |
| **Pathology** |  |  |  |  |  |  |
| Airspace Opacity (AO) | 0.803 | 0.736 ± 0.003 | 0.756 ± 0.002 | 0.779 ± 0.001 | **0.811 ± 0.002** | 0.819 ± 0.005 |
| Atelectasis (A) | 0.829 | 0.705 ± 0.002 | 0.736 ± 0.003 | 0.769 ± 0.004 | 0.806 ± 0.002 | **0.834 ± 0.005** |
| Cardiomegaly (CD) | 0.830 | 0.748 ± 0.005 | 0.774 ± 0.002 | 0.803 ± 0.003 | 0.826 ± 0.002 | **0.837 ± 0.002** |
| Consolidation (CO) | 0.857 | 0.729 ± 0.001 | 0.749 ± 0.003 | 0.786 ± 0.003 | 0.815 ± 0.001 | 0.839 ± 0.002 |
| Edema (ED) | 0.914 | 0.786 ± 0.005 | 0.827 ± 0.002 | 0.854 ± 0.002 | 0.883 ± 0.003 | 0.906 ± 0.005 |
| Enlarged Cardio Mediastinum (EC) | 0.768 | 0.665 ± 0.002 | 0.697 ± 0.004 | 0.747 ± 0.004 | **0.771 ± 0.002** | 0.785 ± 0.004 |
| Fracture (FR) | 0.735 | 0.671 ± 0.002 | 0.693 ± 0.004 | 0.718 ± 0.004 | 0.729 ± 0.003 | **0.743 ± 0.001** |
| Lung Lesion (LL) | 0.756 | 0.640 ± 0.004 | 0.658 ± 0.002 | 0.673 ± 0.002 | 0.713 ± 0.001 | 0.749 ± 0.003 |
| Pleural Effusion (PE) | 0.915 | 0.812 ± 0.004 | 0.856 ± 0.001 | 0.884 ± 0.003 | **0.919 ± 0.002** | 0.927 ± 0.004 |
| Pleural Other (PO) | 0.837 | 0.708 ± 0.002 | 0.732 ± 0.004 | 0.775 ± 0.003 | 0.812 ± 0.005 | **0.845 ± 0.002** |
| Pneumonia (PA) | 0.768 | 0.675 ± 0.002 | 0.725 ± 0.001 | 0.738 ± 0.005 | **0.776 ± 0.005** | 0.785 ± 0.002 |
| Pneumothorax (PX) | 0.894 | 0.772 ± 0.004 | 0.829 ± 0.004 | 0.863 ± 0.003 | 0.885 ± 0.003 | **0.907 ± 0.002** |
| Support Devices (SD) | 0.920 | 0.804 ± 0.001 | 0.836 ± 0.005 | 0.863 ± 0.005 | 0.897 ± 0.004 | 0.905 ± 0.002 |
