# Supplementary material for: Intersection of Performance, Interpretability, and Fairness in Neural Prototype Tree for Chest X-Ray Pathology Detection: Algorithm Development and Validation Study
Source: JMIR Form Res. 2024 Dec 5;8:e59045. doi: 10.2196/59045 (PMC11659703; doi:10.2196/59045)
Supplement: Multimedia Appendix 3 [file formative_v8i1e59045_app3.docx]

## Multimedia Appendix-3: Linear Regression for Investigating the Impact of IC Level on ROC AUC

Table 1. Linear regression results analyzing the effect of IC level on ROC AUC across various pathologies for PA view CXRs in Chest X-ray 14 dataset. Each model is based on 25 data points, comprising five IC levels with five experimental runs for each level. The table reports the coefficient for IC level, standard error, t-statistic, adjusted R-squared, F-statistic, sample size, and p-value. The corrected p-values were calculated using the Benjamini-Hochberg procedure to control for the false discovery rate.

| **Pathology** | **Coefficient (IC Level)** | **Std. Error** | **t-statistic** | **Adjusted R-squared** | **F-statistic** | **Sample Size** | **p-value** | | **corrected p-value** |
| --- | --- | --- | --- | --- | --- | --- | --- | --- | --- |
| Atelectasis | 2.15E-03 | 2.90E-04 | 7.414 | 0.6922 | 54.97 | 25 | | < .001 | < .001 |
| Cardiomegaly | 2.20E-03 | 3.59E-04 | 6.115 | 0.6026 | 37.39 | 25 | | < .001 | < .001 |
| Consolidation | 1.77E-03 | 2.74E-04 | 6.453 | 0.6287 | 41.64 | 25 | | < .001 | < .001 |
| Edema | 1.91E-03 | 3.30E-04 | 5.798 | 0.5761 | 33.62 | 25 | | < .001 | < .001 |
| Effusion | 1.85E-03 | 2.65E-04 | 6.99 | 0.666 | 48.86 | 25 | | < .001 | < .001 |
| Emphysema | 2.17E-03 | 2.98E-04 | 7.259 | 0.6829 | 52.69 | 25 | | < .001 | < .001 |
| Fibrosis | 2.09E-03 | 2.95E-04 | 7.105 | 0.6734 | 50.49 | 25 | | < .001 | < .001 |
| Hernia | 1.79E-03 | 2.60E-04 | 6.86 | 0.6575 | 47.06 | 25 | | < .001 | < .001 |
| Infiltration | 2.23E-03 | 3.02E-04 | 7.38 | 0.6902 | 54.46 | 25 | | < .001 | < .001 |
| Mass | 2.45E-03 | 3.44E-04 | 7.121 | 0.6744 | 50.71 | 25 | | < .001 | < .001 |
| Nodule | 2.35E-03 | 3.03E-04 | 7.769 | 0.7121 | 60.35 | 25 | | < .001 | < .001 |
| Pleural Thickening | 2.19E-03 | 3.30E-04 | 6.626 | 0.6413 | 43.91 | 25 | | < .001 | < .001 |
| Pneumonia | 2.04E-03 | 2.33E-04 | 8.763 | 0.7595 | 76.78 | 25 | | < .001 | < .001 |
| Pneumothorax | 2.18E-03 | 4.51E-04 | 4.832 | 0.4822 | 23.35 | 25 | | < .001 | < .001 |

Table 2. Linear regression results analyzing the effect of IC level on ROC AUC across various pathologies for PA view CXRs in CheXpert dataset. Each model is based on 25 data points, comprising five IC levels with five experimental runs for each level. The table reports the coefficient for IC level, standard error, t-statistic, adjusted R-squared, F-statistic, sample size, and p-value. The corrected p-values were calculated using the Benjamini-Hochberg procedure to control for the false discovery rate.

| **Pathology** | **Coefficient**  **(IC Level)** | **Std. Error** | **t-statistic** | **Adjusted R-squared** | **F-statistic** | **Sample Size** | **p-value** | **corrected p-value** |
| --- | --- | --- | --- | --- | --- | --- | --- | --- |
| Atelectasis | 8.80E-04 | 1.53E-04 | 5.75 | 0.5719 | 33.06 | 25 | < .001 | < .001 |
| Cardiomegaly | 1.22E-03 | 1.43E-04 | 8.51 | 0.7483 | 72.35 | 25 | < .001 | < .001 |
| Consolidation | 9.31E-04 | 1.29E-04 | 7.21 | 0.6802 | 52.05 | 25 | < .001 | < .001 |
| Edema | 1.04E-03 | 1.39E-04 | 7.44 | 0.6939 | 55.40 | 25 | < .001 | < .001 |
| Enlarged Cardio Mediastinum | 1.03E-03 | 1.63E-04 | 6.32 | 0.6183 | 39.88 | 25 | < .001 | < .001 |
| Fracture | 1.11E-03 | 1.27E-04 | 8.74 | 0.7586 | 76.41 | 25 | < .001 | < .001 |
| Lung Lesion | 9.97E-04 | 1.45E-04 | 6.9 | 0.6598 | 47.55 | 25 | < .001 | < .001 |
| Lung Opacity | 1.08E-03 | 1.15E-04 | 9.43 | 0.7855 | 88.90 | 25 | < .001 | < .001 |
| Pleural Effusion | 1.05E-03 | 1.26E-04 | 8.36 | 0.7416 | 69.87 | 25 | < .001 | < .001 |
| Pleural Other | 1.05E-03 | 1.29E-04 | 8.12 | 0.7301 | 65.93 | 25 | < .001 | < .001 |
| Pneumonia | 9.61E-04 | 1.16E-04 | 8.31 | 0.7393 | 69.05 | 25 | < .001 | < .001 |
| Pneumothorax | 1.16E-03 | 1.58E-04 | 7.34 | 0.6876 | 53.83 | 25 | < .001 | < .001 |
| Support Devices | 1.05E-03 | 1.62E-04 | 6.48 | 0.6309 | 42.02 | 25 | < .001 | < .001 |

Table 3. Linear regression results analyzing the effect of IC level on ROC AUC across various pathologies for PA view CXRs in the MIMIC-CXR dataset. Each model is based on 25 data points, comprising five IC levels with five experimental runs for each level. The table reports the coefficient for IC level, standard error, t-statistic, adjusted R-squared, F-statistic, sample size, and p-value. The corrected p-values were calculated using the Benjamini-Hochberg procedure to control for the false discovery rate.

| **Pathology** | **Coefficient**  **(IC Level)** | **Std. Error** | **t-statistic** | **Adjusted R-squared** | **F-statistic** | **Sample Size** | **p-value** | **corrected p-value** |
| --- | --- | --- | --- | --- | --- | --- | --- | --- |
| Airspace Opacity | 2.62E-03 | 3.19E-04 | 8.22 | 0.7351 | 67.59 | 25 | < .001 | < .001 |
| Atelectasis | 3.31E-03 | 2.88E-04 | 8.49 | 0.7451 | 69.05 | 25 | < .001 | < .001 |
| Cardiomegaly | 3.19E-03 | 2.03E-04 | 9.74 | 0.7114 | 68.83 | 25 | < .001 | < .001 |
| Consolidation | 3.96E-03 | 3.17E-04 | 7.52 | 0.7665 | 62.36 | 25 | < .001 | < .001 |
| Edema | 2.51E-03 | 2.40E-04 | 8.47 | 0.7190 | 86.78 | 25 | < .001 | < .001 |
| Enlarged Cardio Mediastinum | 3.46E-03 | 3.29E-04 | 9.51 | 0.7203 | 95.76 | 25 | < .001 | < .001 |
| Fracture | 2.77E-03 | 3.60E-04 | 7.70 | 0.7083 | 59.29 | 25 | < .001 | < .001 |
| Lung Lesion | 3.86E-03 | 3.44E-04 | 8.39 | 0.7388 | 75.93 | 25 | < .001 | < .001 |
| Pleural Effusion | 3.19E-03 | 1.87E-04 | 7.05 | 0.6836 | 43.25 | 25 | < .001 | < .001 |
| Pleural Other | 3.31E-03 | 4.83E-04 | 6.86 | 0.6579 | 47.06 | 25 | < .001 | < .001 |
| Pneumonia | 3.01E-03 | 2.98E-04 | 8.03 | 0.7041 | 53.02 | 25 | < .001 | < .001 |
| Pneumothorax | 2.96E-03 | 3.70E-04 | 7.99 | 0.7236 | 63.82 | 25 | < .001 | < .001 |
| Support Devices | 2.85E-03 | 1.56E-04 | 8.32 | 0.7531 | 72.34 | 25 | < .001 | < .001 |

Table 4. Linear regression results analyzing the effect of IC level on ROC AUC across various pathologies for AP view CXRs in Chest X-ray 14 dataset. Each model is based on 25 data points, comprising five IC levels with five experimental runs for each level. The table reports the coefficient for IC level, standard error, t-statistic, adjusted R-squared, F-statistic, sample size, and p-value. The corrected p-values were calculated using the Benjamini-Hochberg procedure to control for the false discovery rate.

| **Pathology** | **Coefficient (IC Level)** | **Std. Error** | **t-statistic** | **Adjusted R-squared** | **F-statistic** | **p-value** | **Corrected p-vale** |  |
| --- | --- | --- | --- | --- | --- | --- | --- | --- |
| Atelectasis | 2.71E-03 | 3.76E-04 | 7.22 | 0.681 | 52.19 | < .001 | < .001 |  |
| Cardiomegaly | 2.72E-03 | 3.44E-04 | 7.91 | 0.719 | 62.51 | < .001 | < .001 |  |
| Consolidation | 2.23E-03 | 2.33E-04 | 9.6 | 0.791 | 92.08 | < .001 | < .001 |  |
| Edema | 2.46E-03 | 3.38E-04 | 7.29 | 0.685 | 53.17 | < .001 | < .001 |  |
| Effusion | 2.11E-03 | 2.73E-04 | 7.71 | 0.709 | 59.51 | < .001 | < .001 |  |
| Emphysema | 2.56E-03 | 2.70E-04 | 9.49 | 0.788 | 90.1 | < .001 | < .001 |  |
| Fibrosis | 2.76E-03 | 3.55E-04 | 7.78 | 0.713 | 60.51 | < .001 | < .001 |  |
| Hernia | 2.39E-03 | 3.19E-04 | 7.48 | 0.696 | 56.01 | < .001 | < .001 |  |
| Infiltration | 2.09E-03 | 2.18E-04 | 9.62 | 0.792 | 92.47 | < .001 | < .001 |  |
| Mass | 2.70E-03 | 3.87E-04 | 6.99 | 0.666 | 48.87 | < .001 | < .001 |  |
| Nodule | 2.45E-03 | 3.30E-04 | 7.44 | 0.693 | 55.29 | < .001 | < .001 |  |
| Pleural Thickening | 2.73E-03 | 4.04E-04 | 6.77 | 0.651 | 45.8 | < .001 | < .001 |  |
| Pneumonia | 2.23E-03 | 2.26E-04 | 9.85 | 0.8 | 96.96 | < .001 | < .001 |  |
| Pneumothorax | 1.69E-03 | 4.16E-04 | 4.06 | 0.393 | 16.51 | < .001 | < .001 |  |

Table 5. Linear regression results analyzing the effect of IC level on ROC AUC across various pathologies for AP view CXRs in CheXpert dataset. Each model is based on 25 data points, comprising five IC levels with five experimental runs for each level. The table reports the coefficient for IC level, standard error, t-statistic, adjusted R-squared, F-statistic, sample size, and p-value. The corrected p-values were calculated using the Benjamini-Hochberg procedure to control for the false discovery rate.

| **Pathology** | **Coefficient (IC Level)** | **Std. Error** | **t-statistic** | **Adjusted R-squared** | **F-statistic** | **p-value** | **Corrected**  **p-value** |
| --- | --- | --- | --- | --- | --- | --- | --- |
| Atelectasis | 2.28E-03 | 2.89E-04 | 7.89 | 0.719 | 62.3 | < .001 | < .001 |
| Cardiomegaly | 2.40E-03 | 3.84E-04 | 6.26 | 0.614 | 39.23 | < .001 | < .001 |
| Consolidation | 2.14E-03 | 2.69E-04 | 7.96 | 0.722 | 63.28 | < .001 | < .001 |
| Edema | 1.97E-03 | 3.56E-04 | 5.54 | 0.553 | 30.67 | < .001 | < .001 |
| Enlarged Cardio Mediastinum | 2.23E-03 | 3.46E-04 | 6.45 | 0.628 | 41.59 | < .001 | < .001 |
| Fracture | 2.16E-03 | 3.25E-04 | 6.63 | 0.642 | 43.97 | < .001 | < .001 |
| Lung Lesion | 2.17E-03 | 3.02E-04 | 7.18 | 0.678 | 51.57 | < .001 | < .001 |
| Lung Opacity | 1.81E-03 | 2.87E-04 | 6.32 | 0.619 | 39.99 | < .001 | < .001 |
| Pleural Effusion | 2.19E-03 | 2.82E-04 | 7.76 | 0.712 | 60.28 | < .001 | < .001 |
| Pleural Other | 2.11E-03 | 2.56E-04 | 8.24 | 0.736 | 67.91 | < .001 | < .001 |
| Pneumonia | 2.07E-03 | 2.84E-04 | 7.27 | 0.684 | 52.91 | < .001 | < .001 |
| Pneumothorax | 1.99E-03 | 3.09E-04 | 6.44 | 0.628 | 41.48 | < .001 | < .001 |
| Support Devices | 2.01E-03 | 2.89E-04 | 6.95 | 0.664 | 48.32 | < .001 | < .001 |

Table 6. Linear regression results analyzing the effect of IC level on ROC AUC across various pathologies for AP view CXRs in the MIMIC-CXR dataset. Each model is based on 25 data points, comprising five IC levels with five experimental runs for each level. The table reports the coefficient for IC level, standard error, t-statistic, adjusted R-squared, F-statistic, sample size, and p-value. The corrected p-values were calculated using the Benjamini-Hochberg procedure to control for the false discovery rate.

| **Pathology** | **Coefficient**  **(IC Level)** | **Std. Error** | **t-statistic** | **Adjusted R-squared** | **F-statistic** | **Sample Size** | **p-value** | **corrected p-value** |
| --- | --- | --- | --- | --- | --- | --- | --- | --- |
| Airspace Opacity | 2.56E-03 | 2.77E-04 | 8.27 | 0.7798 | 76.23 | 25 | < .001 | < .001 |
| Atelectasis | 3.92E-03 | 3.33E-04 | 7.36 | 0.6825 | 58.25 | 25 | < .001 | < .001 |
| Cardiomegaly | 2.64E-03 | 3.12E-04 | 6.48 | 0.6247 | 43.62 | 25 | < .001 | < .001 |
| Consolidation | 3.41E-03 | 2.95E-04 | 5.54 | 0.5536 | 34.67 | 25 | < .001 | < .001 |
| Edema | 3.23E-03 | 3.59E-04 | 6.58 | 0.6327 | 43.19 | 25 | < .001 | < .001 |
| Enlarged Cardio Mediastinum | 3.54E-03 | 4.51E-04 | 7.85 | 0.7164 | 61.63 | 25 | < .001 | < .001 |
| Fracture | 2.04E-03 | 2.42E-04 | 8.41 | 0.7438 | 70.68 | 25 | < .001 | < .001 |
| Lung Lesion | 3.54E-03 | 3.58E-04 | 7.29 | 0.6635 | 41.75 | 25 | < .001 | < .001 |
| Pleural Effusion | 3.25E-03 | 4.33E-04 | 7.51 | 0.6978 | 56.41 | 25 | < .001 | < .001 |
| Pleural Other | 4.30E-03 | 3.42E-04 | 6.53 | 0.6319 | 46.72 | 25 | < .001 | < .001 |
| Pneumonia | 3.04E-03 | 4.01E-04 | 7.58 | 0.7015 | 57.41 | 25 | < .001 | < .001 |
| Pneumothorax | 3.65E-03 | 4.89E-04 | 7.45 | 0.6944 | 55.54 | 25 | < .001 | < .001 |
| Support Devices | 3.03E-03 | 3.61E-04 | 8.39 | 0.7428 | 70.32 | 25 | < .001 | < .001 |
