# Supplementary material for: Intersection of Performance, Interpretability, and Fairness in Neural Prototype Tree for Chest X-Ray Pathology Detection: Algorithm Development and Validation Study
Source: JMIR Form Res. 2024 Dec 5;8:e59045. doi: 10.2196/59045 (PMC11659703; doi:10.2196/59045)
Supplement: Multimedia Appendix 4 [file formative_v8i1e59045_app4.docx]

## Multimedia Appendix-4: Per-label TPR Disparity for NPT Classifiers with Different IC Level

Table 1. Overview of true positive rate (TPR) disparities for NPT classifiers across subgroups differentiated by sex, age, and race for the CheXpert dataset. The table presents the TPR disparities for each subgroup across five IC levels (IC=1, IC=3, IC=7, IC=15, IC=31). Disparities are calculated by comparing subgroup performance within each IC level, where a positive TPR disparity indicates better performance for that group and a negative TPR disparity indicates worse performance.

|  |  | **IC=1** | **IC=3** | **IC=7** | **IC=15** | **IC=31** |
| --- | --- | --- | --- | --- | --- | --- |
| **Pathology** | **Subgroup** | **TPR Disparity** | **TPR Disparity** | **TPR Disparity** | **TPR Disparity** | **TPR Disparity** |
| Atelectasis | Female | -0.064 | -0.046 | -0.032 | -0.025 | -0.014 |
|  | Male | 0.064 | 0.046 | 0.032 | 0.025 | 0.014 |
|  | 0-25 | -0.078 | -0.054 | -0.039 | -0.027 | -0.017 |
|  | 26-50 | 0.031 | 0.021 | -0.044 | 0.009 | 0.005 |
|  | 51-75 | -0.024 | -0.016 | 0.068 | 0.028 | 0.020 |
|  | 75+ | 0.071 | 0.049 | 0.015 | -0.010 | -0.008 |
|  | White | 0.034 | 0.025 | 0.019 | 0.012 | 0.006 |
|  | Asian | 0.000 | 0.014 | 0.009 | 0.000 | 0.002 |
|  | Black | -0.016 | -0.018 | -0.012 | -0.008 | -0.003 |
|  | Hispanic | 0.002 | -0.021 | 0.000 | 0.001 | -0.005 |
|  | Native | -0.020 | 0.000 | -0.016 | -0.005 | 0.000 |
| Cardiomegaly | Female | -0.031 | -0.013 | 0.018 | -0.006 | -0.015 |
|  | Male | 0.031 | 0.013 | -0.018 | 0.006 | 0.015 |
|  | 0-25 | -0.113 | -0.123 | -0.031 | -0.026 | -0.015 |
|  | 26-50 | 0.111 | -0.042 | 0.047 | -0.033 | -0.003 |
|  | 51-75 | -0.039 | 0.075 | 0.001 | 0.046 | 0.013 |
|  | 75+ | 0.042 | 0.090 | -0.018 | 0.013 | 0.004 |
|  | White | 0.045 | -0.048 | 0.050 | 0.022 | 0.005 |
|  | Asian | 0.000 | 0.000 | 0.008 | -0.004 | 0.003 |
|  | Black | 0.034 | -0.033 | -0.047 | -0.019 | -0.001 |
|  | Hispanic | -0.071 | 0.067 | -0.012 | 0.000 | 0.000 |
|  | Native | -0.007 | 0.013 | 0.000 | 0.001 | -0.007 |
| Consolidation | Female | -0.048 | -0.058 | -0.023 | -0.074 | -0.013 |
|  | Male | 0.048 | 0.058 | 0.023 | 0.074 | 0.013 |
|  | 0-25 | -0.054 | -0.062 | -0.030 | -0.073 | -0.018 |
|  | 26-50 | 0.011 | 0.019 | 0.017 | -0.033 | -0.004 |
|  | 51-75 | -0.034 | -0.001 | -0.014 | 0.067 | 0.015 |
|  | 75+ | 0.077 | 0.044 | 0.027 | 0.038 | 0.007 |
|  | White | 0.097 | 0.081 | 0.031 | 0.032 | 0.014 |
|  | Asian | 0.021 | 0.000 | 0.000 | -0.049 | 0.000 |
|  | Black | -0.094 | -0.083 | -0.032 | 0.030 | -0.015 |
|  | Hispanic | -0.023 | 0.017 | 0.021 | -0.014 | 0.002 |
|  | Native | 0.000 | -0.015 | -0.020 | 0.000 | -0.001 |
| Edema | Female | -0.068 | -0.034 | 0.024 | -0.005 | -0.014 |
|  | Male | 0.068 | 0.034 | -0.024 | 0.005 | 0.014 |
|  | 0-25 | -0.084 | -0.115 | -0.027 | -0.005 | -0.016 |
|  | 26-50 | 0.104 | -0.027 | -0.022 | -0.008 | 0.003 |
|  | 51-75 | -0.077 | 0.185 | 0.029 | 0.011 | 0.008 |
|  | 75+ | 0.057 | -0.043 | 0.020 | 0.002 | 0.004 |
|  | White | 0.068 | 0.024 | 0.056 | 0.000 | 0.003 |
|  | Asian | 0.019 | 0.006 | 0.000 | -0.040 | 0.001 |
|  | Black | -0.007 | -0.014 | -0.049 | -0.044 | 0.000 |
|  | Hispanic | -0.080 | -0.015 | -0.012 | 0.049 | 0.001 |
|  | Native | 0.000 | 0.000 | 0.005 | 0.035 | -0.005 |
| Enlarged Cardio Mediastinum | Female | -0.061 | -0.037 | -0.028 | -0.012 | -0.012 |
|  | Male | 0.061 | 0.037 | 0.028 | 0.012 | 0.012 |
|  | 0-25 | -0.045 | -0.066 | -0.025 | -0.011 | -0.018 |
|  | 26-50 | 0.035 | 0.012 | 0.052 | -0.080 | -0.005 |
|  | 51-75 | -0.057 | 0.085 | -0.034 | 0.066 | 0.014 |
|  | 75+ | 0.066 | -0.031 | 0.007 | 0.025 | 0.008 |
|  | White | 0.021 | 0.009 | 0.031 | 0.045 | -0.003 |
|  | Asian | 0.046 | 0.000 | 0.001 | -0.041 | 0.005 |
|  | Black | 0.000 | -0.023 | -0.014 | -0.037 | 0.000 |
|  | Hispanic | -0.048 | 0.029 | 0.000 | 0.033 | -0.003 |
|  | Native | -0.019 | -0.014 | -0.017 | 0.000 | 0.001 |
| Fracture | Female | -0.053 | -0.060 | 0.015 | -0.049 | -0.014 |
|  | Male | 0.053 | 0.060 | -0.015 | 0.049 | 0.014 |
|  | 0-25 | -0.067 | -0.068 | -0.023 | -0.047 | -0.015 |
|  | 26-50 | 0.132 | -0.024 | -0.052 | -0.045 | -0.004 |
|  | 51-75 | -0.139 | 0.053 | 0.058 | 0.058 | 0.014 |
|  | 75+ | 0.073 | 0.038 | 0.016 | 0.034 | 0.004 |
|  | White | 0.042 | 0.034 | 0.057 | 0.000 | -0.002 |
|  | Asian | 0.000 | -0.007 | 0.002 | 0.004 | -0.007 |
|  | Black | -0.026 | 0.000 | -0.046 | -0.040 | 0.000 |
|  | Hispanic | -0.051 | 0.033 | -0.013 | 0.050 | 0.007 |
|  | Native | 0.035 | -0.060 | 0.000 | -0.014 | 0.002 |
| Lung Lesion | Female | -0.062 | -0.065 | -0.022 | -0.017 | -0.011 |
|  | Male | 0.062 | 0.065 | 0.022 | 0.017 | 0.011 |
|  | 0-25 | -0.032 | -0.078 | -0.019 | 0.016 | -0.017 |
|  | 26-50 | 0.056 | -0.014 | -0.002 | -0.012 | 0.004 |
|  | 51-75 | -0.114 | 0.069 | 0.006 | 0.009 | 0.019 |
|  | 75+ | 0.090 | 0.023 | 0.015 | -0.013 | -0.007 |
|  | White | 0.000 | 0.006 | 0.040 | 0.014 | 0.005 |
|  | Asian | 0.040 | -0.014 | -0.005 | 0.000 | 0.001 |
|  | Black | 0.006 | -0.022 | 0.000 | -0.029 | 0.000 |
|  | Hispanic | -0.029 | 0.030 | -0.055 | 0.028 | -0.004 |
|  | Native | -0.017 | 0.000 | 0.020 | -0.013 | -0.002 |
| Lung Opacity | Female | -0.080 | -0.032 | 0.017 | -0.004 | -0.014 |
|  | Male | 0.080 | 0.032 | -0.017 | 0.004 | 0.014 |
|  | 0-25 | -0.042 | -0.094 | -0.016 | -0.043 | -0.015 |
|  | 26-50 | 0.109 | -0.025 | 0.000 | 0.012 | 0.002 |
|  | 51-75 | -0.168 | 0.043 | 0.031 | -0.009 | 0.017 |
|  | 75+ | 0.101 | 0.076 | -0.015 | 0.040 | -0.004 |
|  | White | 0.068 | 0.047 | 0.051 | -0.042 | -0.005 |
|  | Asian | -0.031 | 0.000 | 0.000 | 0.050 | -0.003 |
|  | Black | -0.052 | -0.003 | -0.037 | -0.035 | 0.000 |
|  | Hispanic | 0.000 | 0.001 | 0.004 | 0.027 | 0.007 |
|  | Native | 0.015 | -0.045 | -0.018 | 0.000 | 0.002 |
| Pleural Effusion | Female | -0.068 | -0.036 | 0.013 | -0.021 | -0.012 |
|  | Male | 0.068 | 0.036 | -0.013 | 0.021 | 0.012 |
|  | 0-25 | -0.029 | -0.082 | -0.085 | 0.020 | -0.016 |
|  | 26-50 | 0.027 | 0.009 | 0.006 | -0.017 | 0.003 |
|  | 51-75 | -0.086 | 0.090 | 0.085 | 0.013 | 0.020 |
|  | 75+ | 0.089 | -0.017 | -0.006 | -0.017 | -0.008 |
|  | White | 0.073 | 0.000 | 0.040 | 0.042 | 0.006 |
|  | Asian | -0.019 | -0.042 | -0.041 | 0.015 | -0.008 |
|  | Black | -0.055 | -0.025 | -0.033 | -0.012 | 0.000 |
|  | Hispanic | 0.000 | 0.056 | 0.020 | 0.000 | 0.004 |
|  | Native | 0.001 | 0.010 | 0.014 | -0.045 | -0.001 |
| Pleural Other | Female | -0.068 | -0.039 | 0.025 | -0.012 | -0.002 |
|  | Male | 0.068 | 0.039 | -0.025 | 0.012 | 0.002 |
|  | 0-25 | -0.059 | -0.093 | -0.025 | -0.038 | -0.017 |
|  | 26-50 | 0.099 | 0.019 | 0.010 | -0.035 | -0.001 |
|  | 51-75 | -0.153 | 0.093 | 0.034 | 0.038 | 0.014 |
|  | 75+ | 0.113 | -0.020 | -0.019 | 0.036 | 0.004 |
|  | White | 0.068 | 0.056 | 0.053 | 0.062 | 0.017 |
|  | Asian | -0.017 | -0.041 | 0.000 | 0.000 | -0.002 |
|  | Black | -0.060 | 0.000 | -0.044 | 0.011 | 0.000 |
|  | Hispanic | 0.010 | 0.018 | 0.013 | -0.044 | -0.018 |
|  | Native | 0.000 | -0.033 | -0.023 | -0.029 | 0.002 |
| Pneumonia | Female | -0.078 | -0.069 | -0.044 | -0.006 | -0.012 |
|  | Male | 0.078 | 0.069 | 0.044 | 0.006 | 0.012 |
|  | 0-25 | -0.049 | -0.042 | -0.031 | -0.024 | -0.014 |
|  | 26-50 | 0.035 | 0.036 | 0.001 | -0.022 | -0.001 |
|  | 51-75 | -0.077 | 0.013 | -0.038 | 0.025 | 0.009 |
|  | 75+ | 0.092 | -0.007 | 0.068 | 0.021 | 0.006 |
|  | White | 0.016 | 0.034 | 0.039 | 0.000 | 0.014 |
|  | Asian | 0.083 | -0.051 | -0.011 | -0.030 | -0.016 |
|  | Black | -0.047 | 0.000 | -0.032 | -0.017 | 0.008 |
|  | Hispanic | -0.052 | 0.034 | 0.000 | 0.034 | 0.000 |
|  | Native | 0.000 | -0.017 | 0.004 | 0.013 | -0.005 |
| Pneumothorax | Female | -0.059 | -0.029 | 0.035 | -0.013 | -0.011 |
|  | Male | 0.059 | 0.029 | -0.035 | 0.013 | 0.011 |
|  | 0-25 | -0.073 | -0.082 | -0.038 | 0.029 | -0.016 |
|  | 26-50 | 0.054 | 0.010 | 0.003 | 0.026 | 0.028 |
|  | 51-75 | -0.090 | 0.079 | -0.021 | -0.033 | -0.019 |
|  | 75+ | 0.109 | -0.006 | 0.056 | -0.022 | 0.007 |
|  | White | 0.082 | 0.068 | 0.050 | 0.067 | 0.019 |
|  | Asian | 0.000 | -0.042 | 0.000 | -0.046 | -0.001 |
|  | Black | 0.011 | -0.030 | -0.031 | -0.052 | 0.000 |
|  | Hispanic | -0.086 | 0.005 | -0.020 | 0.031 | -0.019 |
|  | Native | -0.007 | 0.000 | 0.002 | 0.000 | 0.001 |
| Support Devices | Female | -0.064 | -0.020 | -0.034 | 0.019 | -0.014 |
|  | Male | 0.064 | 0.020 | 0.034 | -0.019 | 0.014 |
|  | 0-25 | -0.069 | -0.086 | -0.034 | -0.033 | -0.017 |
|  | 26-50 | 0.078 | -0.007 | 0.003 | 0.031 | -0.002 |
|  | 51-75 | -0.102 | 0.062 | 0.076 | 0.033 | 0.014 |
|  | 75+ | 0.094 | 0.031 | -0.044 | -0.031 | 0.005 |
|  | White | 0.096 | 0.081 | 0.047 | 0.032 | 0.021 |
|  | Asian | 0.021 | 0.000 | 0.008 | 0.000 | -0.001 |
|  | Black | -0.036 | -0.042 | -0.039 | -0.026 | 0.000 |
|  | Hispanic | 0.000 | 0.011 | 0.000 | 0.021 | 0.001 |
|  | Native | -0.081 | -0.050 | -0.016 | -0.027 | -0.021 |

Table 2. Overview of true positive rate (TPR) disparities for NPT classifiers across subgroups differentiated by sex and age for the Chest x-ray 14 dataset. The table presents the TPR disparities for each subgroup across five IC levels (IC=1, IC=3, IC=7, IC=15, IC=31). Disparities are calculated by comparing subgroup performance within each IC level, where a positive TPR disparity indicates better performance for that group and a negative TPR disparity indicates worse performance.

|  |  | **IC=1** | **IC=3** | **IC=7** | **IC=15** | **IC=31** |
| --- | --- | --- | --- | --- | --- | --- |
| **Pathology** | **Subgroup** | **TPR Disparity** | **TPR Disparity** | **TPR Disparity** | **TPR Disparity** | **TPR Disparity** |
| Atelectasis | Female | -0.053 | -0.020 | -0.010 | 0.040 | -0.005 |
|  | Male | 0.053 | 0.020 | 0.010 | -0.040 | 0.005 |
|  | 0-25 | -0.049 | -0.080 | -0.015 | -0.027 | -0.017 |
|  | 26-50 | 0.074 | -0.016 | 0.010 | 0.021 | 0.010 |
|  | 51-75 | -0.145 | 0.026 | 0.058 | 0.051 | -0.001 |
|  | 75+ | 0.121 | 0.070 | -0.053 | -0.044 | 0.007 |
| Cardiomegaly | Female | -0.009 | -0.003 | 0.034 | -0.003 | -0.005 |
|  | Male | 0.009 | 0.003 | -0.034 | 0.003 | 0.005 |
|  | 0-25 | -0.107 | -0.127 | -0.026 | -0.016 | -0.014 |
|  | 26-50 | 0.109 | -0.040 | 0.044 | -0.023 | -0.005 |
|  | 51-75 | -0.049 | 0.080 | 0.012 | 0.031 | 0.012 |
|  | 75+ | 0.047 | 0.088 | -0.031 | 0.008 | 0.007 |
| Consolidation | Female | -0.069 | -0.010 | 0.015 | -0.006 | 0.010 |
|  | Male | 0.069 | 0.010 | -0.015 | 0.006 | -0.010 |
|  | 0-25 | -0.069 | -0.107 | -0.015 | -0.002 | -0.004 |
|  | 26-50 | 0.109 | 0.024 | -0.023 | -0.010 | 0.005 |
|  | 51-75 | -0.097 | 0.169 | 0.017 | 0.008 | -0.010 |
|  | 75+ | 0.057 | -0.086 | 0.020 | 0.004 | 0.009 |
| Edema | Female | -0.059 | -0.048 | -0.002 | -0.002 | -0.006 |
|  | Male | 0.059 | 0.048 | 0.002 | 0.002 | 0.006 |
|  | 0-25 | -0.032 | -0.081 | -0.016 | 0.020 | -0.006 |
|  | 26-50 | 0.055 | -0.018 | 0.002 | 0.003 | 0.013 |
|  | 51-75 | -0.123 | 0.069 | -0.001 | -0.012 | 0.006 |
|  | 75+ | 0.100 | 0.030 | 0.016 | -0.011 | -0.013 |
| Effusion | Female | -0.055 | -0.045 | 0.046 | -0.014 | 0.017 |
|  | Male | 0.055 | 0.045 | -0.046 | 0.014 | -0.017 |
|  | 0-25 | -0.044 | -0.089 | -0.016 | -0.034 | -0.019 |
|  | 26-50 | 0.097 | 0.024 | 0.024 | -0.037 | -0.009 |
|  | 51-75 | -0.177 | 0.083 | 0.004 | 0.027 | 0.004 |
|  | 75+ | 0.125 | -0.018 | -0.011 | 0.044 | 0.024 |
| Emphysema | Female | -0.056 | -0.049 | -0.015 | -0.002 | 0.002 |
|  | Male | 0.056 | 0.049 | 0.015 | 0.002 | -0.002 |
|  | 0-25 | -0.071 | -0.057 | -0.028 | -0.024 | -0.019 |
|  | 26-50 | 0.041 | 0.033 | -0.039 | 0.016 | -0.007 |
|  | 51-75 | -0.046 | -0.025 | 0.049 | 0.014 | 0.006 |
|  | 75+ | 0.076 | 0.049 | 0.018 | -0.006 | 0.020 |
| Fibrosis | Female | -0.053 | -0.012 | -0.015 | 0.020 | 0.002 |
|  | Male | 0.053 | 0.012 | 0.015 | -0.020 | -0.002 |
|  | 0-25 | -0.069 | -0.081 | -0.028 | -0.022 | -0.003 |
|  | 26-50 | 0.079 | -0.006 | 0.010 | 0.031 | 0.006 |
|  | 51-75 | -0.117 | 0.055 | 0.051 | 0.023 | -0.012 |
|  | 75+ | 0.106 | 0.032 | -0.033 | -0.031 | 0.009 |
| Hernia | Female | -0.031 | -0.073 | 0.019 | -0.039 | -0.029 |
|  | Male | 0.031 | 0.073 | -0.019 | 0.039 | 0.029 |
|  | 0-25 | -0.069 | -0.058 | -0.011 | -0.035 | -0.018 |
|  | 26-50 | 0.015 | -0.029 | -0.045 | -0.046 | -0.008 |
|  | 51-75 | 0.066 | 0.037 | 0.036 | 0.051 | 0.007 |
|  | 75+ | -0.012 | 0.050 | 0.021 | 0.031 | 0.019 |
| Infiltration | Female | -0.062 | -0.055 | -0.026 | -0.008 | -0.004 |
|  | Male | 0.062 | 0.055 | 0.026 | 0.008 | 0.004 |
|  | 0-25 | -0.049 | -0.035 | -0.025 | -0.020 | -0.008 |
|  | 26-50 | 0.041 | 0.048 | 0.004 | -0.019 | 0.000 |
|  | 51-75 | -0.080 | -0.015 | -0.048 | 0.017 | -0.008 |
|  | 75+ | 0.087 | 0.001 | 0.068 | 0.022 | 0.016 |
| Mass | Female | -0.074 | -0.014 | -0.034 | -0.013 | 0.003 |
|  | Male | 0.074 | 0.014 | 0.034 | 0.013 | -0.003 |
|  | 0-25 | -0.037 | -0.053 | -0.014 | -0.008 | -0.020 |
|  | 26-50 | 0.033 | 0.020 | 0.050 | -0.084 | 0.007 |
|  | 51-75 | -0.061 | 0.056 | -0.052 | 0.061 | -0.003 |
|  | 75+ | 0.065 | -0.023 | 0.016 | 0.031 | 0.016 |
| Nodule | Female | -0.042 | -0.070 | -0.021 | -0.081 | 0.004 |
|  | Male | 0.042 | 0.070 | 0.021 | 0.081 | -0.004 |
|  | 0-25 | -0.043 | -0.048 | -0.034 | -0.068 | -0.011 |
|  | 26-50 | 0.017 | 0.017 | 0.027 | -0.018 | -0.009 |
|  | 51-75 | -0.061 | -0.025 | -0.023 | 0.034 | 0.004 |
|  | 75+ | 0.088 | 0.056 | 0.030 | 0.052 | 0.016 |
| Pleural Thickening | Female | -0.068 | -0.046 | -0.002 | -0.003 | 0.010 |
|  | Male | 0.068 | 0.046 | 0.002 | 0.003 | -0.010 |
|  | 0-25 | -0.029 | -0.080 | -0.087 | -0.032 | -0.014 |
|  | 26-50 | 0.024 | 0.022 | 0.016 | 0.018 | 0.011 |
|  | 51-75 | -0.093 | 0.070 | 0.079 | -0.002 | 0.006 |
|  | 75+ | 0.098 | -0.012 | -0.008 | 0.017 | -0.003 |
| Pneumonia | Female | -0.058 | -0.041 | 0.035 | 0.013 | -0.003 |
|  | Male | 0.058 | 0.041 | -0.035 | -0.013 | 0.003 |
|  | 0-25 | -0.032 | -0.097 | -0.005 | -0.030 | -0.015 |
|  | 26-50 | 0.114 | -0.024 | 0.010 | 0.018 | 0.002 |
|  | 51-75 | -0.195 | 0.031 | 0.000 | -0.040 | 0.020 |
|  | 75+ | 0.113 | 0.090 | -0.005 | 0.053 | -0.007 |
| Pneumothorax | Female | -0.067 | -0.014 | 0.029 | -0.013 | -0.023 |
|  | Male | 0.067 | 0.014 | -0.029 | 0.013 | 0.023 |
|  | 0-25 | -0.066 | -0.084 | -0.032 | 0.042 | -0.016 |
|  | 26-50 | 0.065 | 0.020 | 0.007 | 0.034 | 0.038 |
|  | 51-75 | -0.105 | -0.062 | -0.027 | -0.062 | -0.031 |
|  | 75+ | 0.105 | 0.125 | 0.051 | -0.015 | 0.009 |

Table 3. Overview of true positive rate (TPR) disparities for NPT classifiers across subgroups differentiated by sex, age, and race for the MIMIC-CXR dataset. The table presents the TPR disparities for each subgroup across five IC levels (IC=1, IC=3, IC=7, IC=15, IC=31). Disparities are calculated by comparing subgroup performance within each IC level, where a positive TPR disparity indicates better performance for that group and a negative TPR disparity indicates worse performance.

|  |  | **IC=1** | **IC=3** | **IC=7** | **IC=15** | **IC=31** |
| --- | --- | --- | --- | --- | --- | --- |
| **Pathology** | **Subgroup** | **TPR Disparity** | **TPR Disparity** | **TPR Disparity** | **TPR Disparity** | **TPR Disparity** |
| Airspace Opacity | Female | -0.098 | -0.051 | -0.079 | 0.044 | -0.018 |
|  | Male | 0.098 | 0.051 | 0.079 | -0.044 | 0.018 |
|  | 0-25 | -0.086 | -0.126 | -0.065 | -0.074 | -0.051 |
|  | 26-50 | 0.079 | 0.020 | 0.040 | 0.061 | 0.038 |
|  | 51-75 | -0.047 | 0.112 | 0.067 | 0.053 | 0.033 |
|  | 75+ | 0.053 | -0.006 | -0.043 | -0.040 | -0.020 |
|  | White | 0.081 | 0.079 | 0.018 | 0.042 | 0.034 |
|  | Asian | 0.000 | 0.047 | -0.042 | -0.042 | 0.000 |
|  | Black | -0.034 | 0.000 | -0.040 | 0.000 | -0.033 |
|  | Hispanic | 0.016 | -0.083 | 0.064 | 0.032 | 0.035 |
|  | Native | -0.063 | -0.043 | 0.000 | -0.032 | -0.036 |
| Atelectasis | Female | -0.082 | -0.058 | -0.045 | -0.017 | 0.011 |
|  | Male | 0.082 | 0.058 | 0.045 | 0.017 | -0.011 |
|  | 0-25 | -0.061 | -0.055 | -0.041 | -0.028 | -0.021 |
|  | 26-50 | 0.022 | 0.066 | -0.046 | -0.010 | -0.015 |
|  | 51-75 | 0.073 | -0.075 | 0.053 | 0.002 | 0.015 |
|  | 75+ | -0.034 | 0.064 | 0.034 | 0.036 | 0.021 |
|  | White | 0.000 | 0.003 | 0.036 | 0.020 | -0.013 |
|  | Asian | -0.040 | 0.017 | 0.000 | -0.001 | -0.013 |
|  | Black | -0.036 | -0.049 | -0.014 | -0.037 | 0.017 |
|  | Hispanic | 0.012 | 0.000 | 0.006 | 0.019 | 0.014 |
|  | Native | 0.064 | -0.021 | -0.028 | 0.000 | -0.005 |
| Cardiomegaly | Female | -0.051 | -0.082 | 0.013 | -0.009 | 0.022 |
|  | Male | 0.051 | 0.082 | -0.013 | 0.009 | -0.022 |
|  | 0-25 | -0.051 | 0.020 | 0.001 | -0.035 | -0.036 |
|  | 26-50 | 0.063 | -0.092 | -0.037 | 0.036 | -0.013 |
|  | 51-75 | -0.050 | 0.083 | -0.008 | -0.013 | 0.022 |
|  | 75+ | 0.038 | -0.011 | 0.044 | 0.012 | 0.027 |
|  | White | 0.069 | 0.056 | 0.043 | 0.011 | 0.000 |
|  | Asian | 0.000 | -0.006 | -0.021 | -0.018 | -0.029 |
|  | Black | -0.038 | 0.000 | 0.000 | -0.022 | 0.026 |
|  | Hispanic | -0.055 | -0.070 | 0.019 | 0.000 | 0.034 |
|  | Native | 0.024 | 0.020 | -0.041 | 0.029 | -0.031 |
| Consolidation | Female | -0.060 | -0.047 | -0.022 | 0.027 | -0.012 |
|  | Male | 0.060 | 0.047 | 0.022 | -0.027 | 0.012 |
|  | 0-25 | -0.011 | -0.089 | -0.029 | -0.021 | -0.035 |
|  | 26-50 | 0.021 | 0.074 | 0.031 | 0.030 | 0.014 |
|  | 51-75 | -0.087 | 0.029 | 0.054 | 0.014 | 0.028 |
|  | 75+ | 0.077 | -0.014 | -0.055 | -0.023 | -0.008 |
|  | White | 0.079 | 0.000 | 0.042 | 0.000 | 0.018 |
|  | Asian | -0.003 | -0.039 | -0.048 | -0.027 | 0.030 |
|  | Black | 0.000 | -0.073 | 0.028 | -0.011 | -0.020 |
|  | Hispanic | -0.095 | 0.082 | 0.000 | 0.022 | -0.028 |
|  | Native | 0.020 | 0.030 | -0.022 | 0.016 | 0.000 |
| Edema | Female | -0.100 | -0.077 | 0.077 | -0.030 | -0.013 |
|  | Male | 0.100 | 0.077 | -0.077 | 0.030 | 0.013 |
|  | 0-25 | -0.120 | -0.053 | -0.017 | -0.051 | 0.005 |
|  | 26-50 | 0.092 | 0.051 | 0.000 | 0.045 | -0.019 |
|  | 51-75 | -0.040 | -0.021 | -0.035 | 0.034 | -0.014 |
|  | 75+ | 0.068 | 0.023 | 0.052 | -0.028 | 0.028 |
|  | White | 0.105 | 0.072 | 0.068 | 0.052 | 0.022 |
|  | Asian | 0.028 | -0.023 | 0.000 | 0.020 | 0.000 |
|  | Black | 0.000 | -0.061 | -0.023 | 0.000 | -0.005 |
|  | Hispanic | -0.085 | 0.013 | -0.051 | -0.037 | 0.028 |
|  | Native | -0.047 | 0.000 | 0.007 | -0.035 | -0.044 |
| Enlarged Cardio Mediastinum | Female | -0.023 | -0.023 | -0.024 | 0.015 | -0.018 |
|  | Male | 0.023 | 0.023 | 0.024 | -0.015 | 0.018 |
|  | 0-25 | -0.113 | -0.114 | -0.024 | -0.018 | 0.014 |
|  | 26-50 | 0.108 | -0.028 | 0.002 | -0.069 | -0.017 |
|  | 51-75 | -0.020 | 0.062 | 0.049 | 0.045 | -0.031 |
|  | 75+ | 0.025 | 0.080 | -0.027 | 0.043 | 0.034 |
|  | White | 0.067 | 0.061 | 0.049 | 0.016 | 0.002 |
|  | Asian | -0.033 | 0.000 | -0.030 | 0.047 | -0.020 |
|  | Black | 0.077 | -0.057 | -0.020 | 0.000 | 0.022 |
|  | Hispanic | -0.111 | -0.027 | 0.000 | -0.031 | -0.004 |
|  | Native | 0.000 | 0.023 | 0.001 | -0.032 | 0.000 |
| Fracture | Female | -0.076 | -0.044 | -0.030 | -0.029 | -0.003 |
|  | Male | 0.076 | 0.044 | 0.030 | 0.029 | 0.003 |
|  | 0-25 | -0.010 | -0.105 | -0.027 | -0.058 | -0.003 |
|  | 26-50 | 0.130 | -0.023 | -0.047 | -0.039 | 0.029 |
|  | 51-75 | -0.262 | 0.094 | 0.049 | 0.070 | -0.060 |
|  | 75+ | 0.142 | 0.033 | 0.025 | 0.027 | 0.034 |
|  | White | 0.091 | 0.000 | 0.033 | -0.043 | 0.019 |
|  | Asian | 0.047 | 0.031 | -0.044 | 0.000 | 0.000 |
|  | Black | 0.000 | -0.070 | -0.003 | -0.029 | -0.014 |
|  | Hispanic | -0.097 | 0.086 | 0.000 | 0.052 | 0.001 |
|  | Native | -0.041 | -0.047 | 0.014 | 0.020 | -0.005 |
| Lung Lesion | Female | -0.088 | -0.052 | 0.042 | 0.031 | 0.023 |
|  | Male | 0.088 | 0.052 | -0.042 | -0.031 | -0.023 |
|  | 0-25 | -0.009 | -0.114 | -0.040 | 0.011 | -0.003 |
|  | 26-50 | 0.125 | 0.026 | 0.003 | -0.007 | 0.005 |
|  | 51-75 | -0.208 | 0.095 | -0.014 | -0.053 | 0.023 |
|  | 75+ | 0.092 | -0.007 | 0.051 | 0.050 | -0.025 |
|  | White | 0.084 | 0.078 | 0.052 | 0.035 | 0.019 |
|  | Asian | -0.046 | 0.000 | -0.016 | 0.000 | 0.006 |
|  | Black | 0.000 | 0.023 | -0.062 | -0.006 | 0.000 |
|  | Hispanic | -0.049 | -0.043 | 0.026 | -0.031 | -0.024 |
|  | Native | 0.012 | -0.058 | 0.000 | 0.002 | -0.001 |
| Pleural Effusion | Female | -0.004 | -0.054 | 0.065 | -0.015 | -0.014 |
|  | Male | 0.004 | 0.054 | -0.065 | 0.015 | 0.014 |
|  | 0-25 | -0.056 | -0.098 | -0.068 | 0.061 | 0.023 |
|  | 26-50 | 0.084 | -0.030 | -0.055 | -0.064 | -0.002 |
|  | 51-75 | -0.080 | 0.046 | 0.060 | -0.017 | 0.016 |
|  | 75+ | 0.052 | 0.082 | 0.063 | 0.020 | -0.037 |
|  | White | 0.088 | 0.040 | 0.050 | 0.032 | -0.006 |
|  | Asian | -0.063 | -0.023 | 0.018 | 0.008 | 0.000 |
|  | Black | 0.000 | 0.000 | -0.036 | -0.035 | 0.024 |
|  | Hispanic | -0.085 | 0.059 | -0.032 | 0.000 | -0.025 |
|  | Native | 0.060 | -0.077 | 0.000 | -0.005 | 0.007 |
| Pleural Other | Female | -0.080 | -0.072 | -0.044 | -0.037 | -0.021 |
|  | Male | 0.080 | 0.072 | 0.044 | 0.037 | 0.021 |
|  | 0-25 | -0.103 | -0.104 | -0.106 | -0.001 | -0.034 |
|  | 26-50 | 0.050 | -0.010 | 0.012 | -0.019 | 0.006 |
|  | 51-75 | -0.054 | 0.022 | 0.103 | 0.041 | -0.004 |
|  | 75+ | 0.107 | 0.092 | -0.008 | -0.021 | 0.031 |
|  | White | 0.145 | 0.068 | 0.047 | 0.023 | -0.004 |
|  | Asian | 0.000 | 0.030 | 0.046 | -0.044 | -0.035 |
|  | Black | -0.064 | -0.088 | -0.077 | 0.029 | 0.000 |
|  | Hispanic | 0.039 | 0.000 | 0.000 | -0.008 | 0.007 |
|  | Native | -0.119 | -0.009 | -0.016 | 0.000 | 0.032 |
| Pneumonia | Female | -0.069 | -0.040 | 0.014 | -0.011 | -0.020 |
|  | Male | 0.069 | 0.040 | -0.014 | 0.011 | 0.020 |
|  | 0-25 | -0.008 | -0.123 | -0.052 | 0.015 | 0.030 |
|  | 26-50 | 0.056 | 0.023 | 0.023 | -0.060 | -0.002 |
|  | 51-75 | -0.168 | 0.133 | 0.060 | 0.083 | 0.013 |
|  | 75+ | 0.120 | -0.033 | -0.031 | -0.038 | -0.041 |
|  | White | 0.122 | 0.014 | 0.047 | 0.017 | 0.015 |
|  | Asian | 0.000 | -0.069 | -0.073 | -0.007 | 0.000 |
|  | Black | 0.023 | 0.060 | 0.000 | -0.055 | 0.017 |
|  | Hispanic | -0.099 | 0.000 | 0.058 | 0.046 | -0.027 |
|  | Native | -0.046 | -0.005 | -0.032 | 0.000 | -0.005 |
| Pneumothorax | Female | -0.078 | -0.097 | -0.030 | 0.015 | 0.001 |
|  | Male | 0.078 | 0.097 | 0.030 | -0.015 | -0.001 |
|  | 0-25 | -0.071 | -0.033 | -0.069 | -0.073 | 0.000 |
|  | 26-50 | 0.047 | 0.060 | -0.014 | 0.035 | -0.013 |
|  | 51-75 | -0.035 | -0.059 | 0.076 | -0.003 | 0.027 |
|  | 75+ | 0.059 | 0.032 | 0.007 | 0.041 | -0.014 |
|  | White | 0.037 | 0.050 | 0.019 | 0.055 | 0.022 |
|  | Asian | 0.000 | -0.073 | 0.000 | -0.030 | 0.012 |
|  | Black | 0.058 | -0.031 | -0.040 | 0.019 | 0.000 |
|  | Hispanic | -0.082 | 0.000 | 0.068 | -0.044 | -0.021 |
|  | Native | -0.013 | 0.054 | -0.048 | 0.000 | -0.013 |
| Support Devices | Female | -0.054 | -0.055 | 0.007 | 0.019 | -0.006 |
|  | Male | 0.054 | 0.055 | -0.007 | -0.019 | 0.006 |
|  | 0-25 | -0.049 | -0.011 | 0.018 | -0.023 | -0.016 |
|  | 26-50 | 0.081 | 0.004 | -0.062 | 0.014 | -0.031 |
|  | 51-75 | -0.072 | 0.030 | -0.003 | -0.026 | 0.022 |
|  | 75+ | 0.041 | -0.022 | 0.047 | 0.035 | 0.025 |
|  | White | 0.093 | 0.052 | 0.031 | 0.048 | 0.034 |
|  | Asian | 0.058 | 0.038 | 0.000 | -0.044 | -0.038 |
|  | Black | -0.047 | 0.000 | -0.013 | -0.034 | 0.009 |
|  | Hispanic | -0.103 | -0.079 | -0.057 | 0.030 | 0.000 |
|  | Native | 0.000 | -0.011 | 0.039 | 0.000 | -0.005 |
