# Supplementary material for: Intersection of Performance, Interpretability, and Fairness in Neural Prototype Tree for Chest X-Ray Pathology Detection: Algorithm Development and Validation Study
Source: JMIR Form Res. 2024 Dec 5;8:e59045. doi: 10.2196/59045 (PMC11659703; doi:10.2196/59045)
Supplement: Multimedia Appendix 5 [file formative_v8i1e59045_app5.docx]

## Multimedia Appendix-5: Linear Regression for Investigating the Impact of IC Level on Mean TPR Disparity

Table 1. Linear regression results analyzing the effect of IC level on mean TPR disparity across demographic attributes, specifically sex and age in the Chest X-ray 14 dataset, and sex, age, and race in the CheXpert and MIMIC-CXR dataset. The table reports the coefficient for IC level, standard error, t-statistic, adjusted R-squared, F-statistic, sample size, and p-value. The corrected p-values were calculated using the Benjamini-Hochberg procedure to control for the false discovery rate.

| **Dataset** | **Demographic**  **Attributes** | **Coefficient (IC Level)** | **Std. Error** | **t-statistic** | **Adjusted R-squared** | **F-statistic** | **Sample Size** | **p-value** | | **corrected p-value** |
| --- | --- | --- | --- | --- | --- | --- | --- | --- | --- | --- |
| CheXpert | Sex | -1.44E-03 | 1.96E-04 | -7.37 | 0.69 | 54.33 | 25 | | < .001 | < .001 |
|  | Age | -2.36E-03 | 2.34E-04 | -10.12 | 0.81 | 102.5 | 25 | | < .001 | < .001 |
|  | Race | -1.61E-03 | 1.10E-04 | -14.57 | 0.90 | 212.2 | 25 | | < .001 | < .001 |
| MIMIC-CXR | Sex | -1.69E-03 | 1.68E-04 | -10.01 | 0.80 | 100.2 | 25 | | < .001 | < .001 |
|  | Age | -2.01E-03 | 1.54E-04 | -13.02 | 0.88 | 168.9 | 25 | | < .001 | < .001 |
|  | Race | -1.97E-03 | 2.07E-04 | -9.64 | 0.79 | 92.99 | 25 | | < .001 | < .001 |
| Chest X-ray 14 | Sex | -1.06E-03 | 1.98E-04 | -5.38 | 0.54 | 28.92 | 25 | | < .001 | < .001 |
|  | Age | -2.23E-03 | 3.82e-04 | -5.85 | 0.58 | 34.22 | 25 | | < .001 | < .001 |
