# Supplementary material for: Intersection of Performance, Interpretability, and Fairness in Neural Prototype Tree for Chest X-Ray Pathology Detection: Algorithm Development and Validation Study
Source: JMIR Form Res. 2024 Dec 5;8:e59045. doi: 10.2196/59045 (PMC11659703; doi:10.2196/59045)
Supplement: Multimedia Appendix 6 [file formative_v8i1e59045_app6.docx]

## Multimedia Appendix-6: Global Explanations of NPT Classifiers


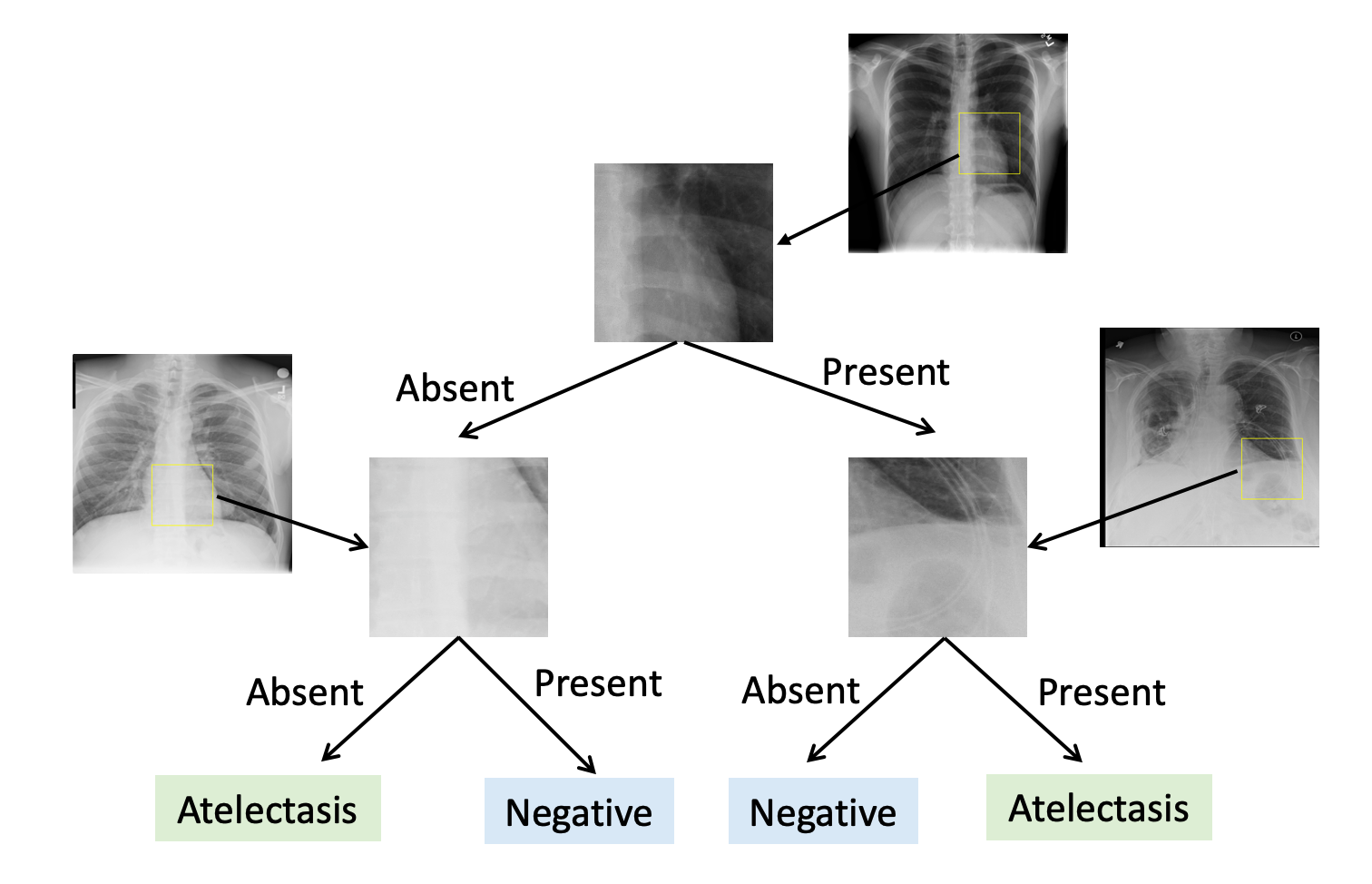


Figure 1. Global explanation of the NPT classifier’s decision-making process for detecting atelectasis (IC=3) with AP view CXRs in Chest X-ray 14.


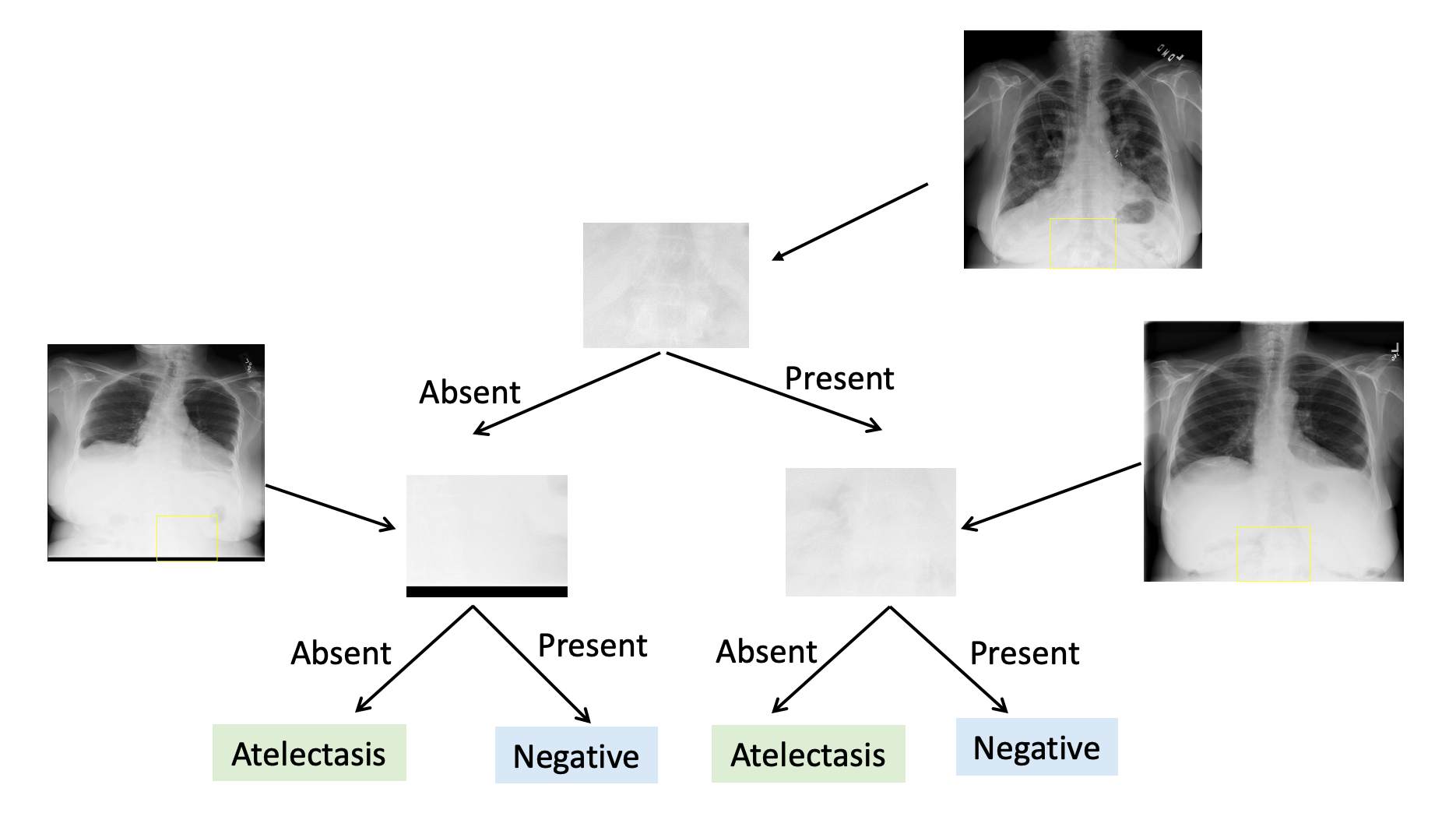


Figure 2. Global explanation of the NPT classifier’s decision-making process for detecting atelectasis (IC=3) with PA view CXRs in Chest X-ray 14.


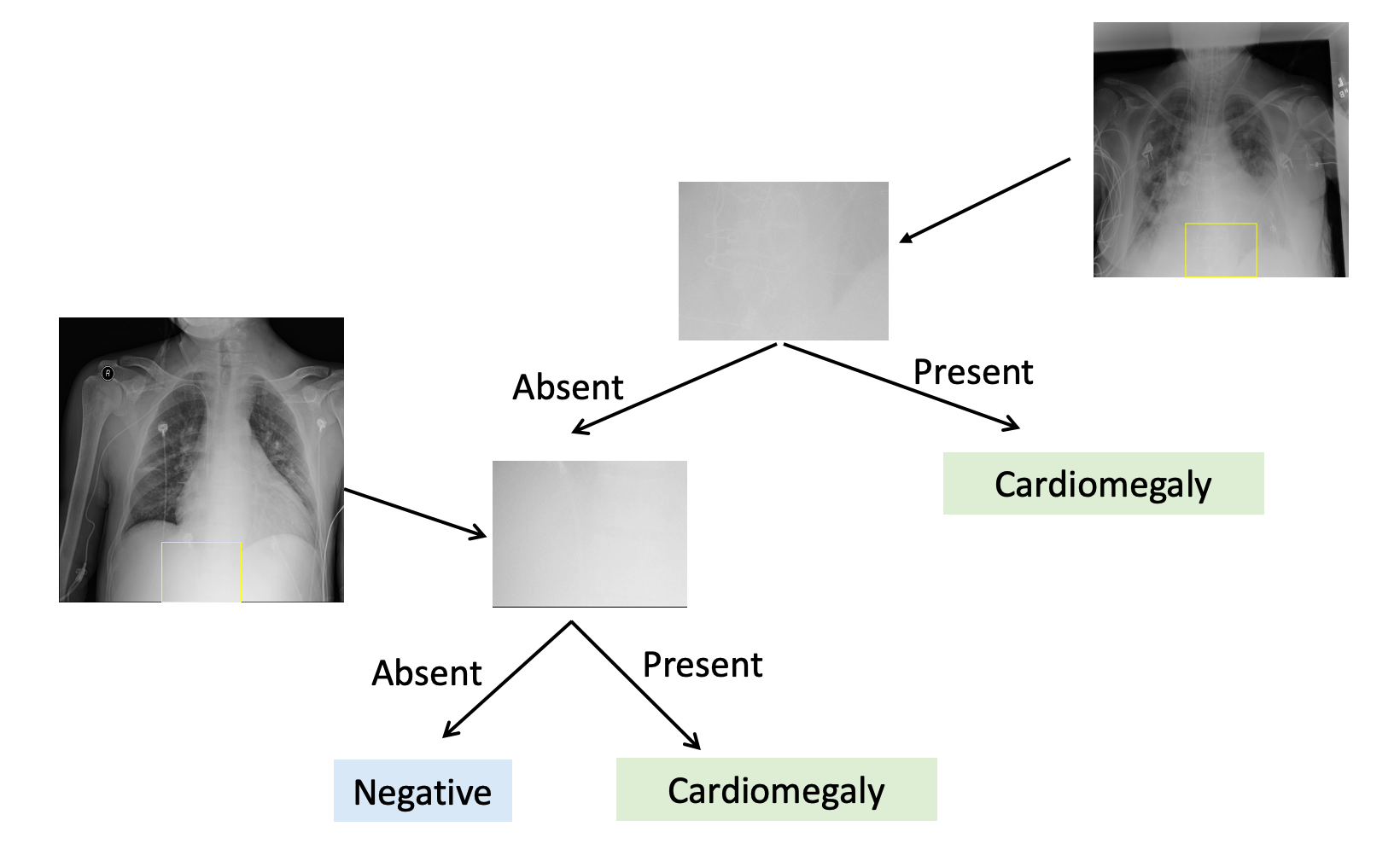


Figure 3. Global explanation of the NPT classifier’s decision-making process for detecting Cardiomegaly (IC=3) with AP view CXRs in Chest X-ray 14.


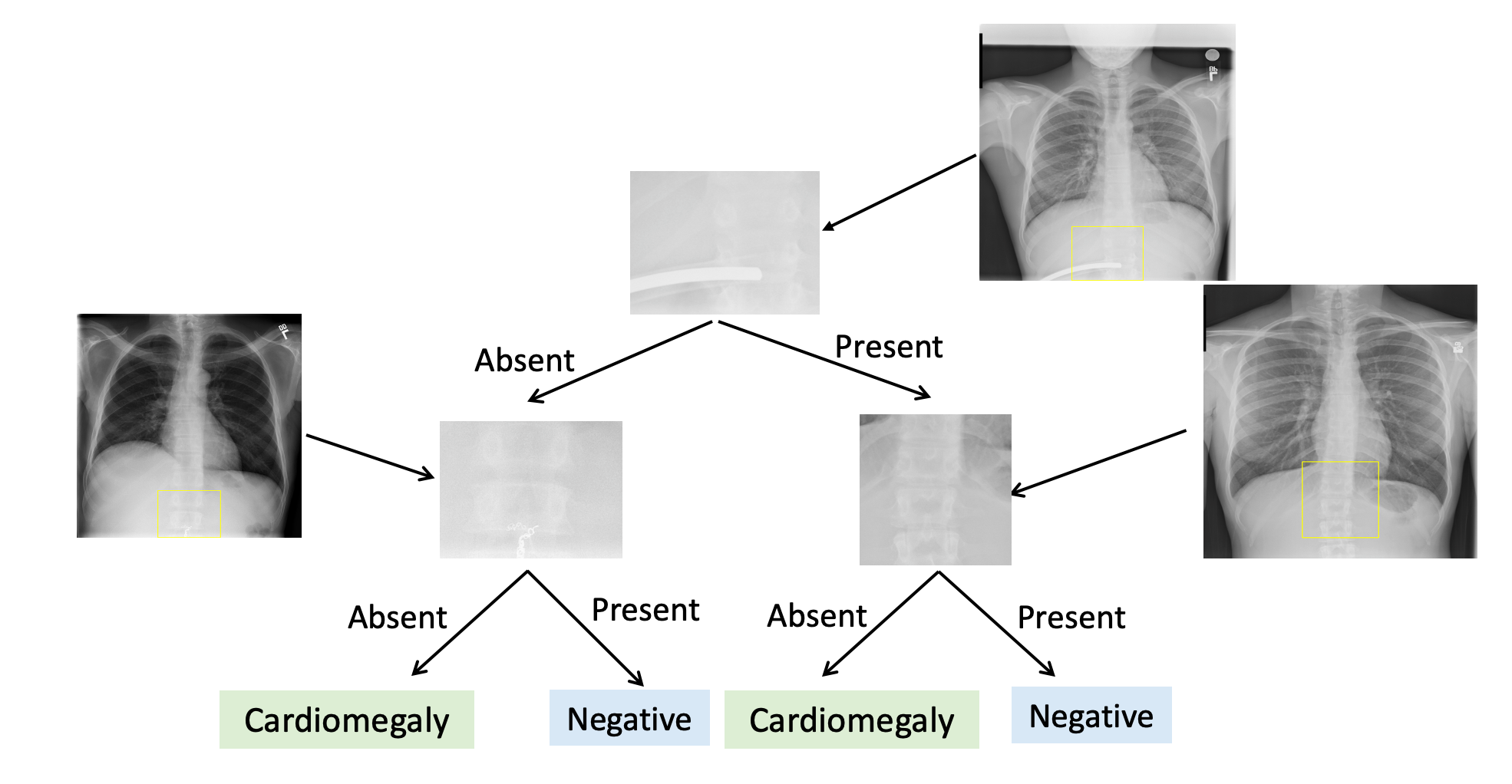


Figure 4. Global explanation of the NPT classifier’s decision-making process for detecting Cardiomegaly (IC=3) with PA view CXRs in Chest X-ray 14.


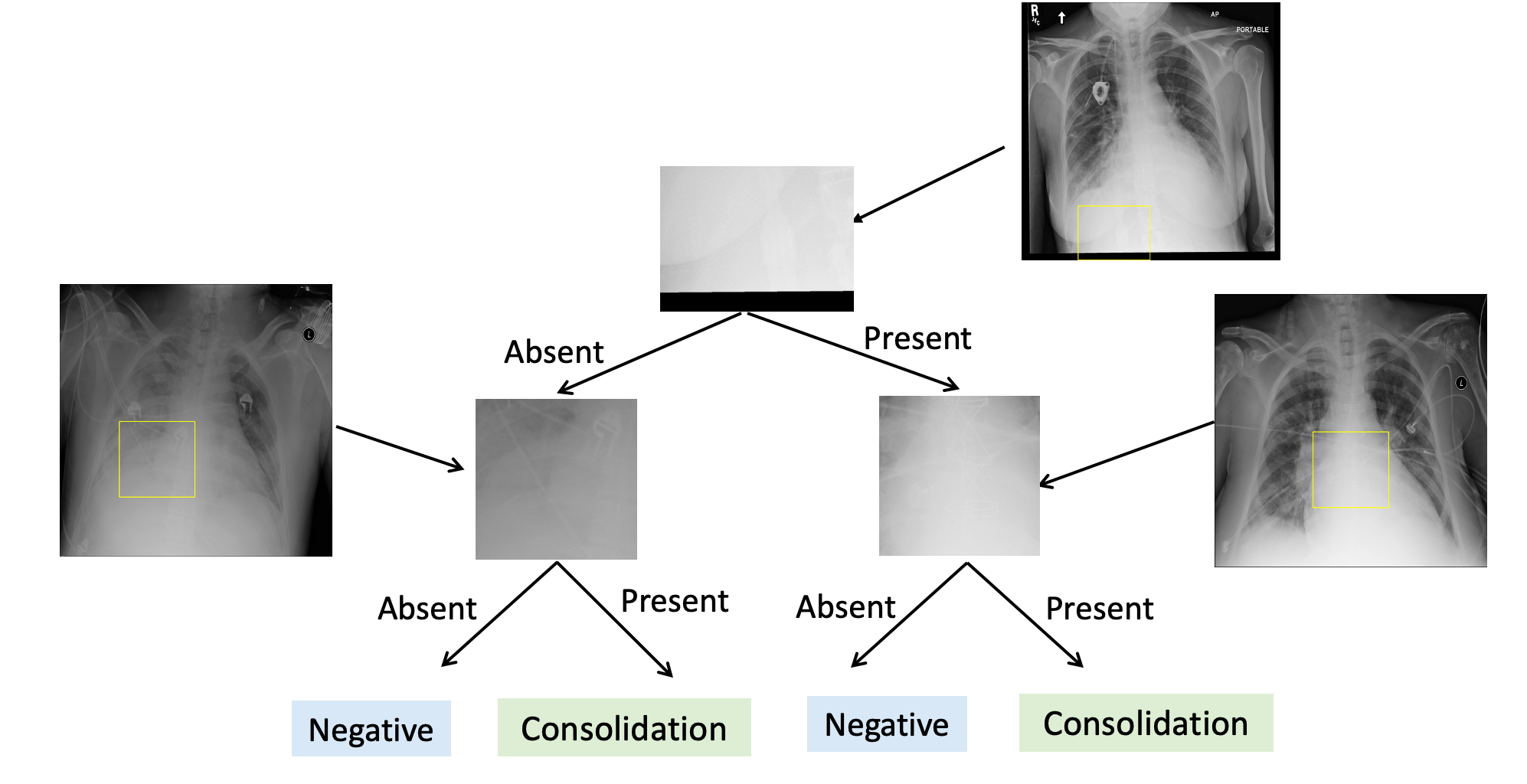


Figure 5. Global explanation of the NPT classifier’s decision-making process for detecting Consolidation (IC=3) with AP view CXRs in Chest X-ray 14.


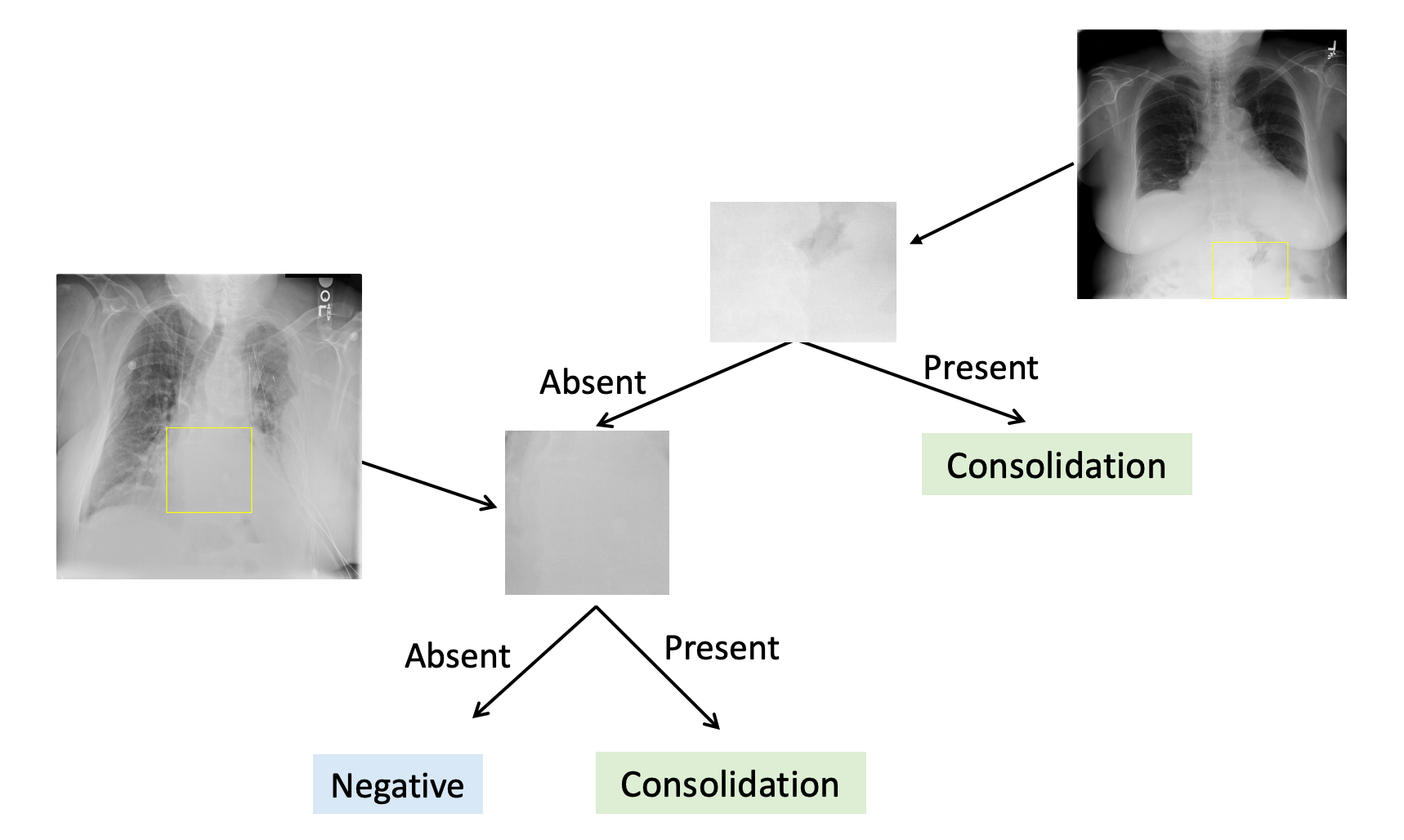


Figure 6. Global explanation of the NPT classifier’s decision-making process for detecting Consolidation (IC=3) with PA view CXRs in Chest X-ray 14.


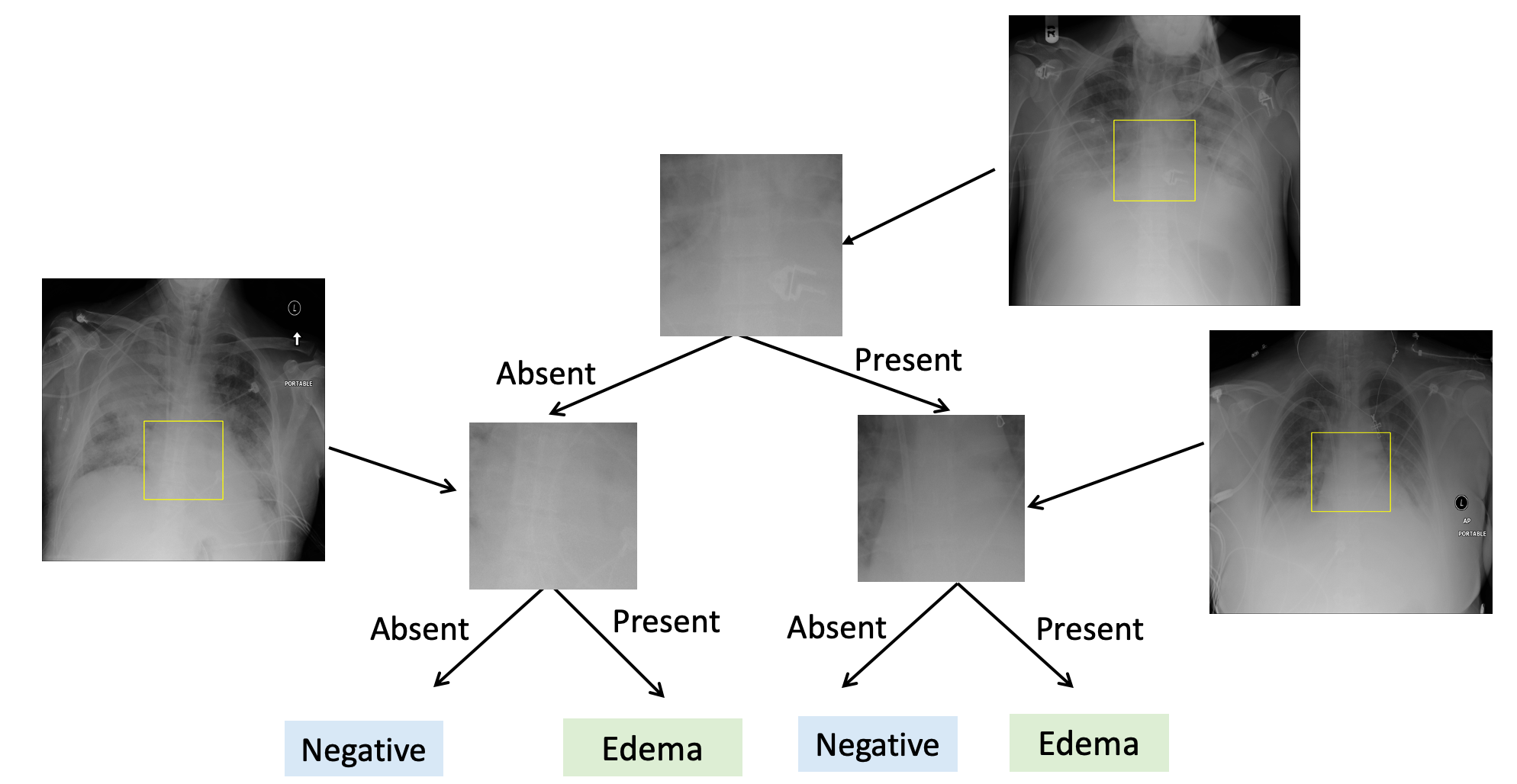


Figure 7. Global explanation of the NPT classifier’s decision-making process for detecting Edema (IC=3) with AP view CXRs in Chest X-ray 14.


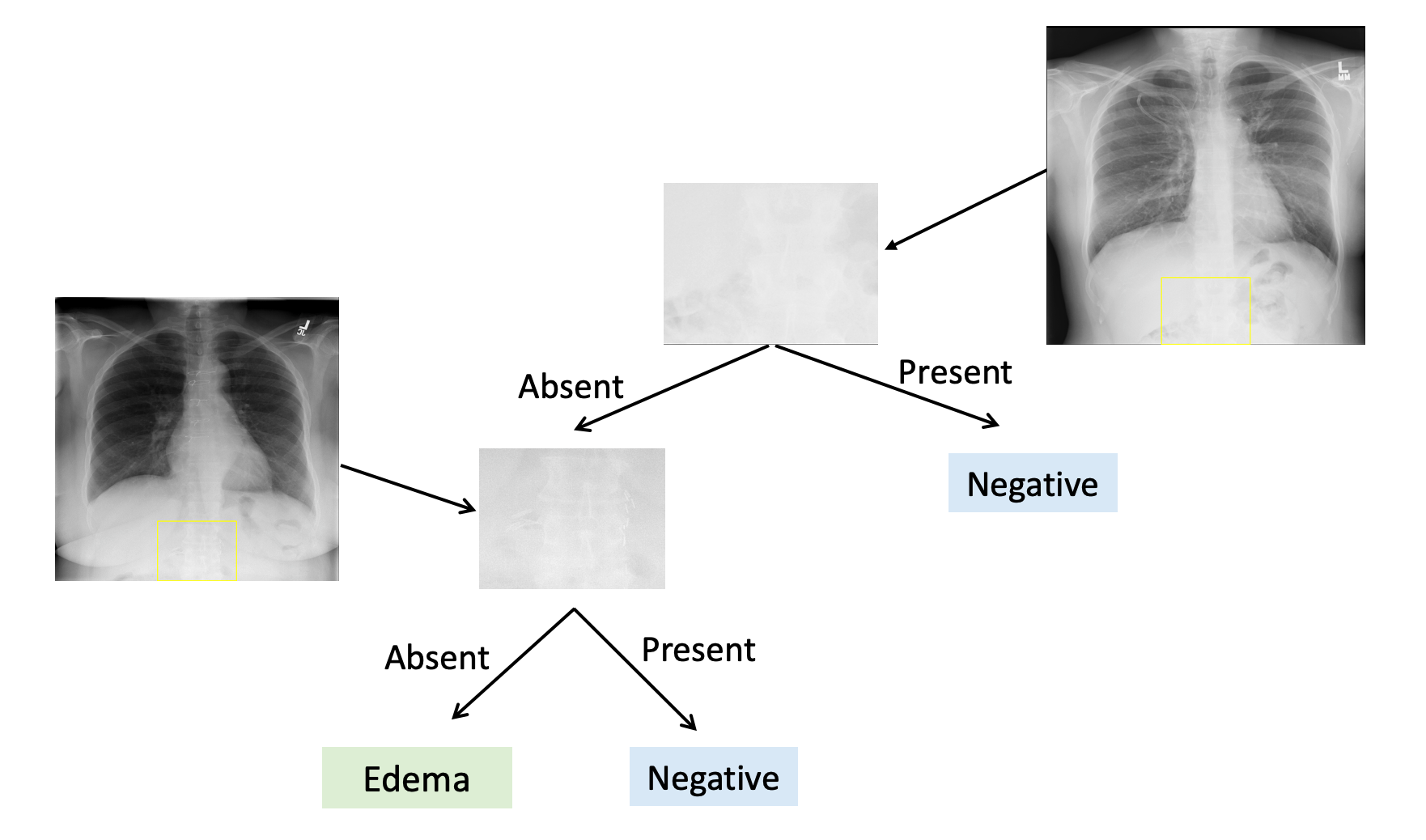


Figure 8. Global explanation of the NPT classifier’s decision-making process for detecting Edema (IC=3) with PA view CXRs in Chest X-ray 14.


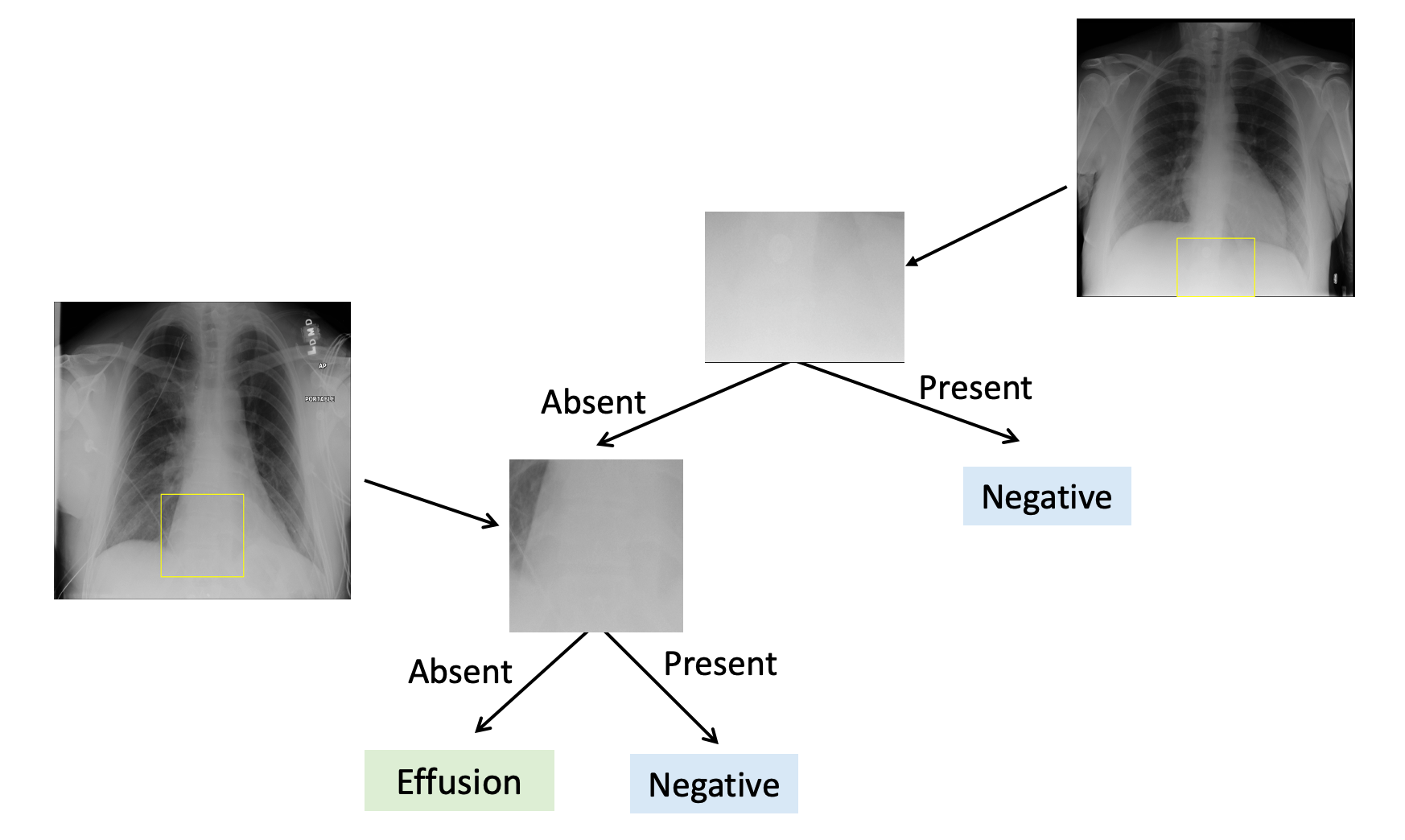


Figure 9. Global explanation of the NPT classifier’s decision-making process for detecting Effusion (IC=3) with AP view CXRs in Chest X-ray 14.


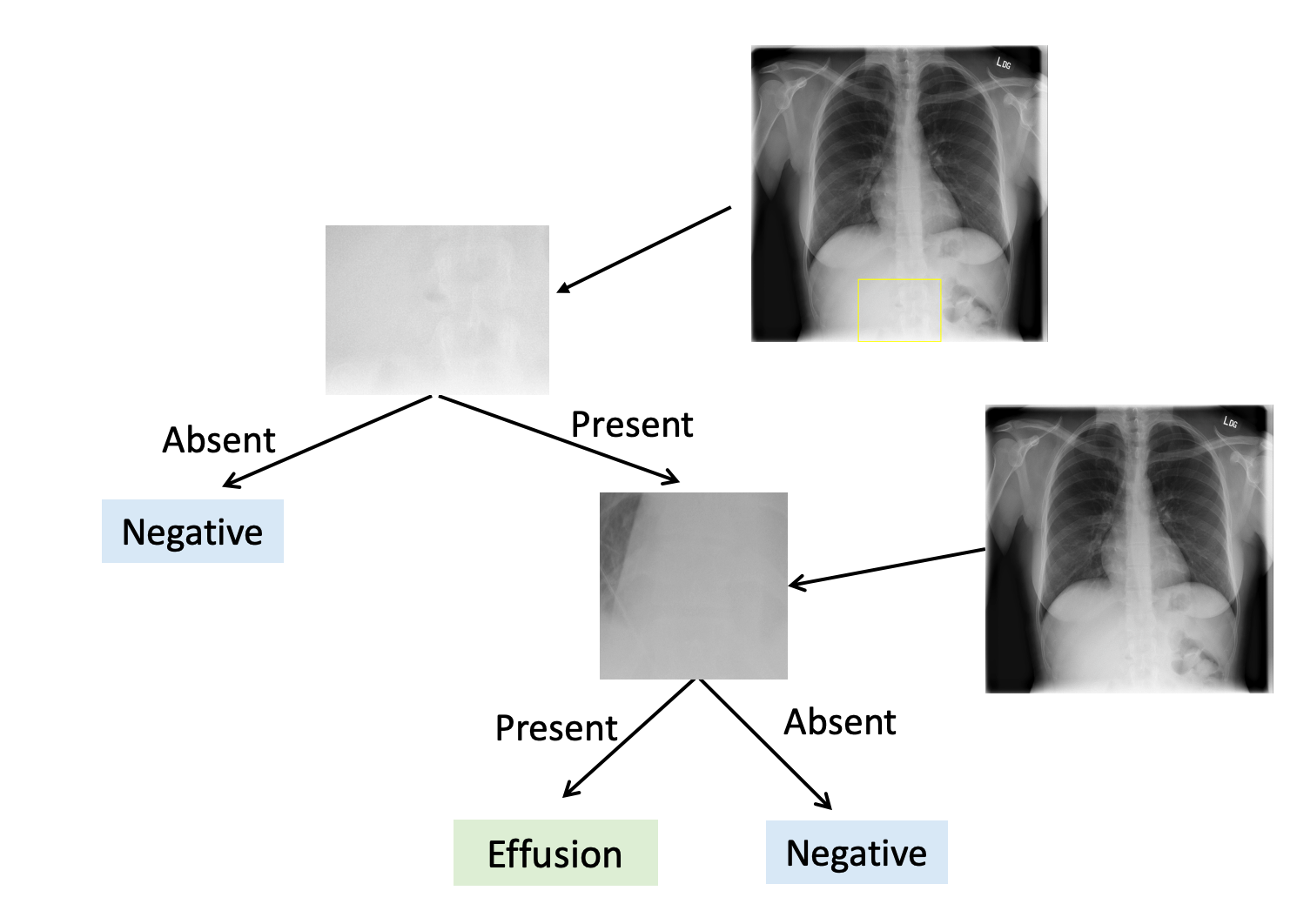


Figure 10. Global explanation of the NPT classifier’s decision-making process for detecting Effusion (IC=3) with PA view CXRs in Chest X-ray 14.


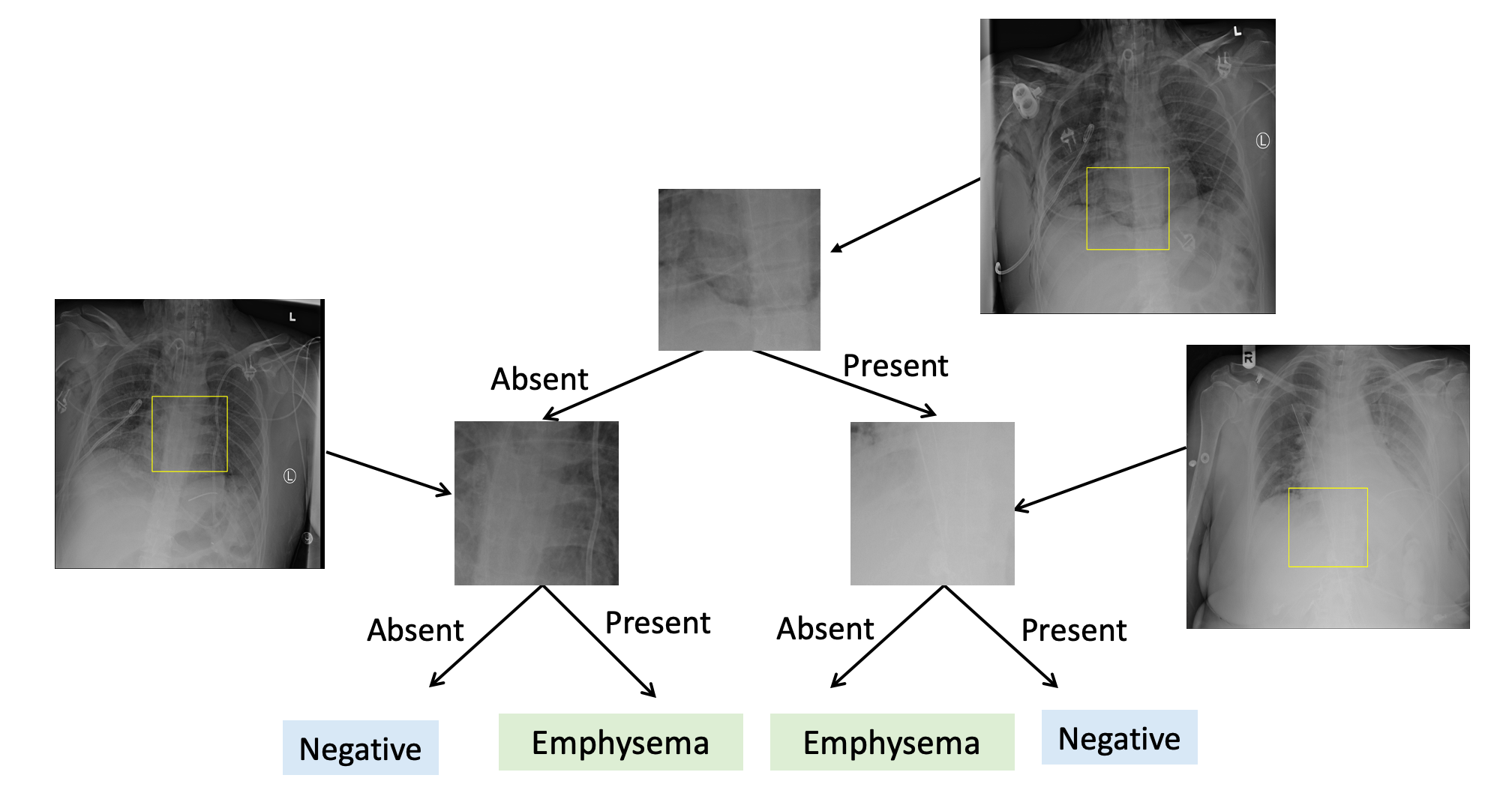


Figure 11. Global explanation of the NPT classifier’s decision-making process for detecting Emphysema (IC=3) with AP view CXRs in Chest X-ray 14.


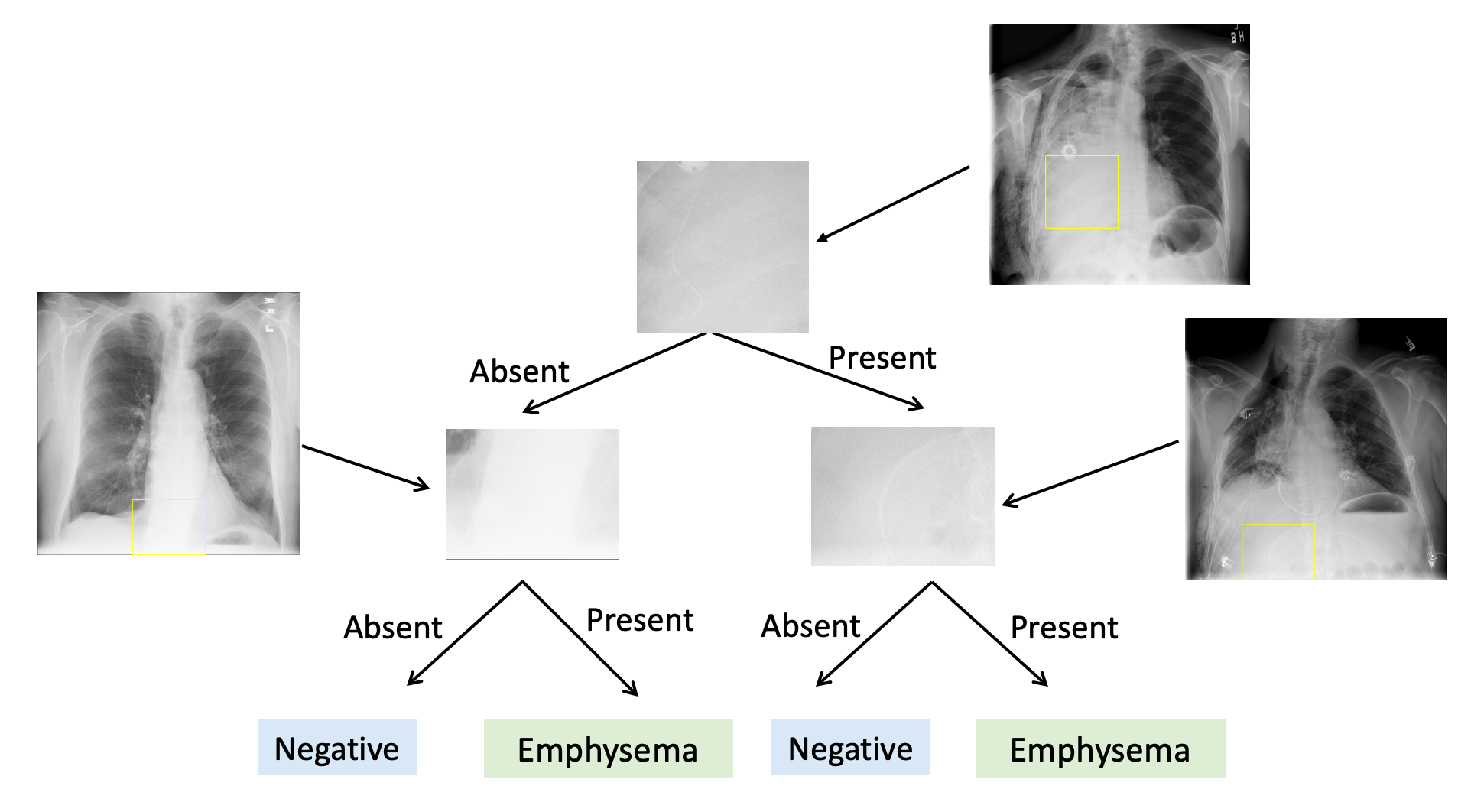


Figure 12. Global explanation of the NPT classifier’s decision-making process for detecting Emphysema (IC=3) with PA view CXRs in Chest X-ray 14.


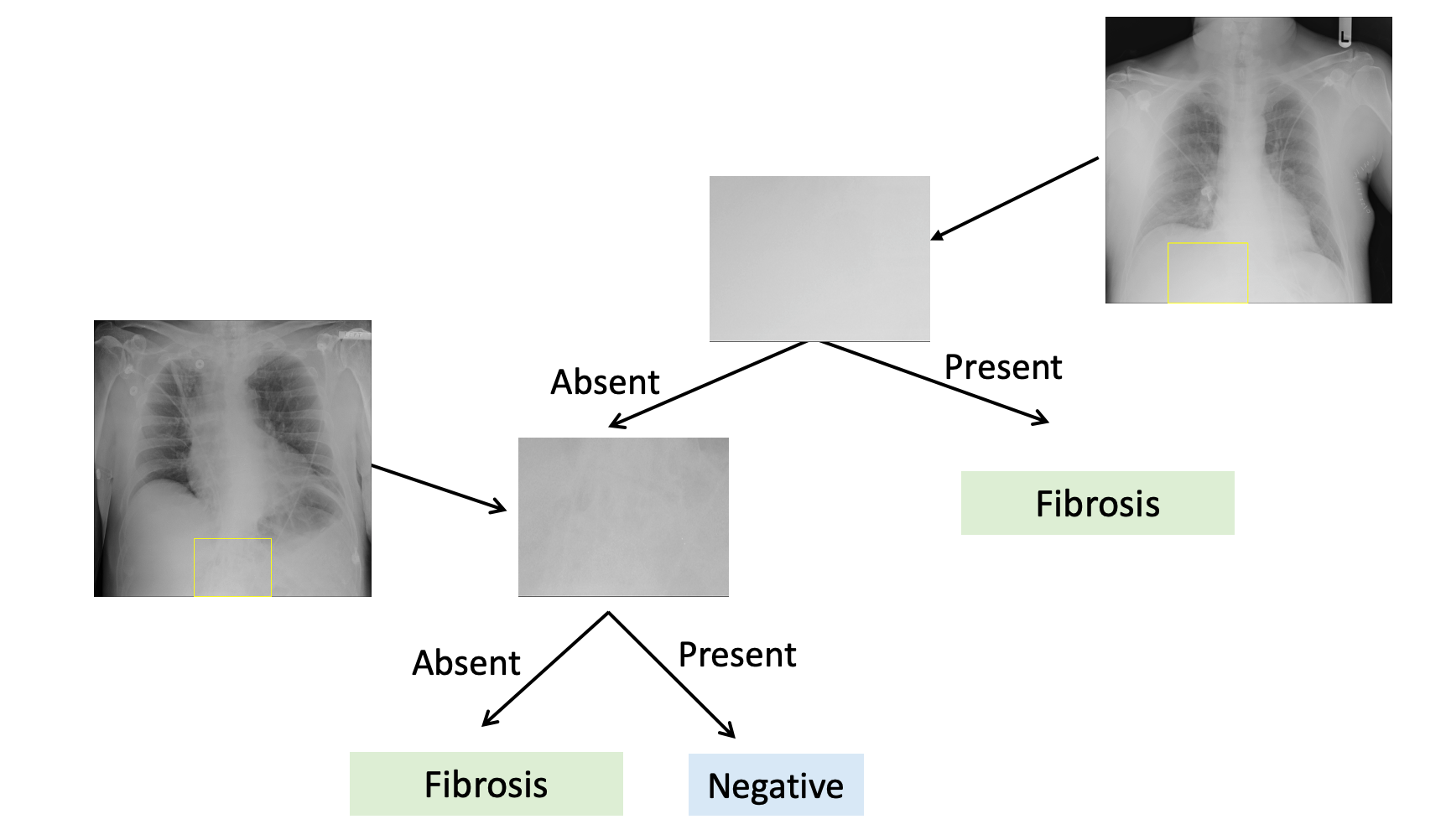


Figure 13. Global explanation of the NPT classifier’s decision-making process for detecting Fibrosis (IC=3) with AP view CXRs in Chest X-ray 14.


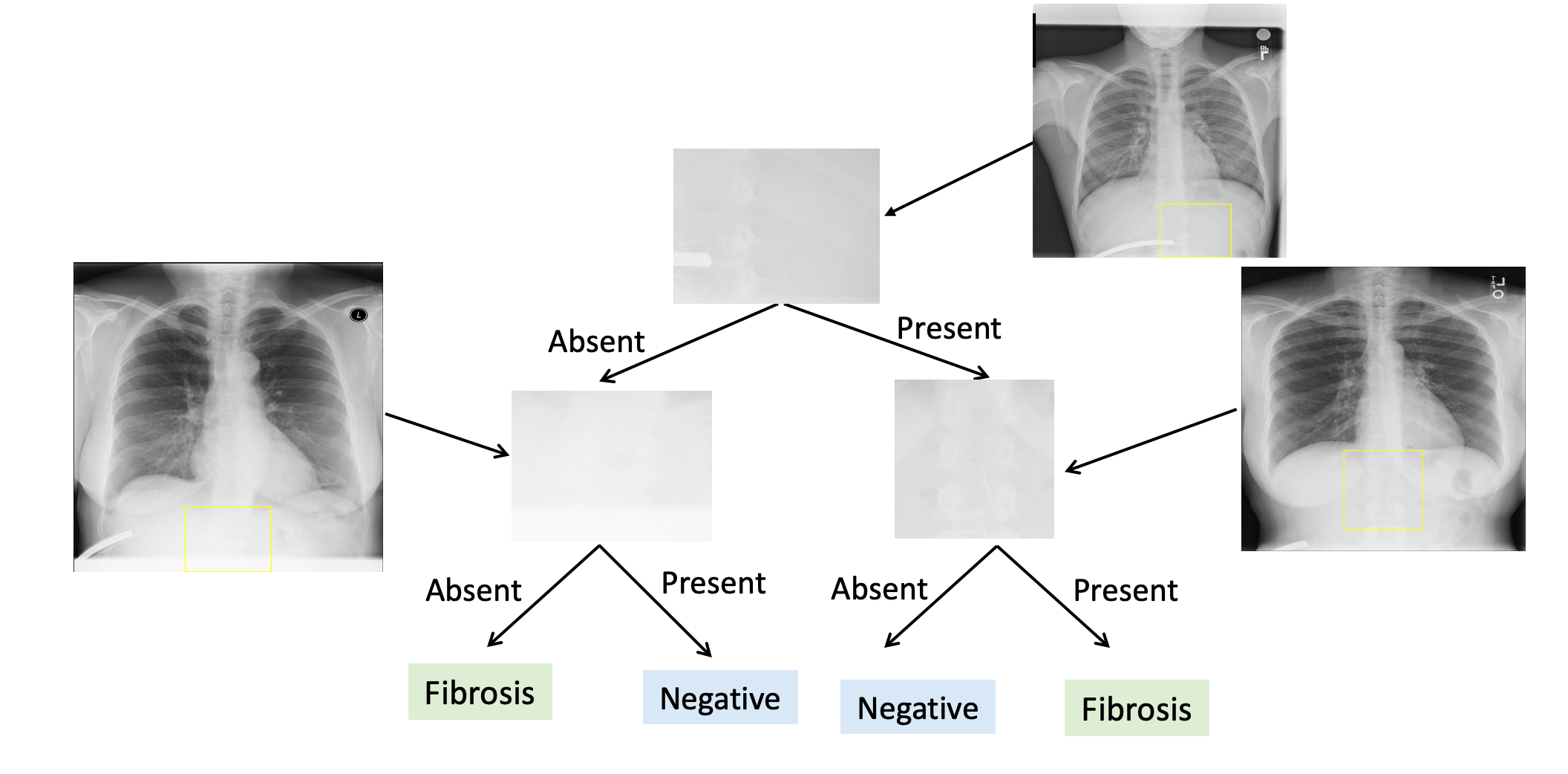


Figure 14. Global explanation of the NPT classifier’s decision-making process for detecting Fibrosis (IC=3) with PA view CXRs in Chest X-ray 14.


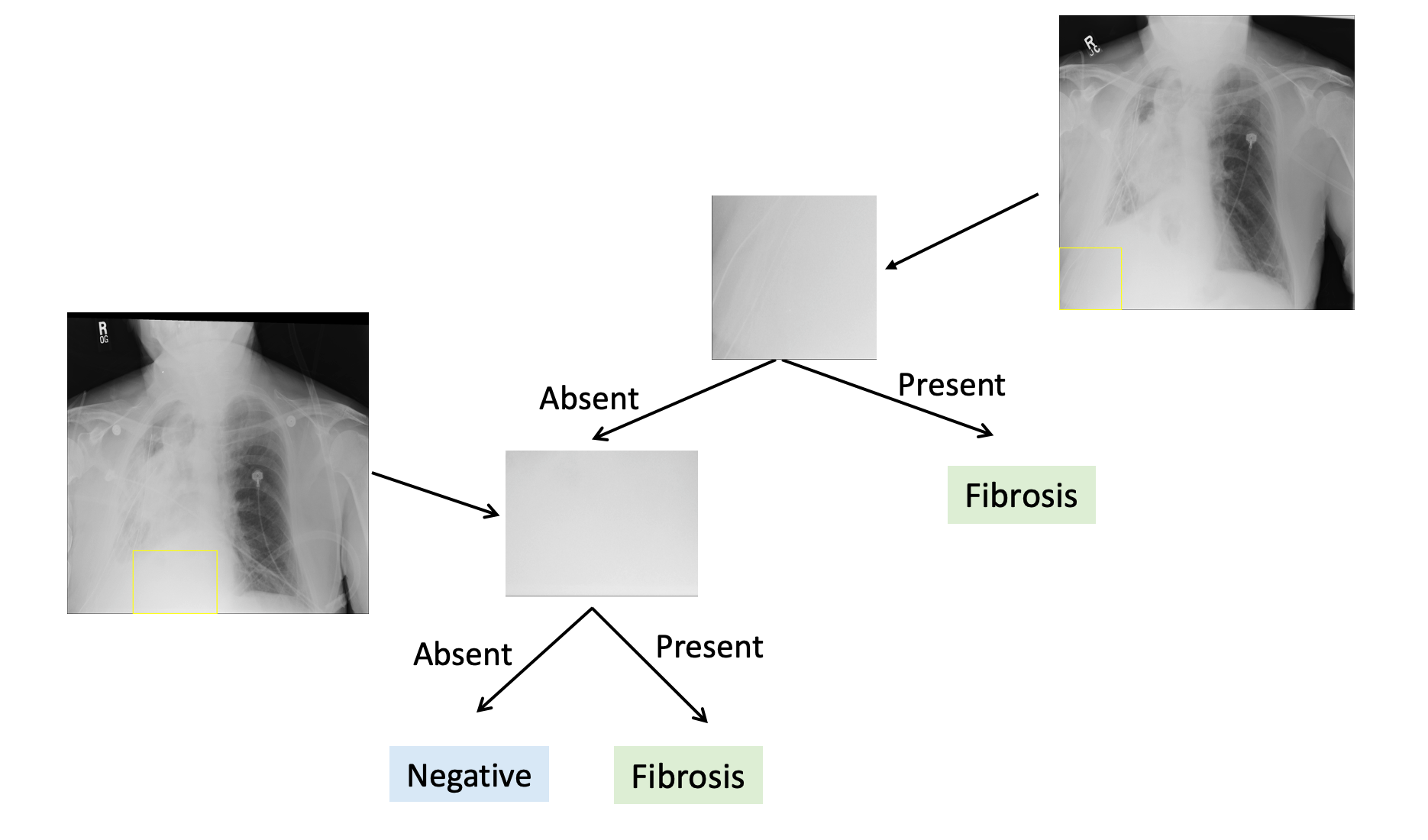


Figure 15. Global explanation of the NPT classifier’s decision-making process for detecting Hernia (IC=3) with AP view CXRs in Chest X-ray 14.


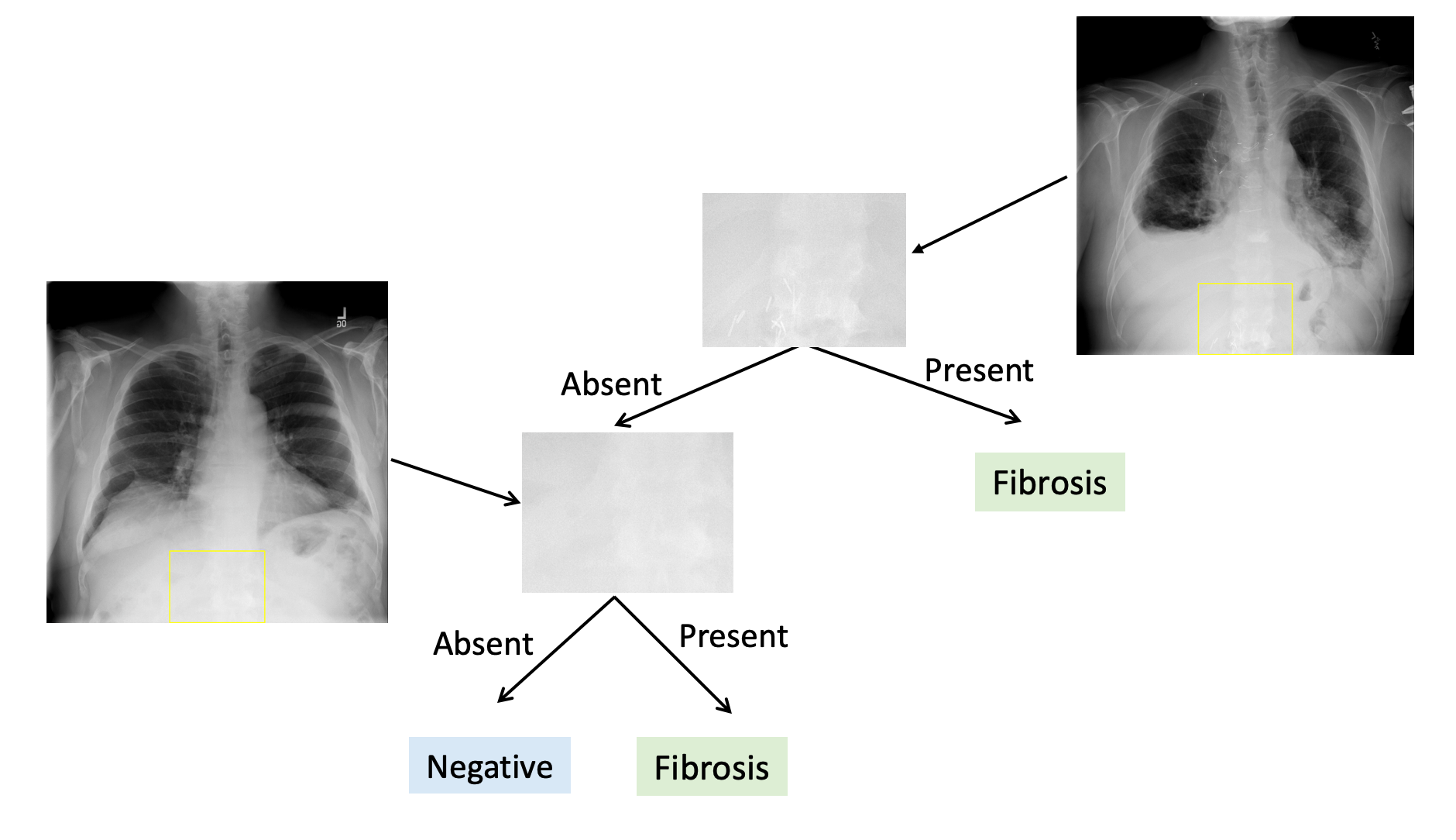


Figure 16. Global explanation of the NPT classifier’s decision-making process for detecting Hernia (IC=3) with PA view CXRs in Chest X-ray 14.


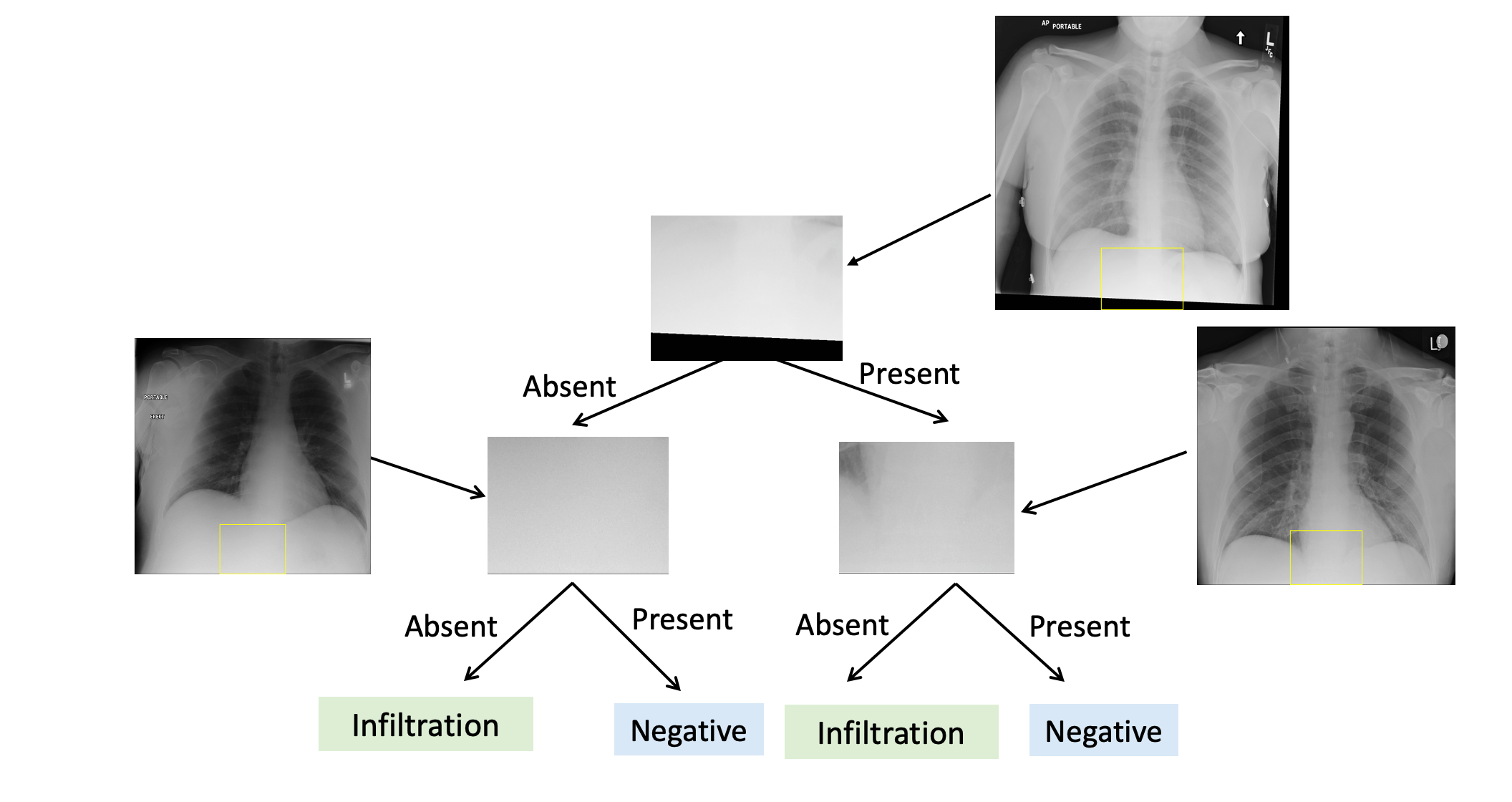


Figure 17. Global explanation of the NPT classifier’s decision-making process for detecting Infiltration (IC=3) with AP view CXRs in Chest X-ray 14.


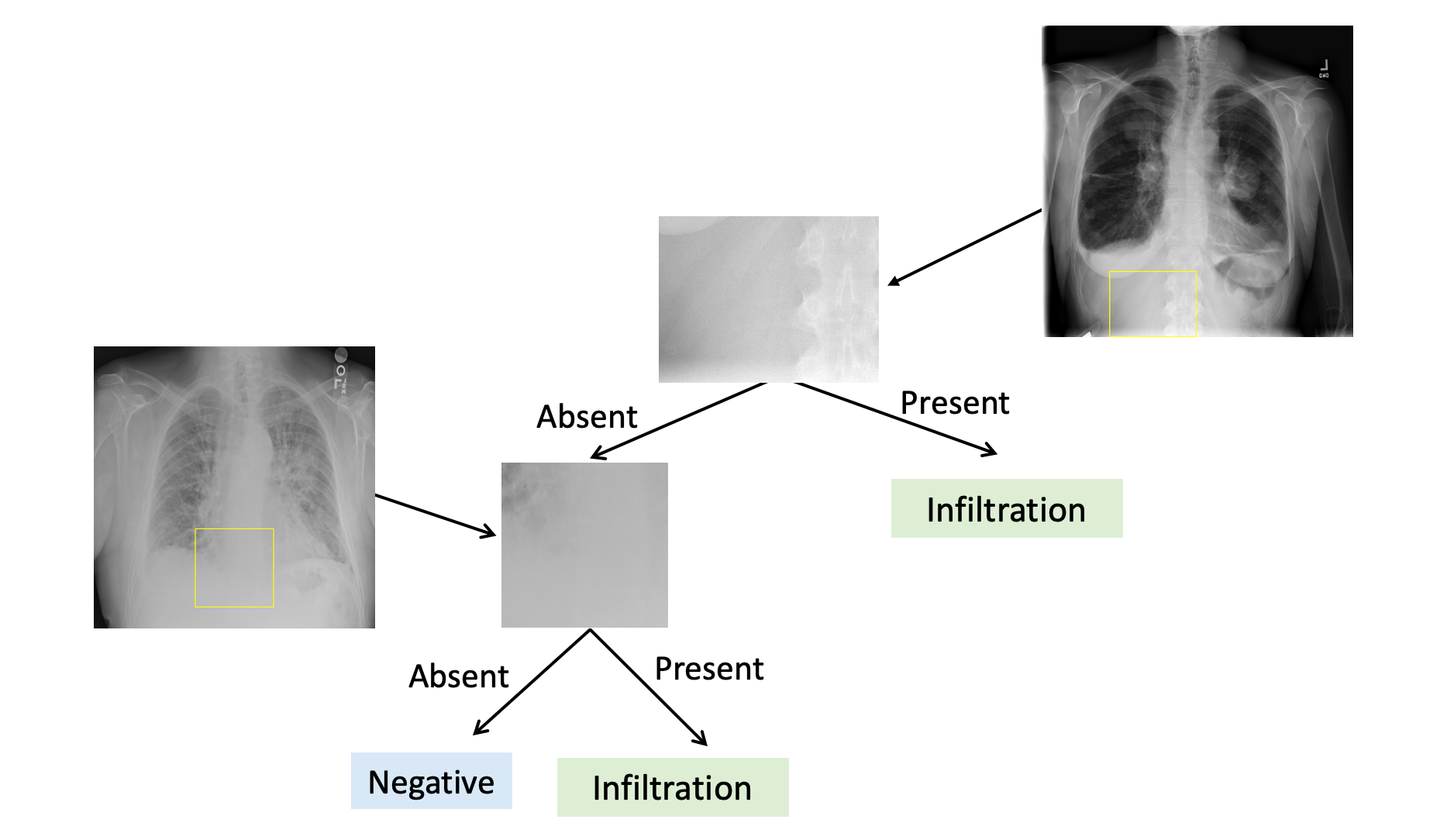


Figure 18. Global explanation of the NPT classifier’s decision-making process for detecting Infiltration (IC=3) with PA view CXRs in Chest X-ray 14.


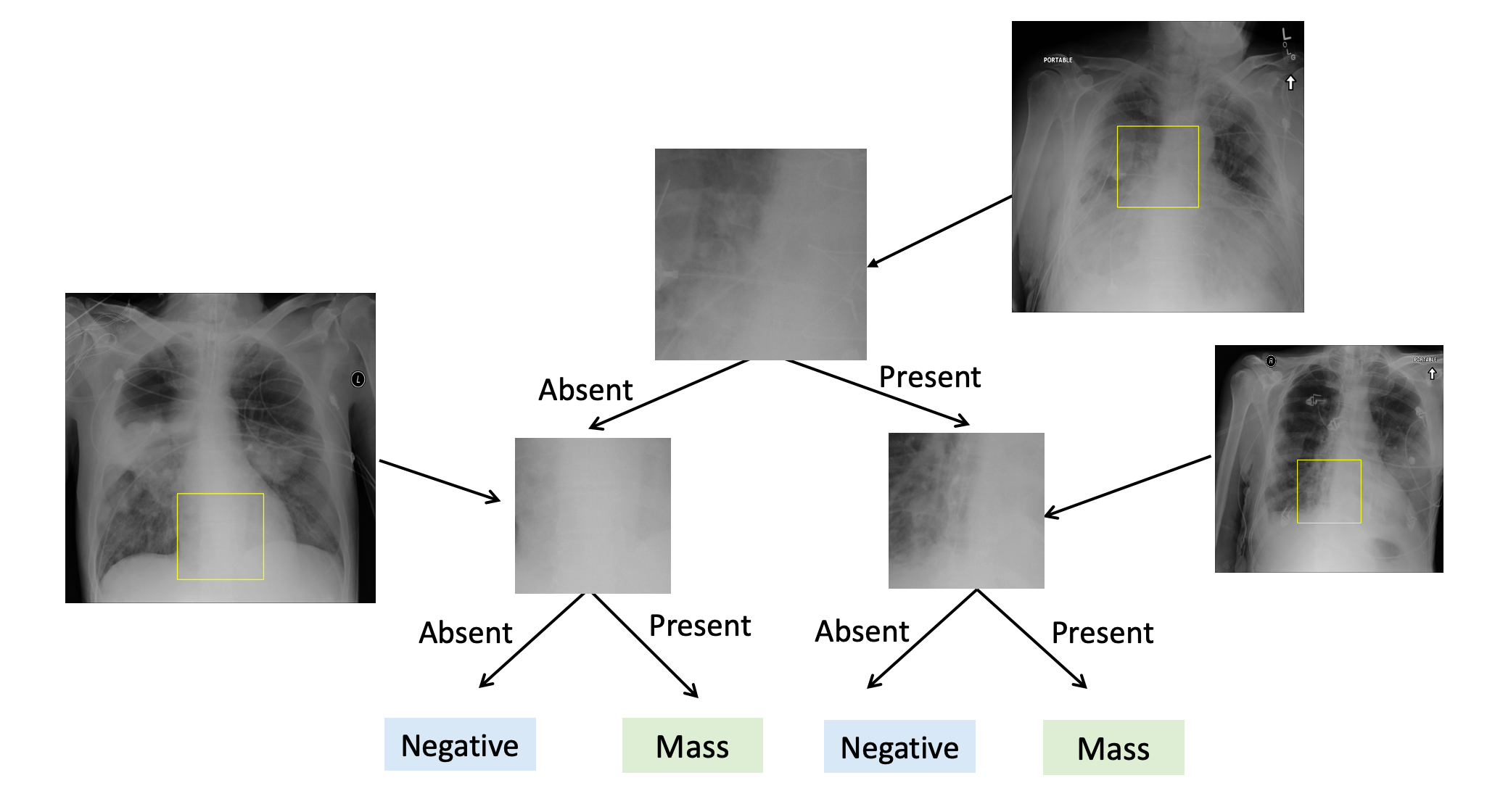


Figure 19. Global explanation of the NPT classifier’s decision-making process for detecting Mass (IC=3) with AP view CXRs in Chest X-ray 14.


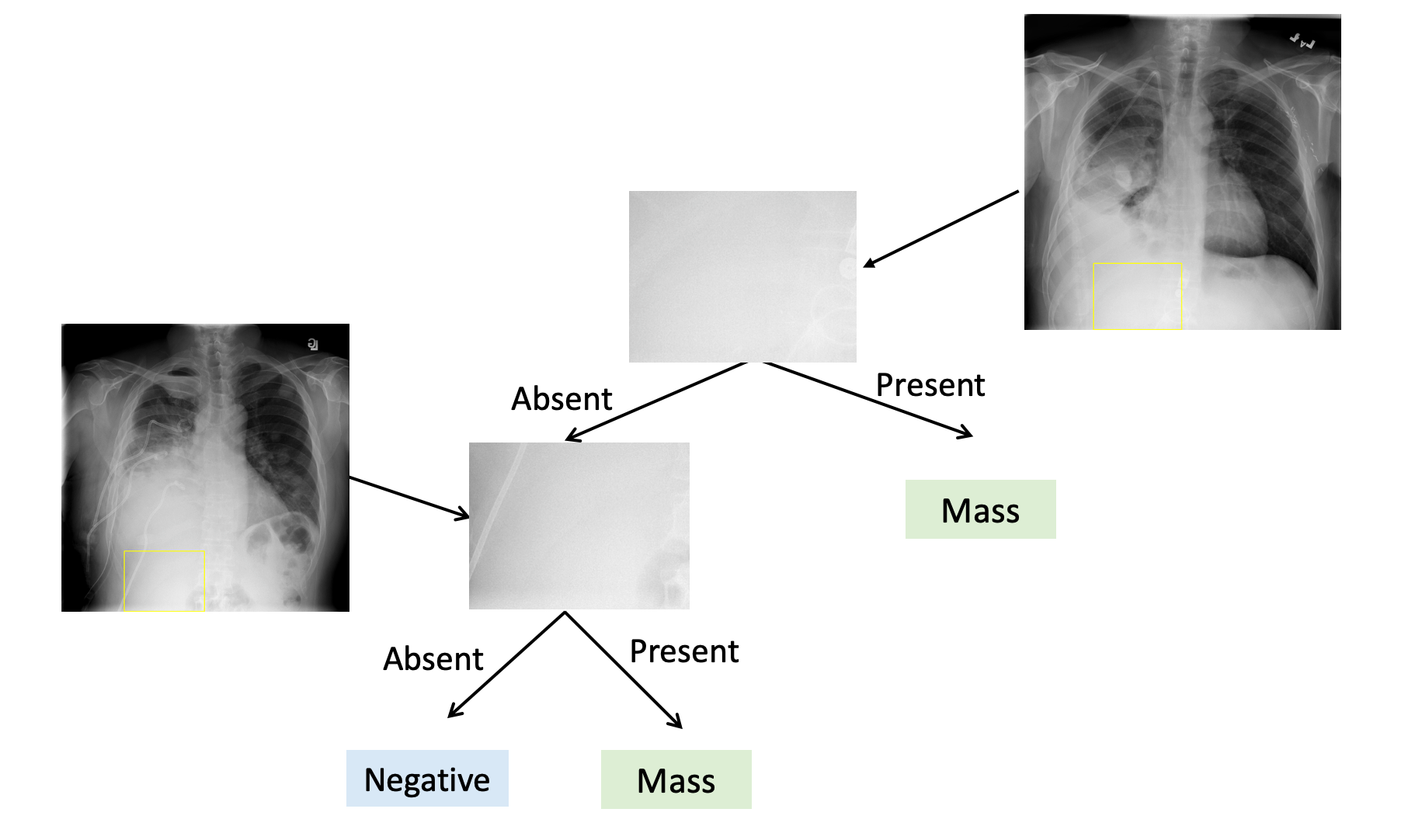


Figure 20. Global explanation of the NPT classifier’s decision-making process for detecting Mass (IC=3) with PA view CXRs in Chest X-ray 14.


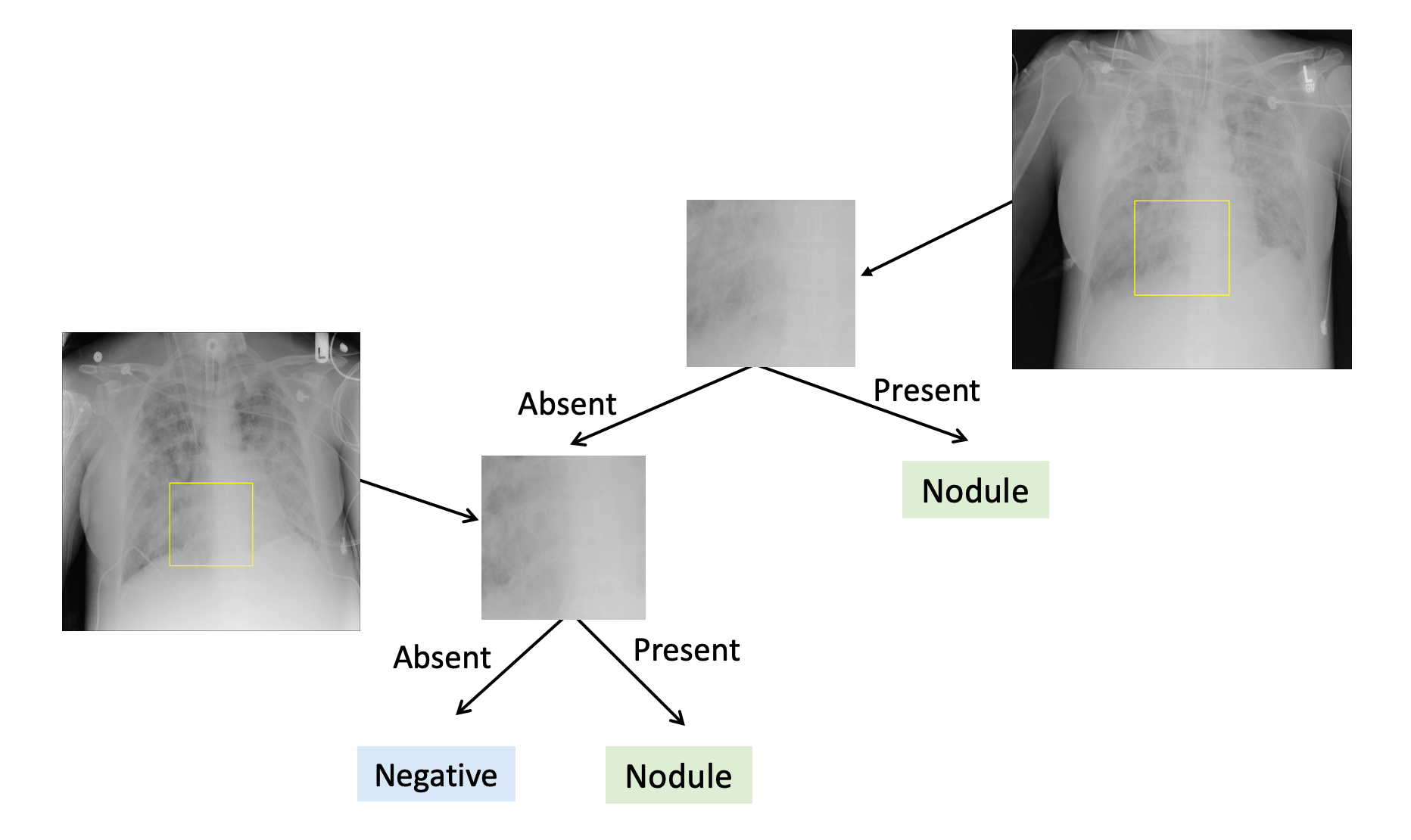


Figure 21. Global explanation of the NPT classifier’s decision-making process for detecting Nodule (IC=3) with AP view CXRs in Chest X-ray 14.


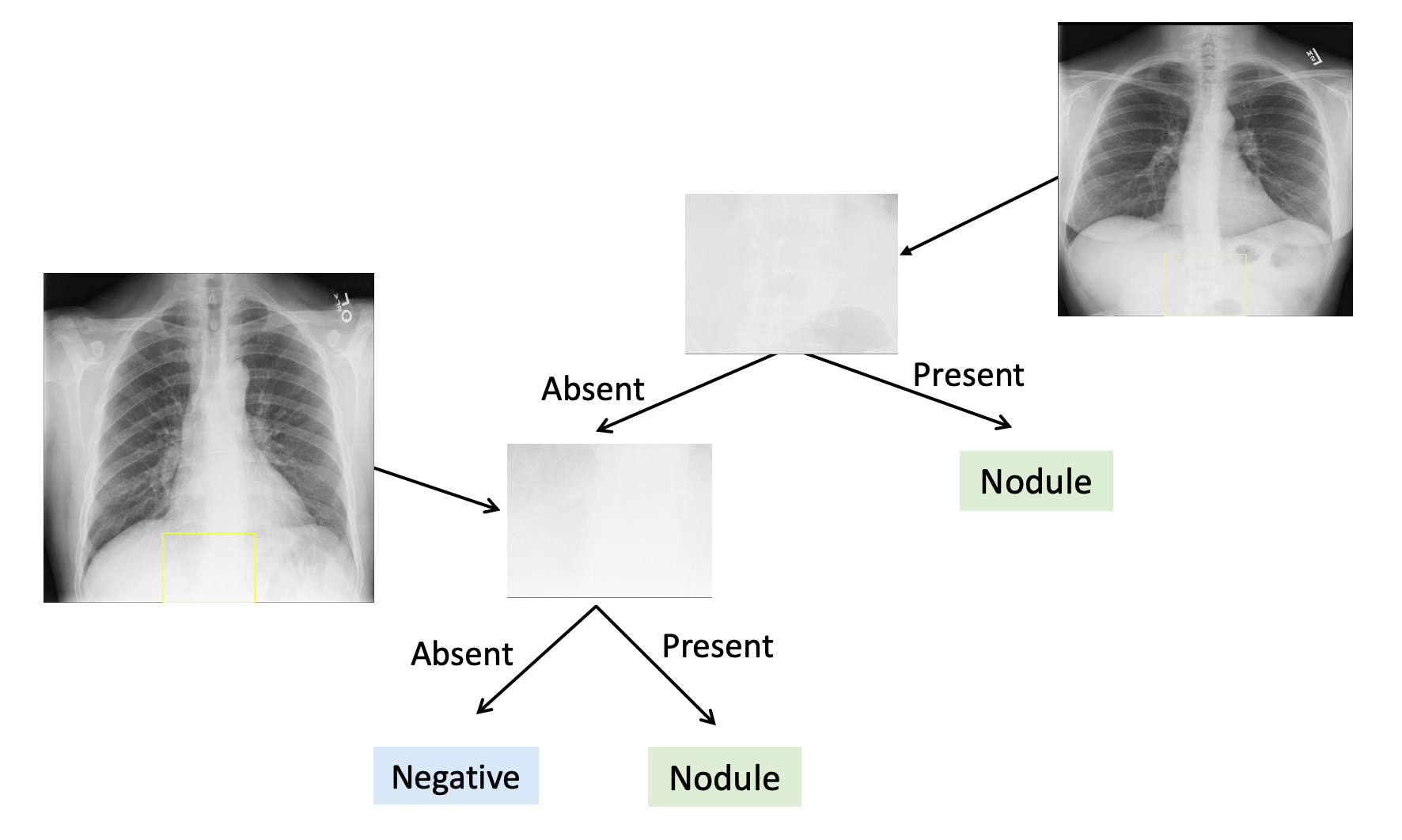


Figure 22. Global explanation of the NPT classifier’s decision-making process for detecting Nodule (IC=3) with PA view CXRs in Chest X-ray 14.


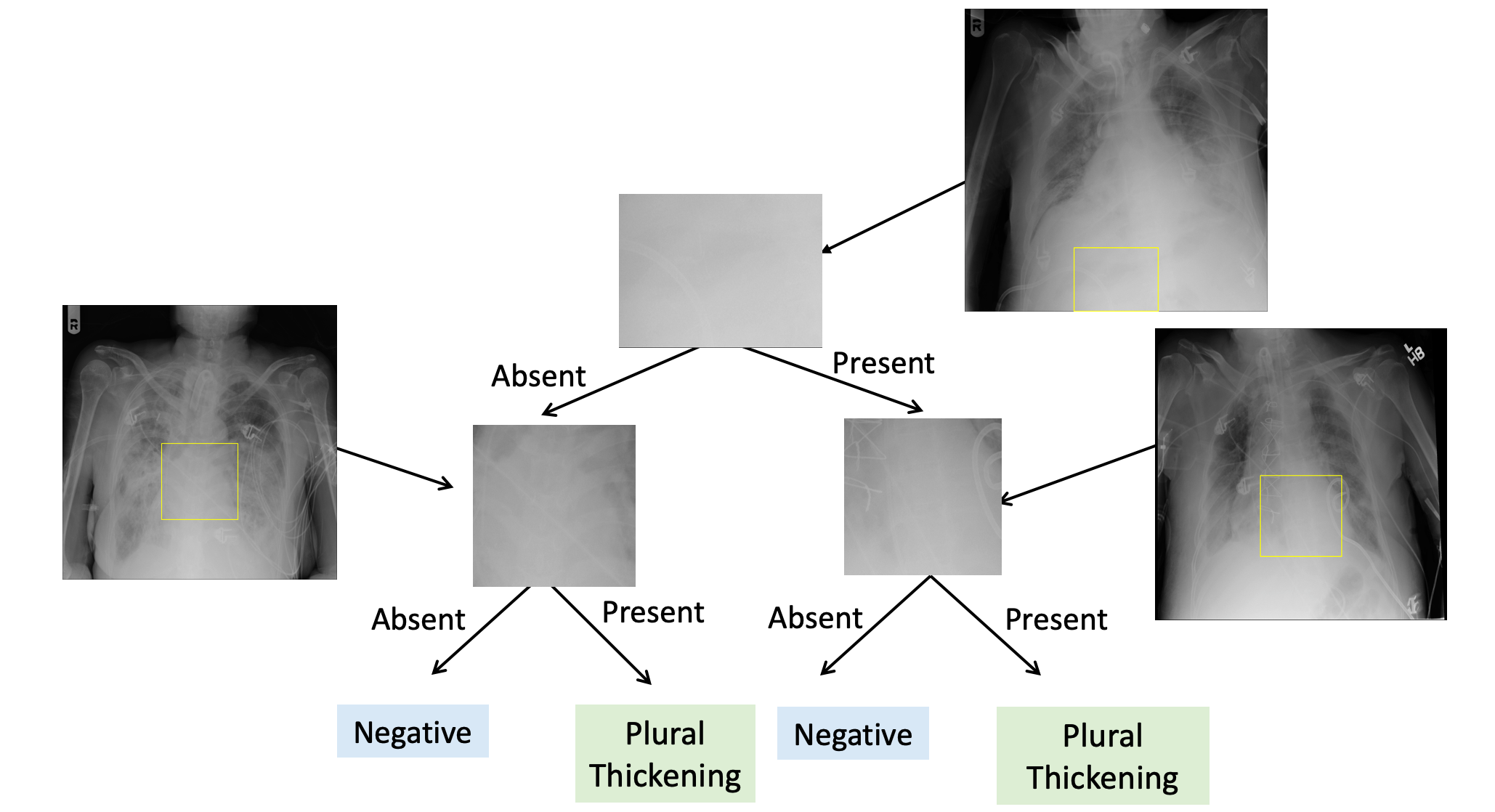


Figure 23. Global explanation of the NPT classifier’s decision-making process for detecting Plural Thickening (IC=3) with AP view CXRs in Chest X-ray 14.


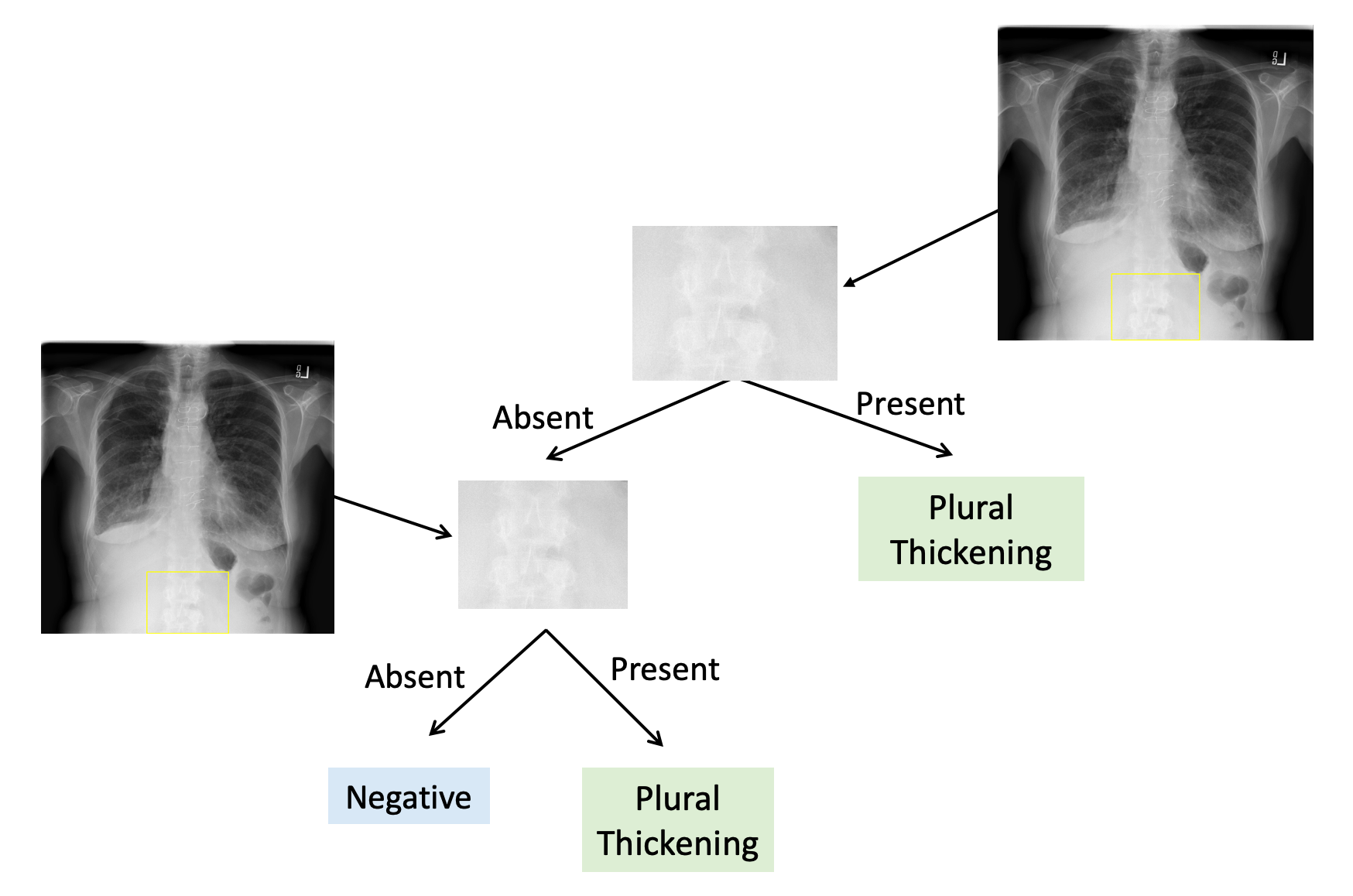


Figure 24. Global explanation of the NPT classifier’s decision-making process for detecting Plural Thickening (IC=3) with PA view CXRs in Chest X-ray 14.


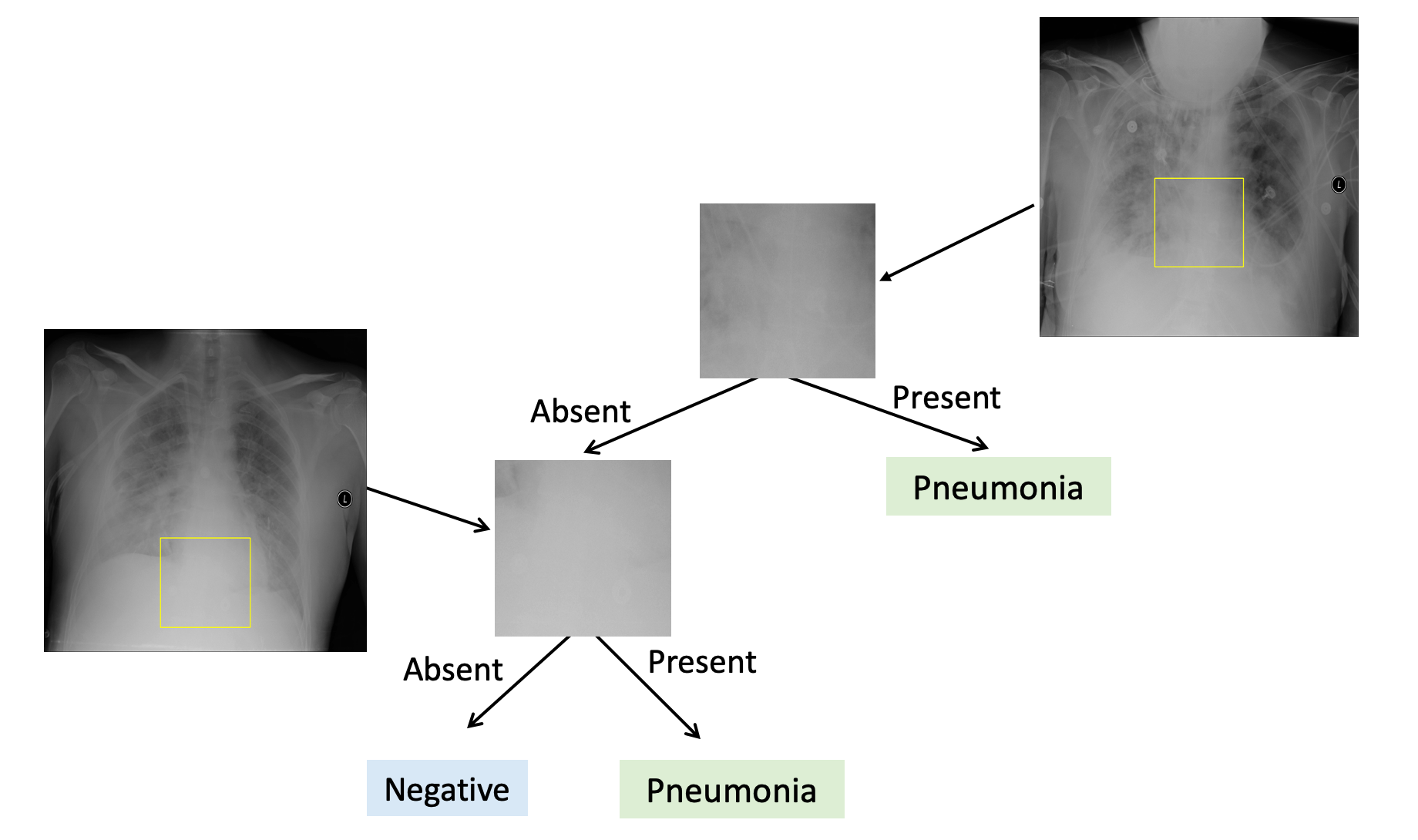


Figure 25. Global explanation of the NPT classifier’s decision-making process for detecting Pneumonia (IC=3) with AP view CXRs in Chest X-ray 14.


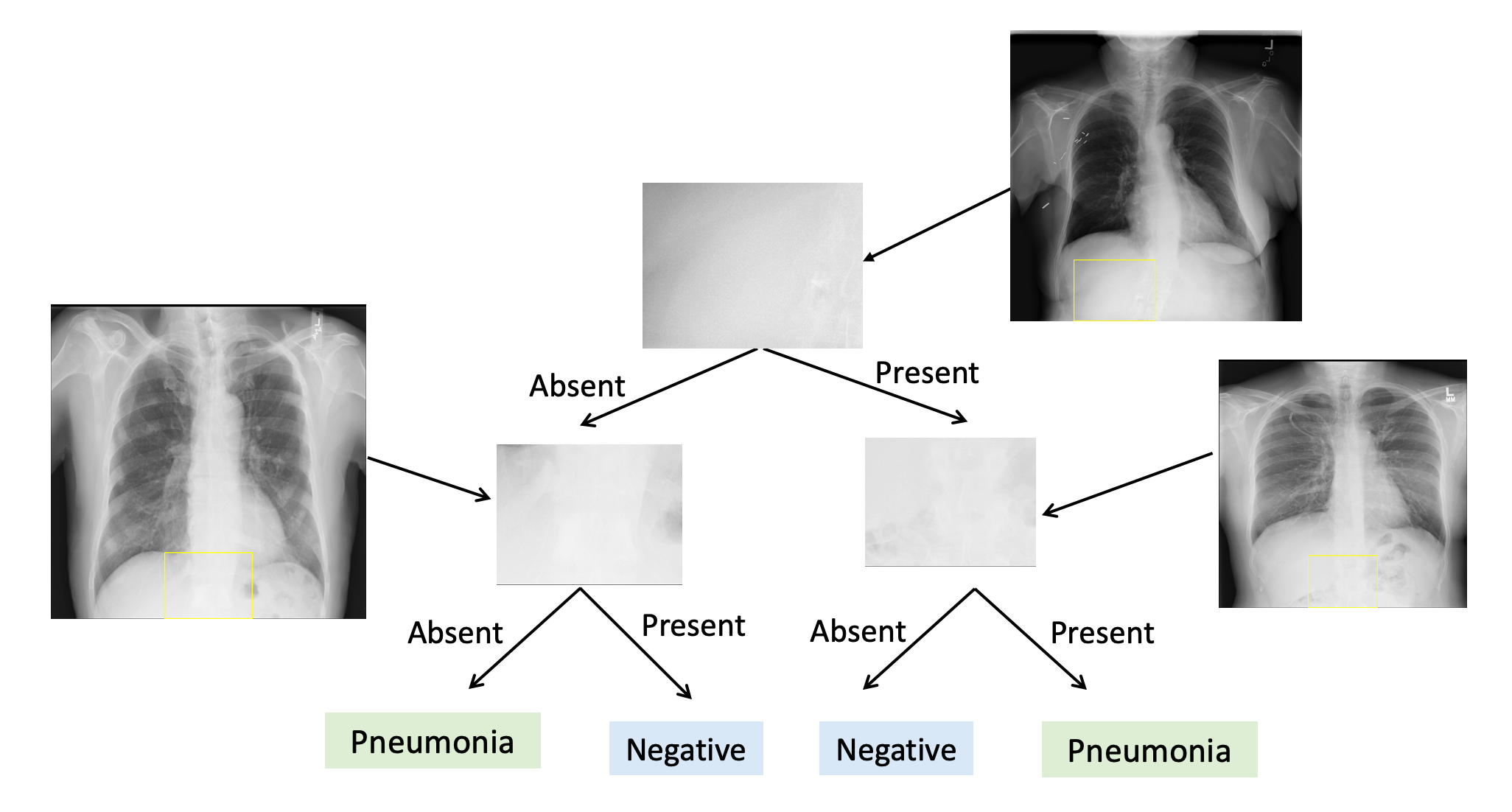


Figure 26. Global explanation of the NPT classifier’s decision-making process for detecting Pneumonia (IC=3) with PA view CXRs in Chest X-ray 14.


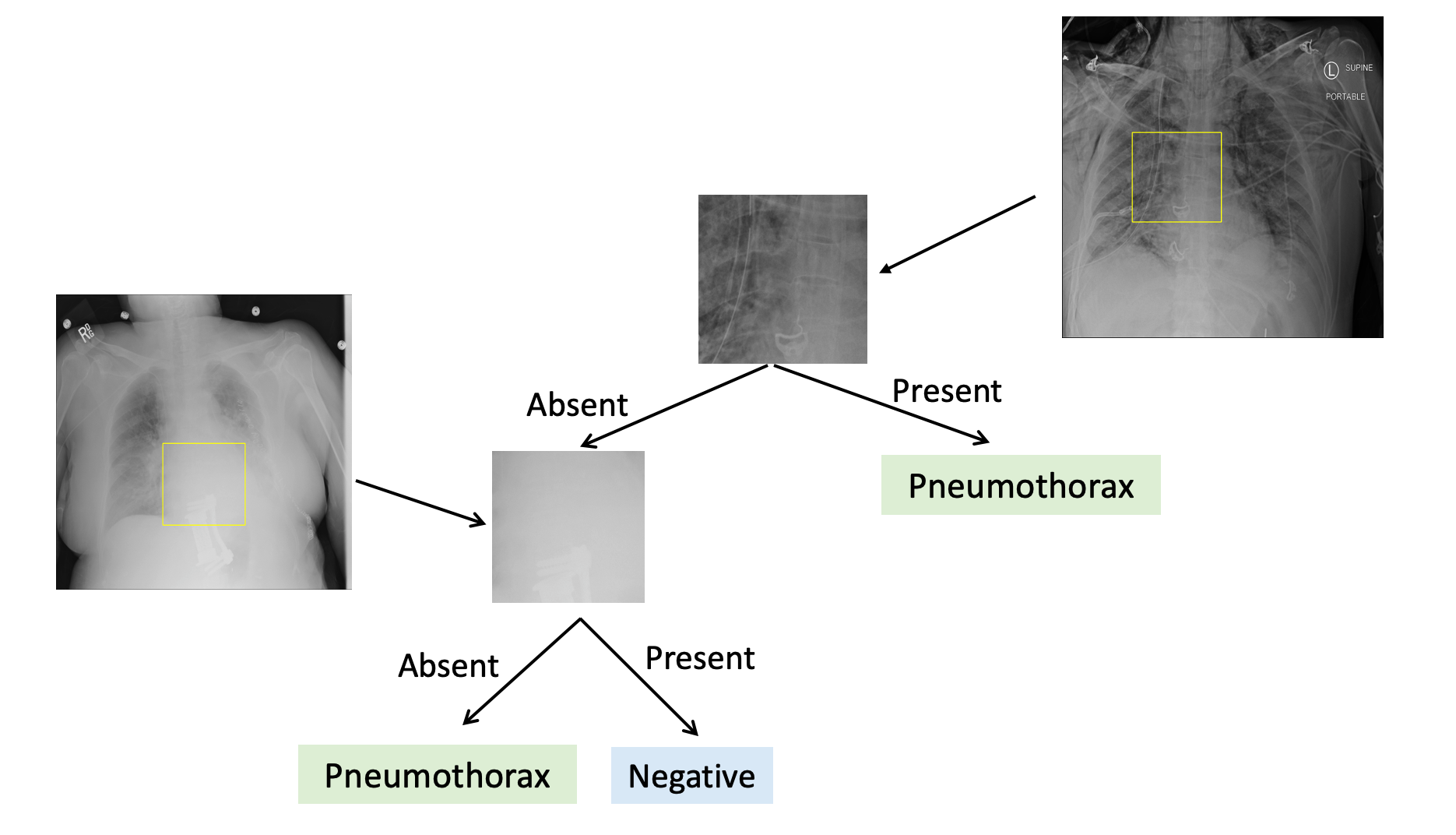


Figure 27. Global explanation of the NPT classifier’s decision-making process for detecting Pneumothorax (IC=3) with AP view CXRs in Chest X-ray 14.


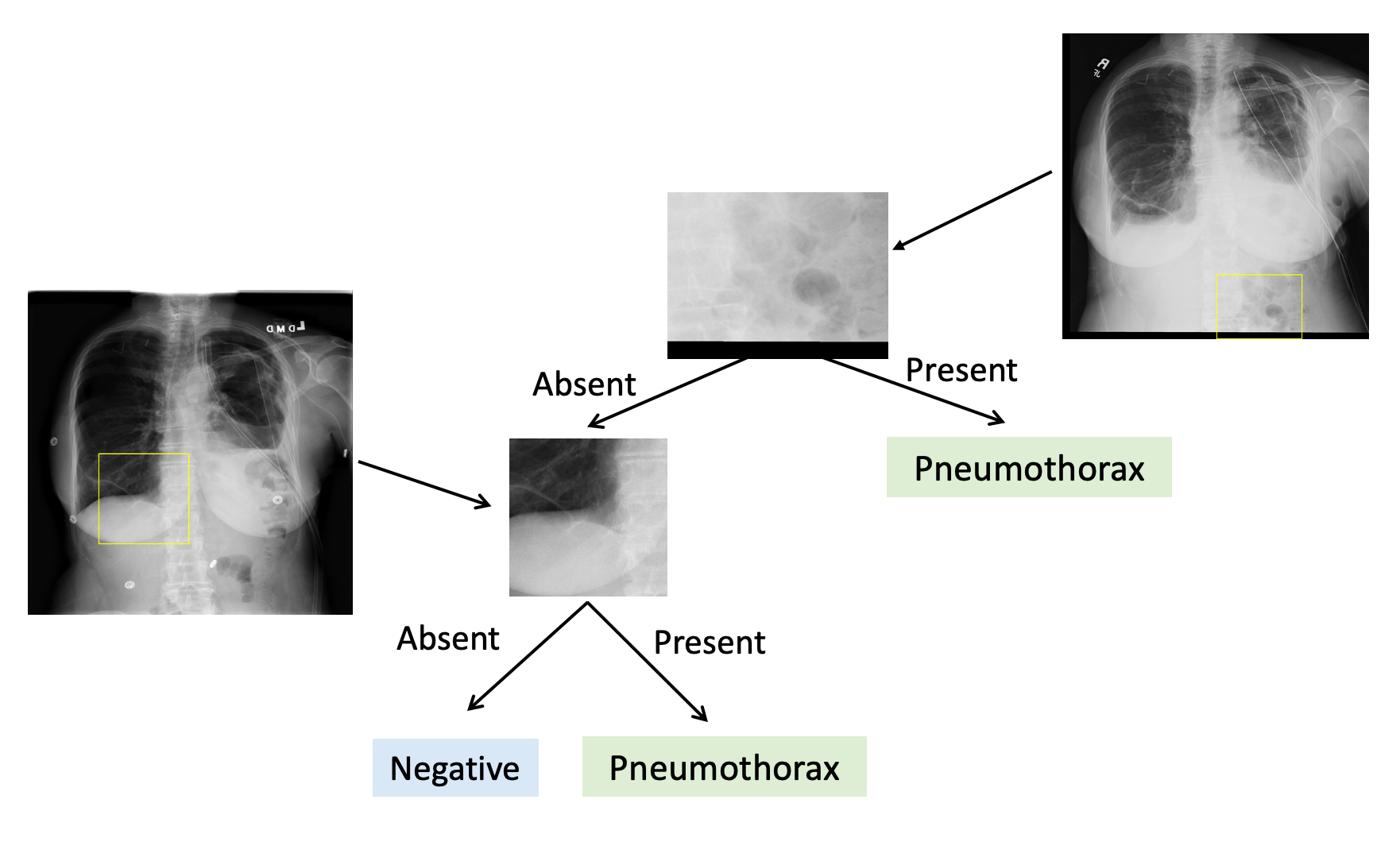


Figure 28. Global explanation of the NPT classifier’s decision-making process for detecting Pneumothorax (IC=3) with PA view CXRs in Chest X-ray 14.


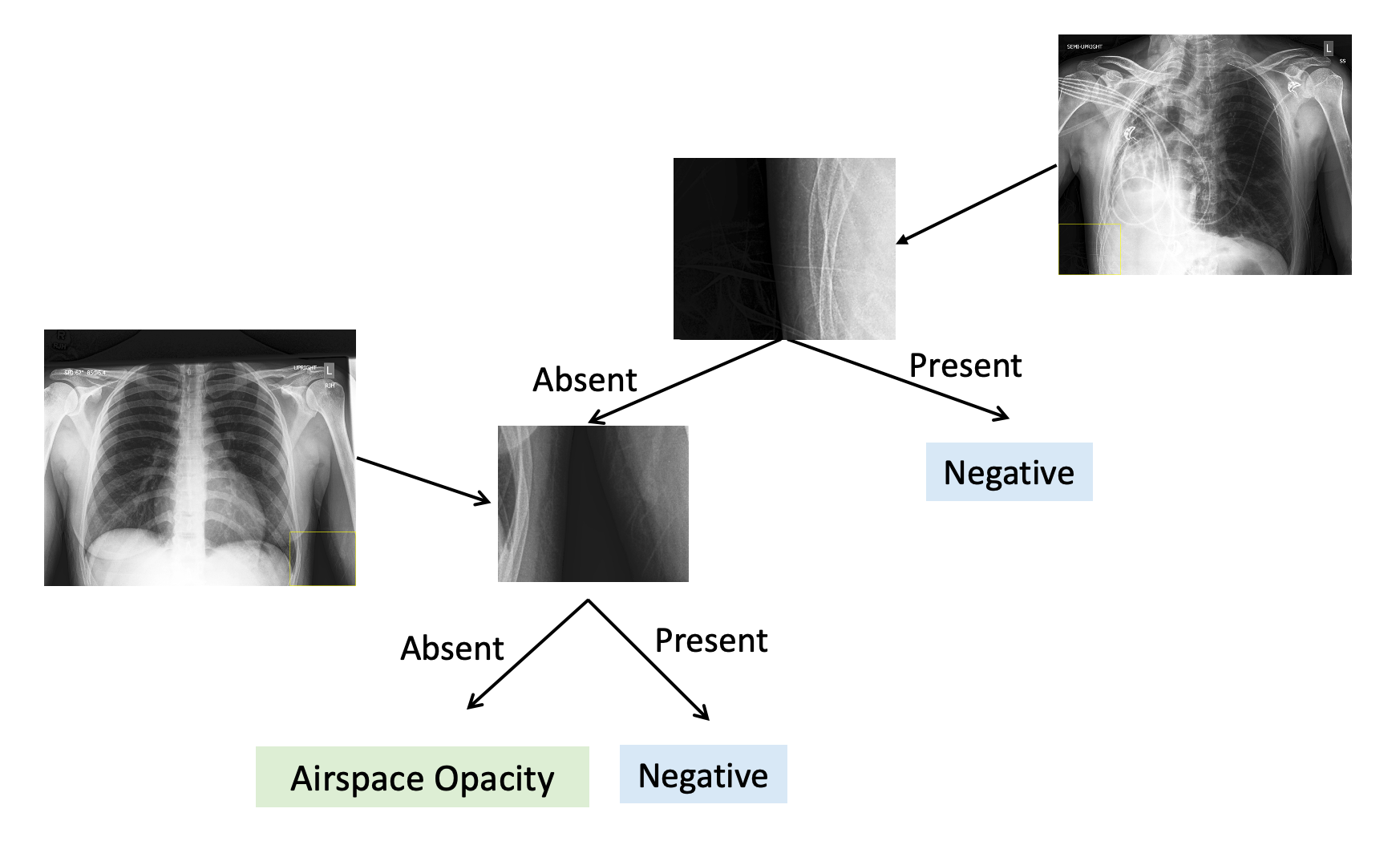


Figure 29. Global explanation of the NPT classifier’s decision-making process for detecting Airspace Opacity (IC=3) with AP view CXRs in CheXpert.


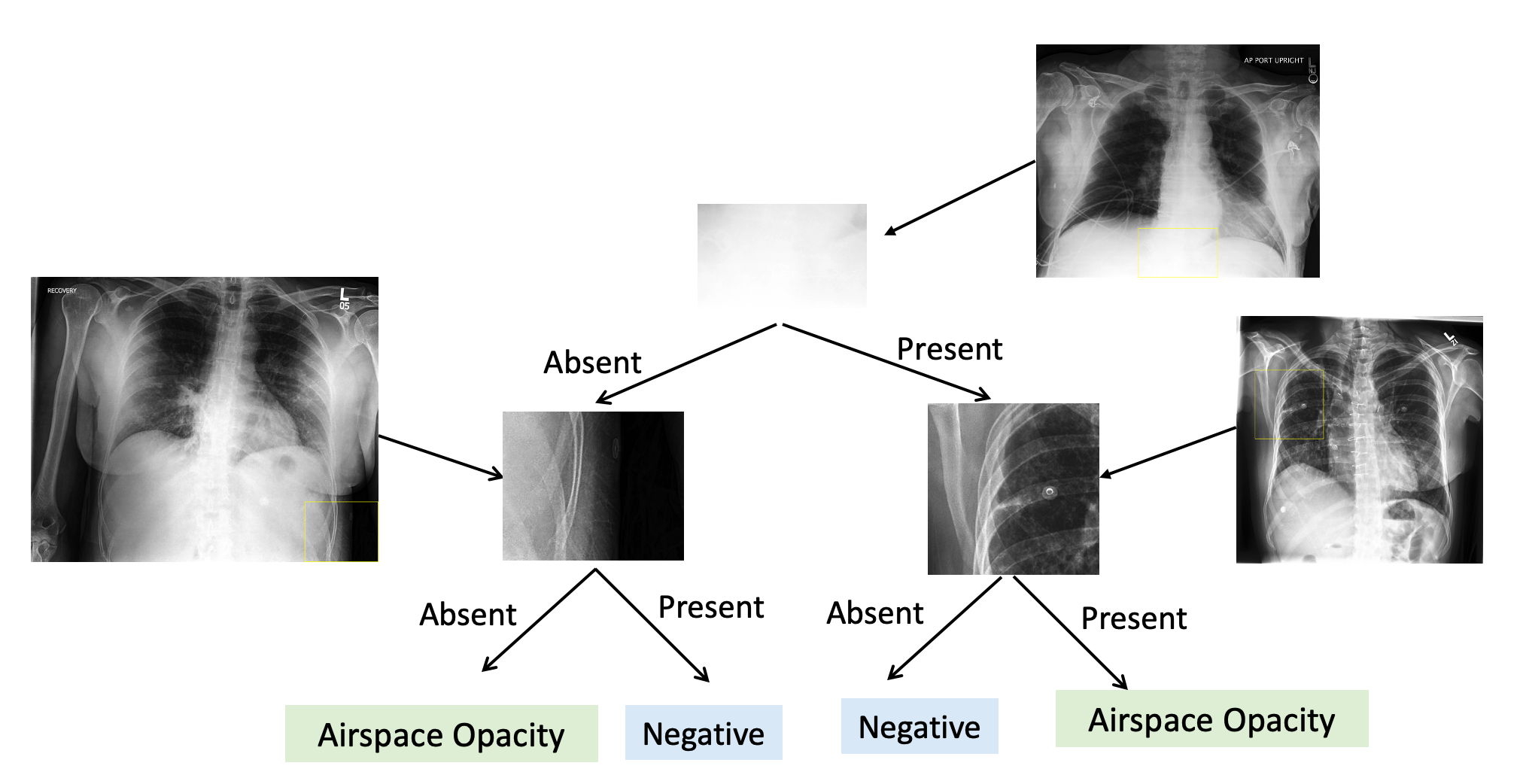


Figure 30. Global explanation of the NPT classifier’s decision-making process for detecting Airspace Opacity (IC=3) with PA view CXRs in CheXpert.


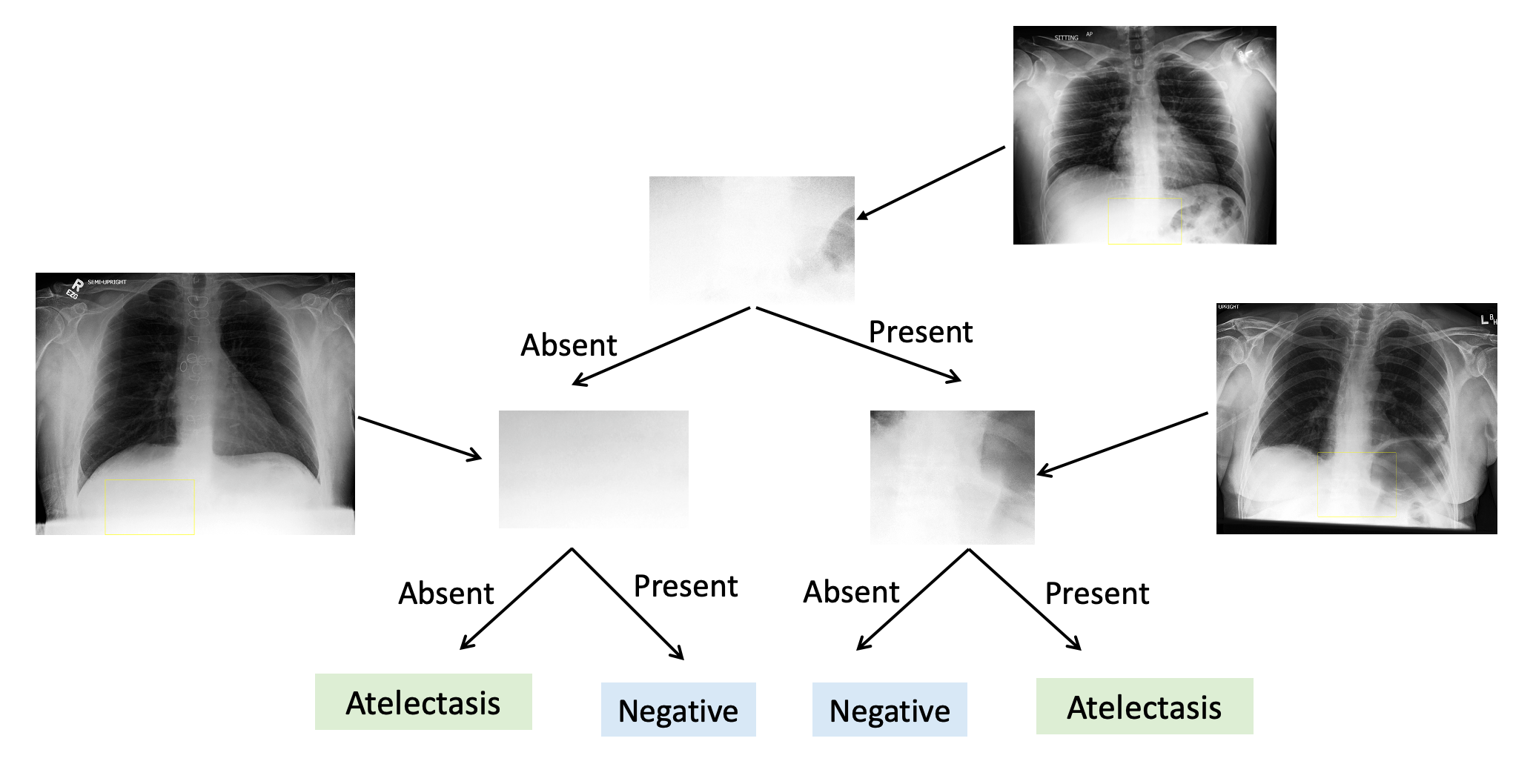


Figure 31. Global explanation of the NPT classifier’s decision-making process for detecting Atelectasis (IC=3) with AP view CXRs in CheXpert.


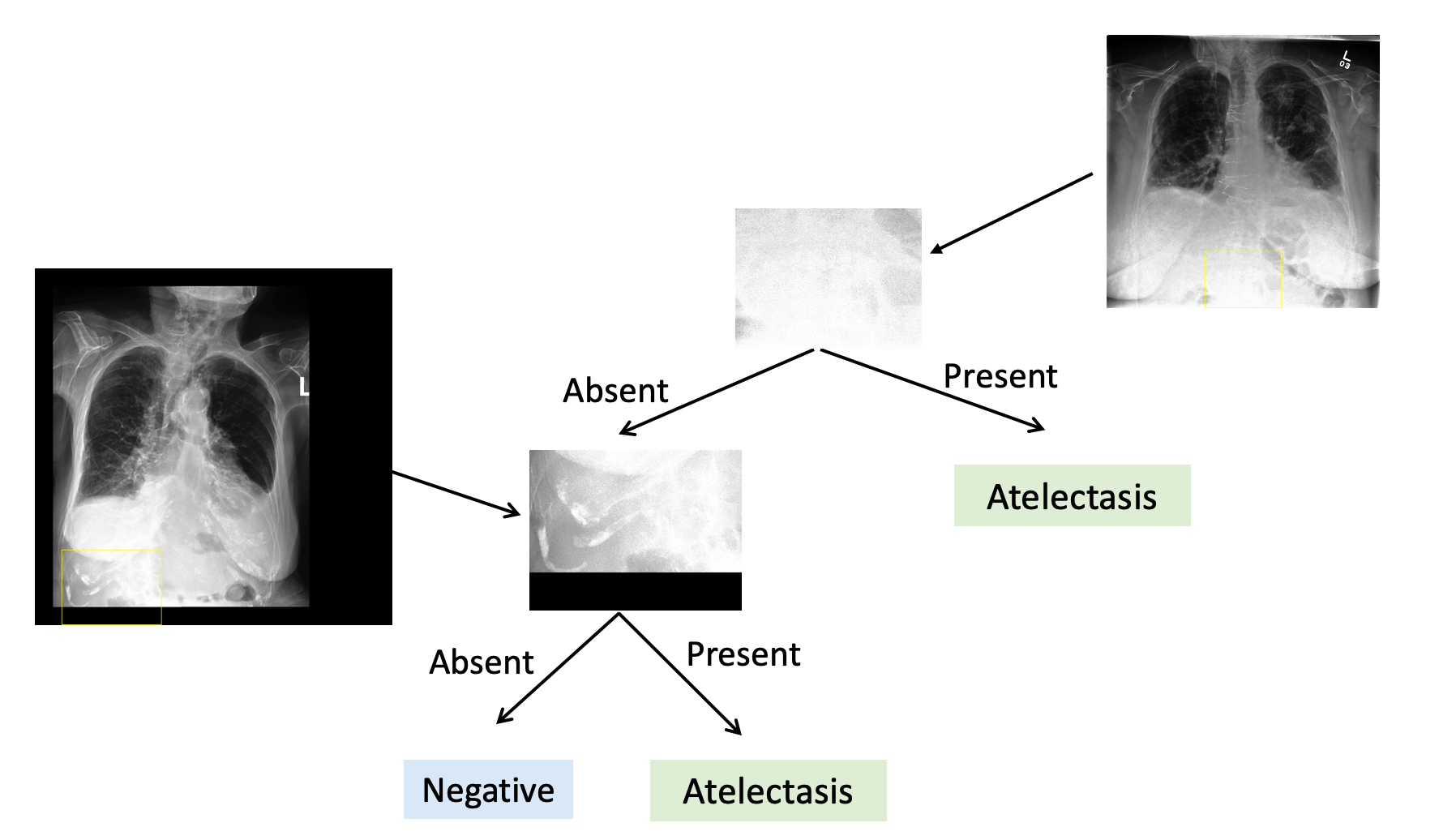


Figure 32. Global explanation of the NPT classifier’s decision-making process for detecting Atelectasis (IC=3) with PA view CXRs in CheXpert.


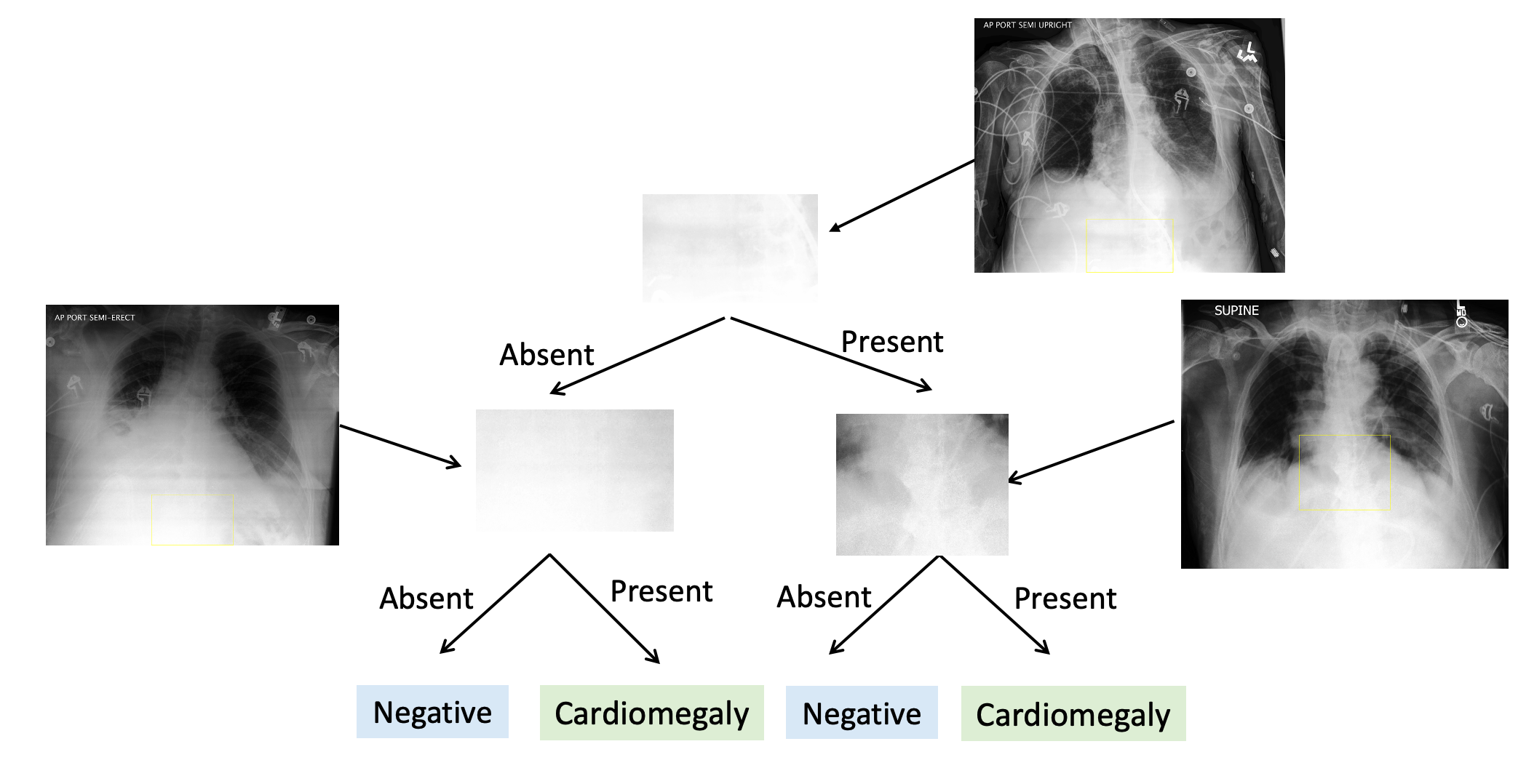


Figure 33. Global explanation of the NPT classifier’s decision-making process for detecting Cardiomegaly (IC=3) with AP view CXRs in CheXpert.


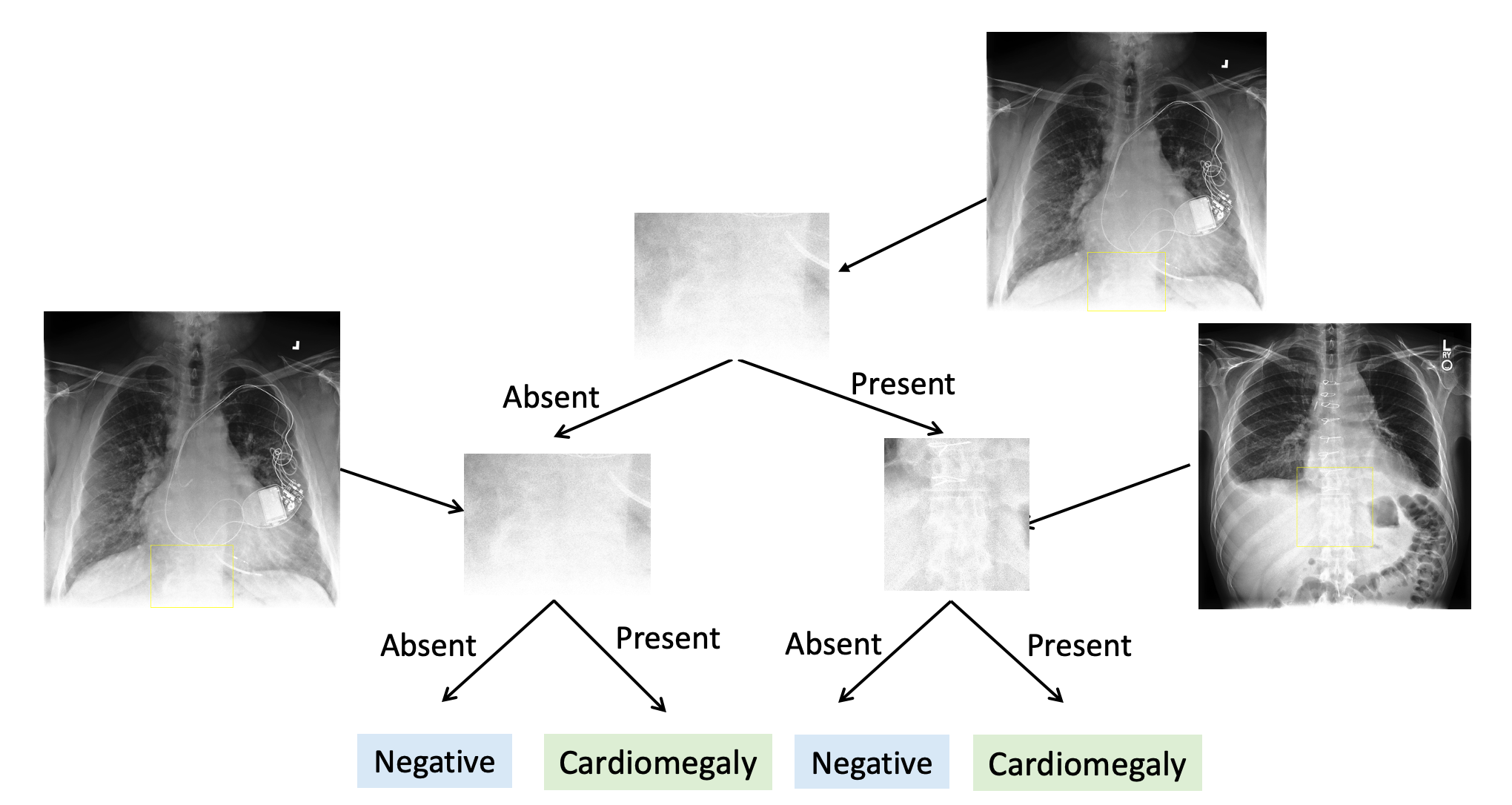


Figure 34. Global explanation of the NPT classifier’s decision-making process for detecting Cardiomegaly (IC=3) with PA view CXRs in CheXpert.


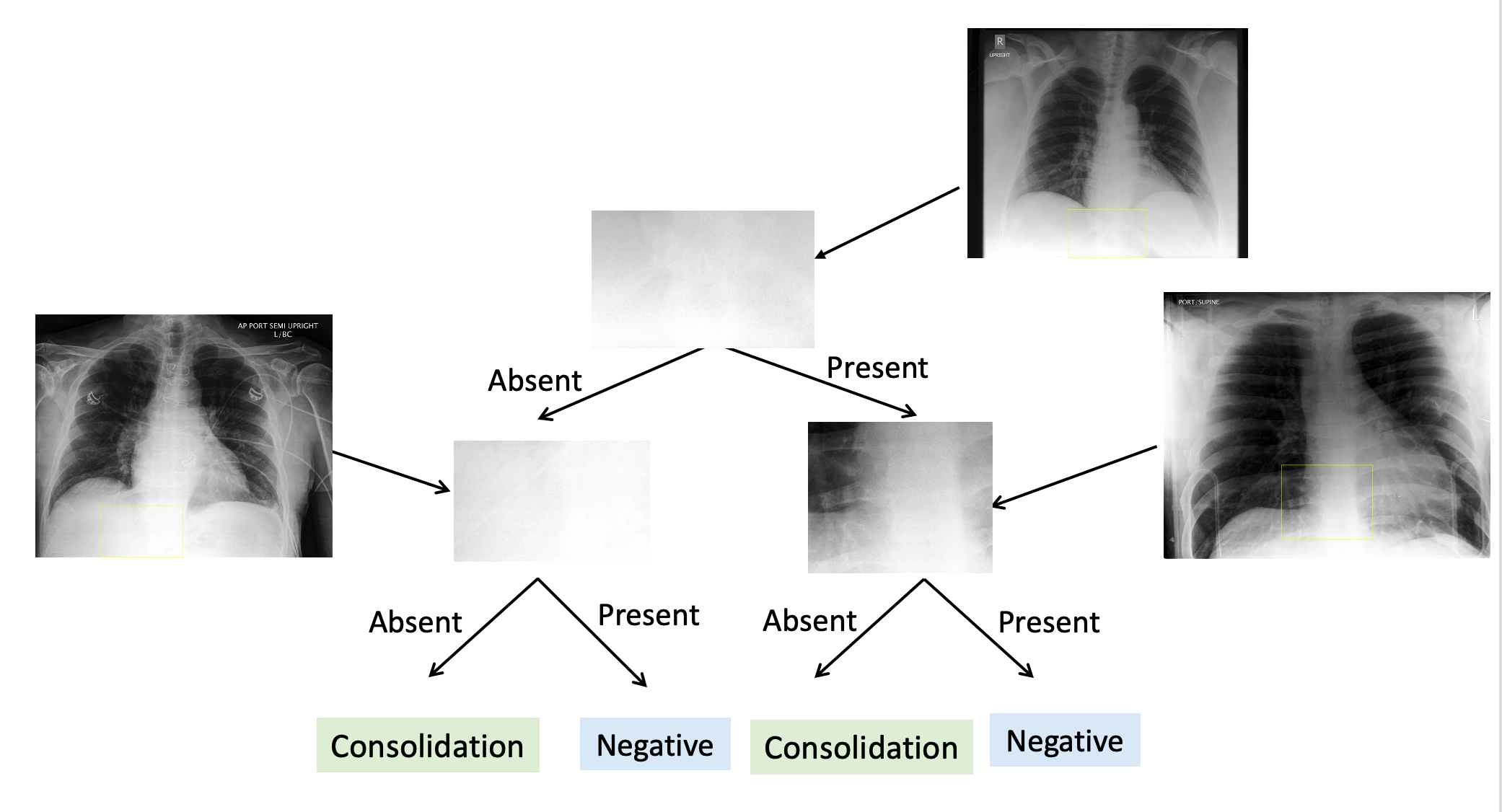


Figure 35. Global explanation of the NPT classifier’s decision-making process for detecting Consolidation (IC=3) with AP view CXRs in CheXpert.


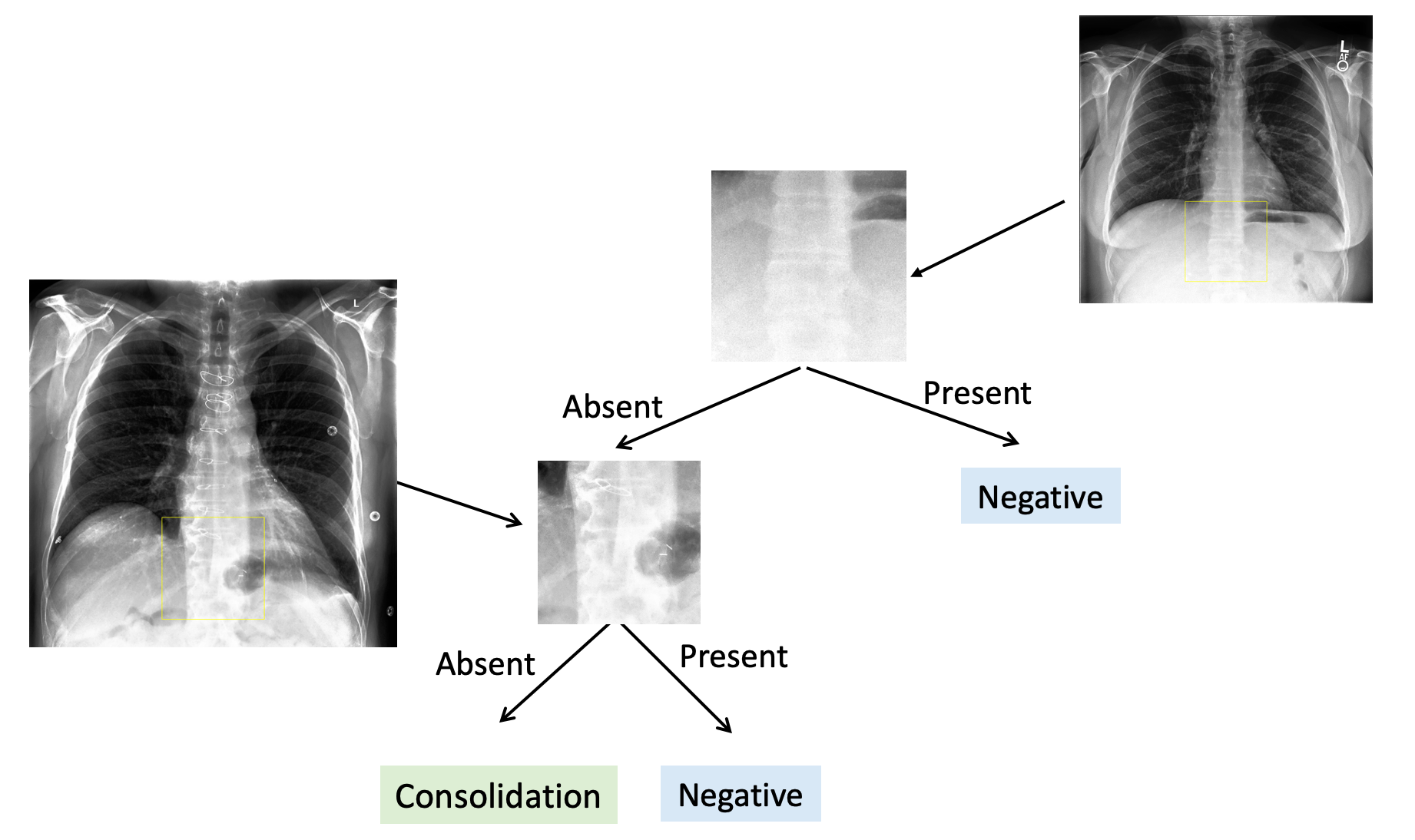


Figure 36. Global explanation of the NPT classifier’s decision-making process for detecting Consolidation (IC=3) with PA view CXRs in CheXpert.


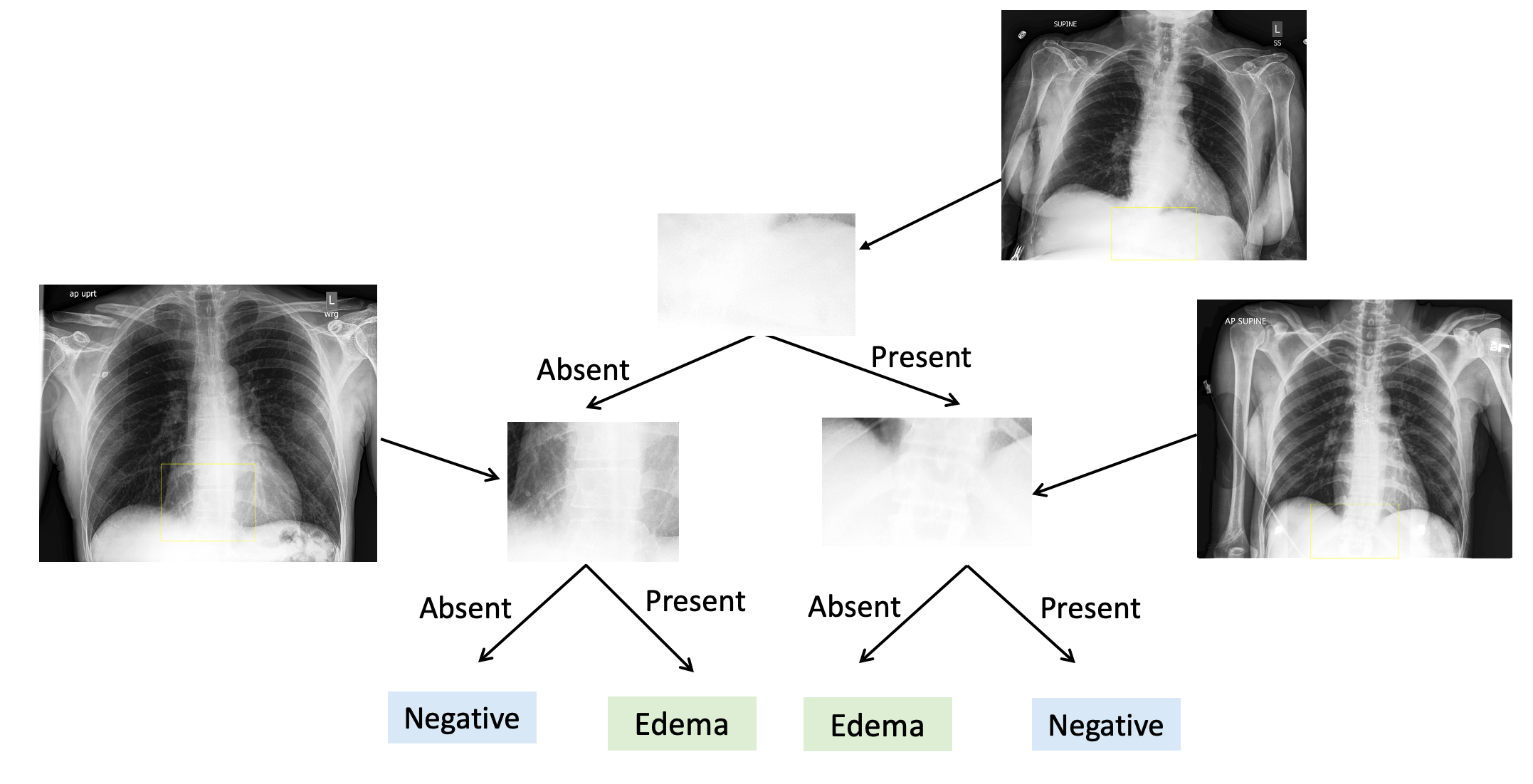


Figure 37. Global explanation of the NPT classifier’s decision-making process for detecting Edema (IC=3) with AP view CXRs in CheXpert.


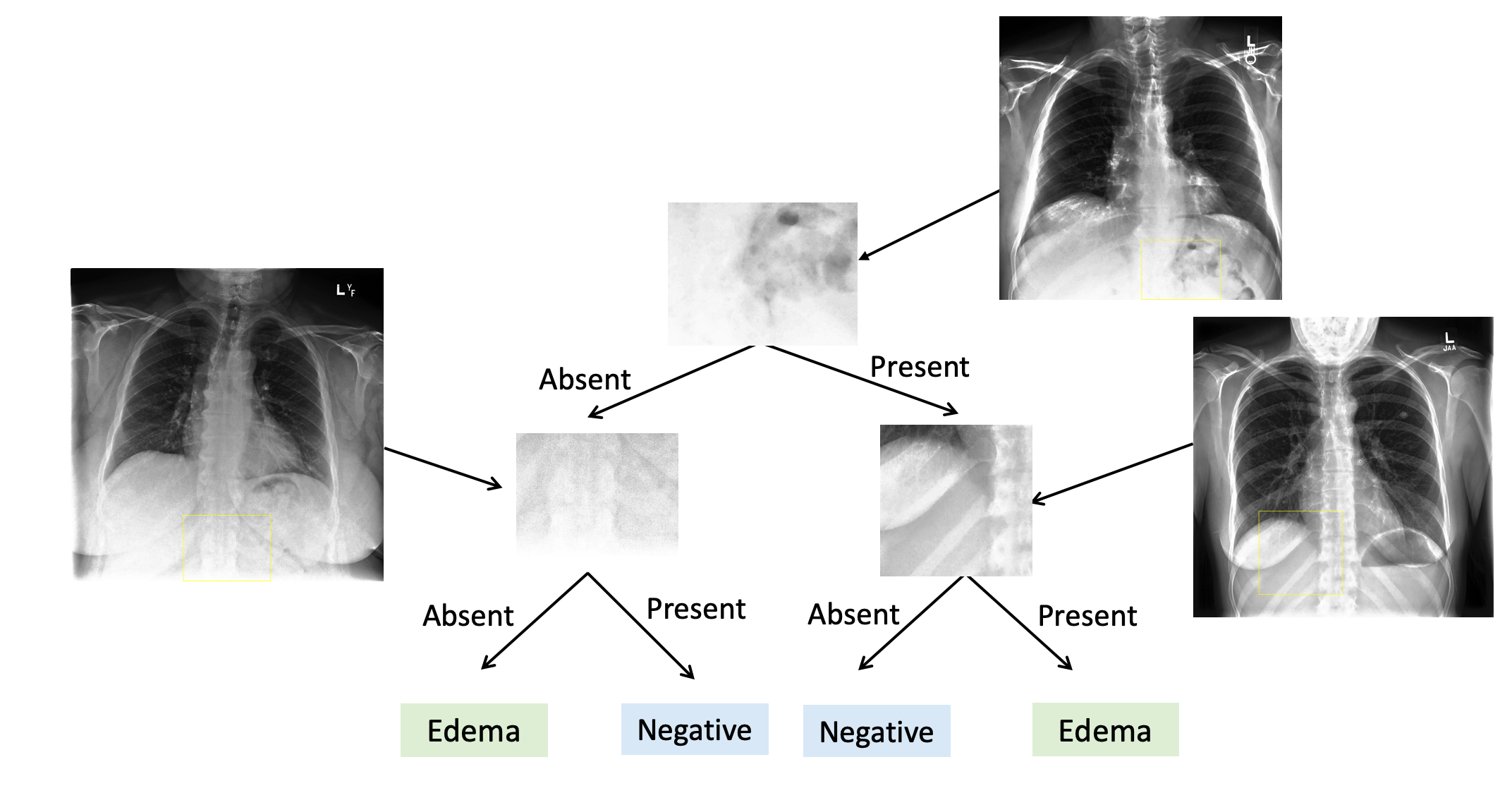


Figure 38. Global explanation of the NPT classifier’s decision-making process for detecting Edema (IC=3) with PA view CXRs in CheXpert.


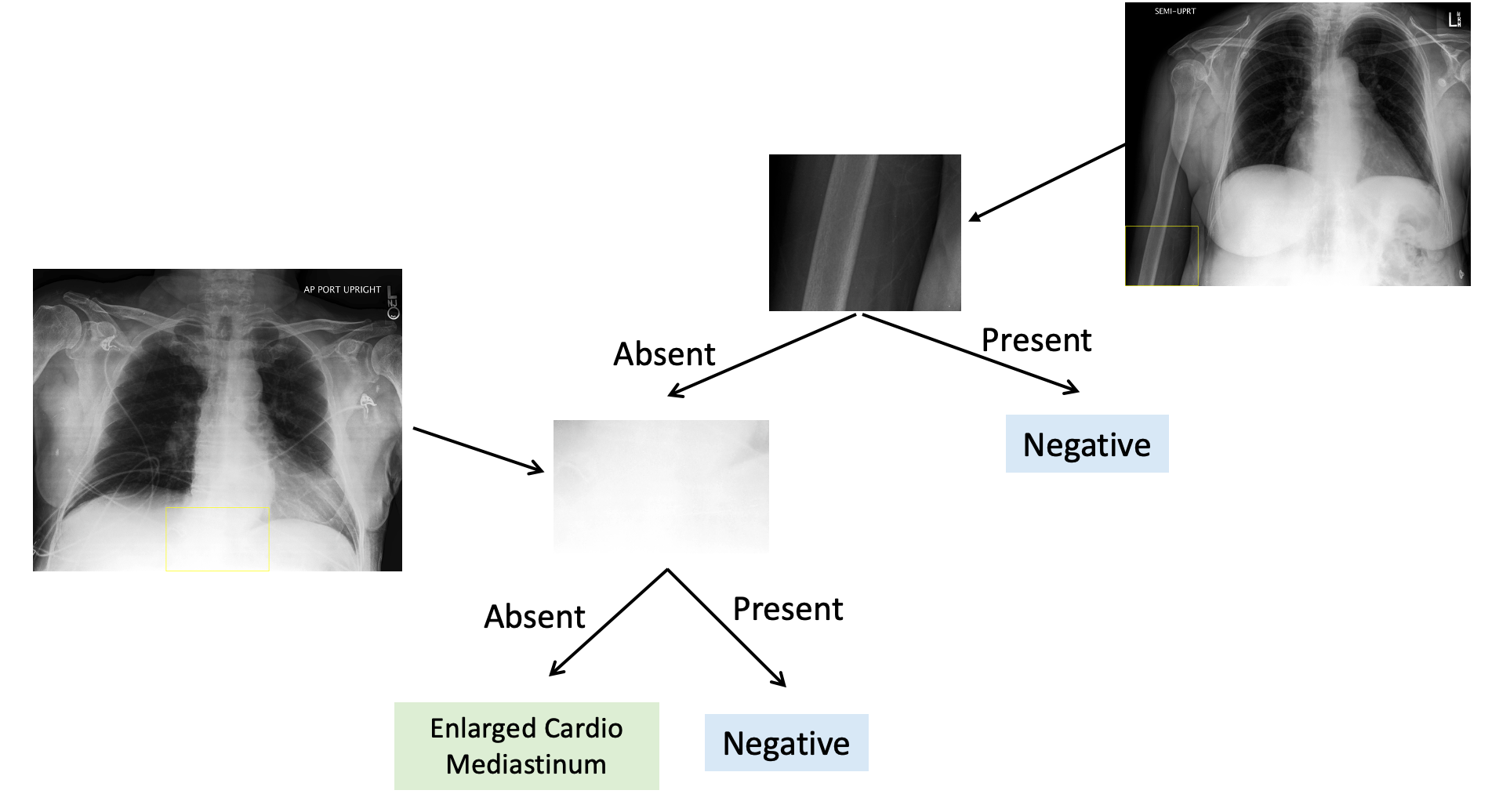


Figure 39. Global explanation of the NPT classifier’s decision-making process for detecting Enlarged Cardio Mediastinum (IC=3) with AP view CXRs in CheXpert.


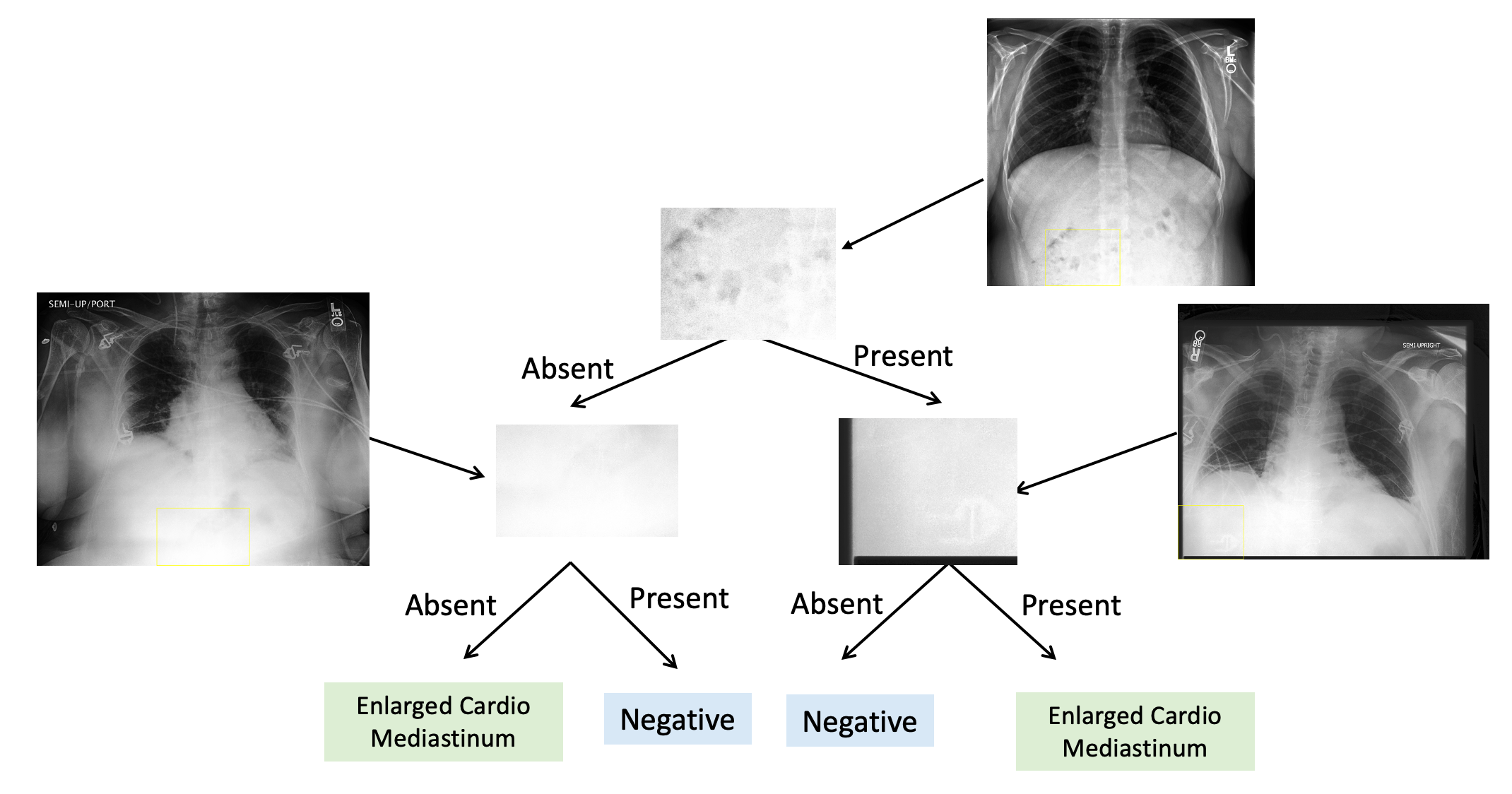


Figure 40. Global explanation of the NPT classifier’s decision-making process for detecting Enlarged Cardio Mediastinum (IC=3) with PA view CXRs in CheXpert.


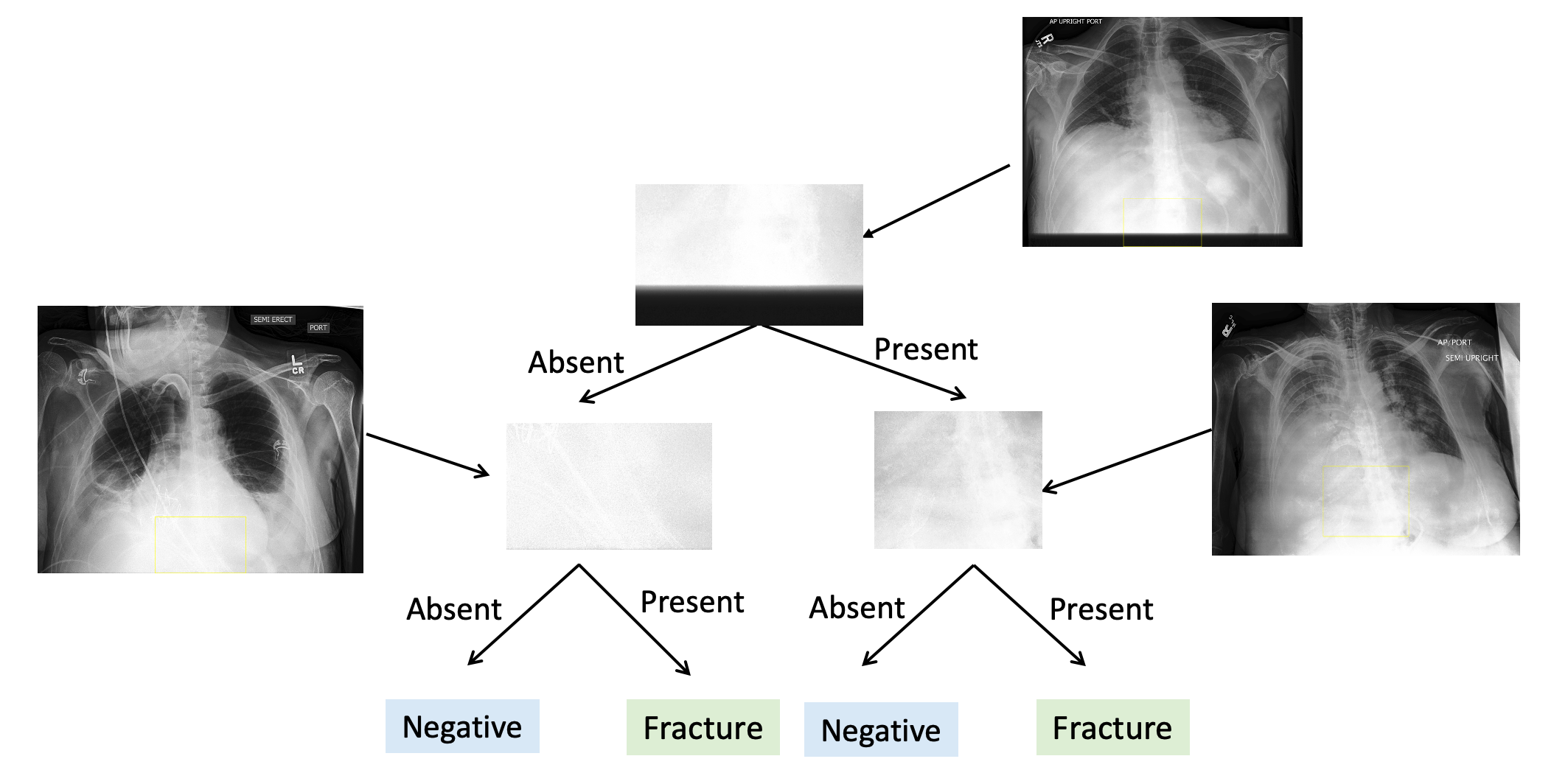


Figure 41. Global explanation of the NPT classifier’s decision-making process for detecting Fracture (IC=3) with AP view CXRs in CheXpert.


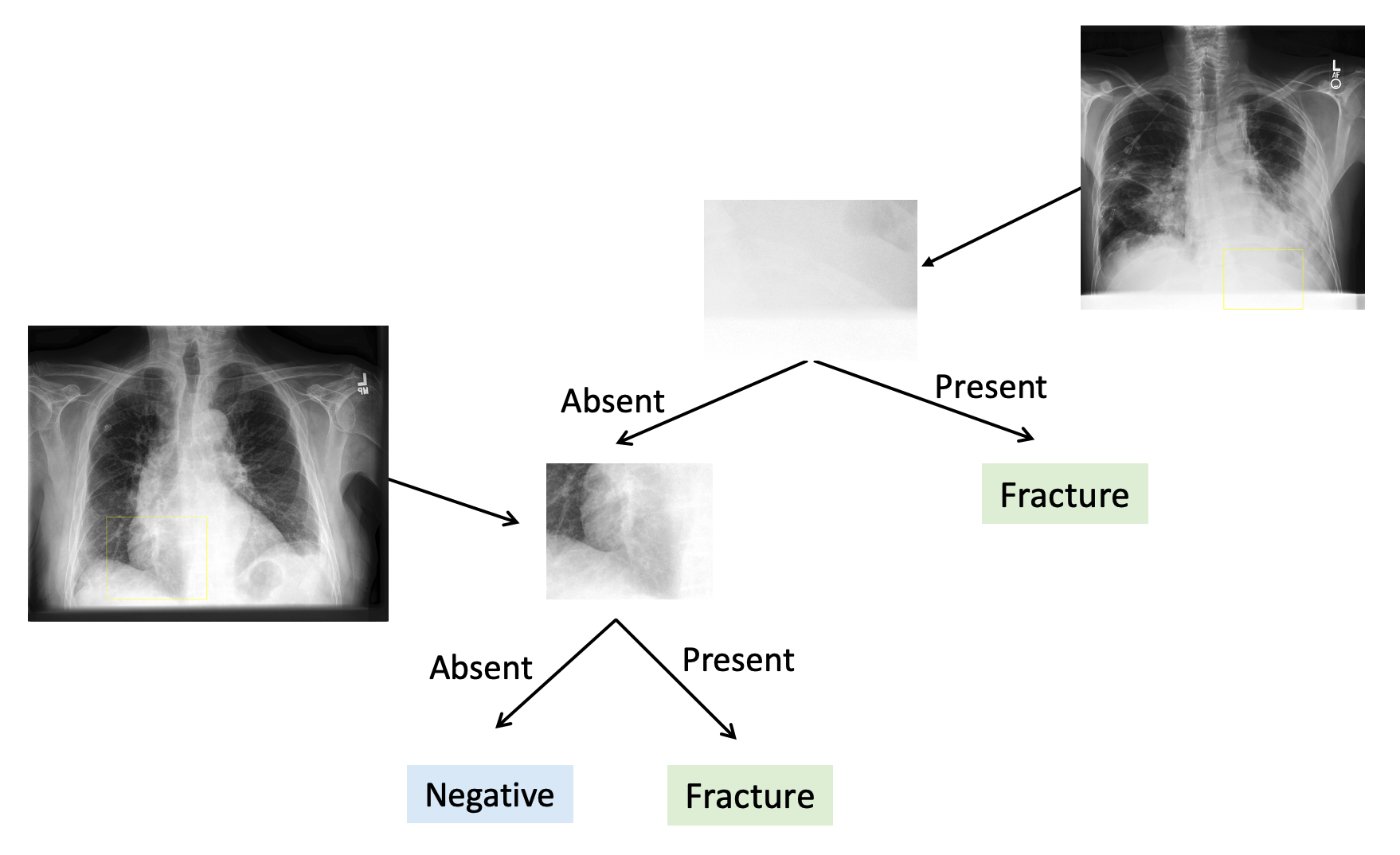


Figure 42. Global explanation of the NPT classifier’s decision-making process for detecting Fracture (IC=3) with PA view CXRs in CheXpert.


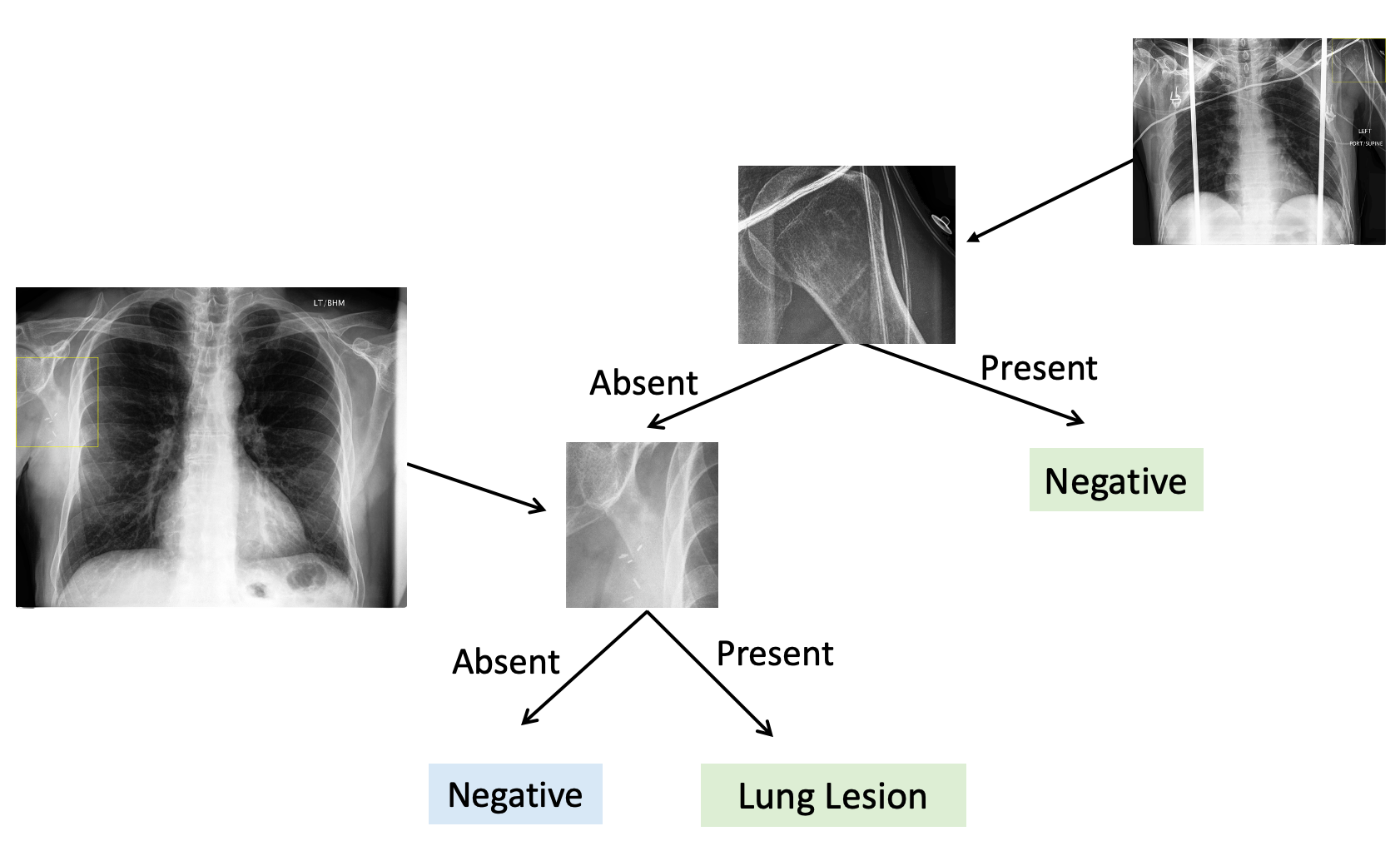


Figure 43. Global explanation of the NPT classifier’s decision-making process for detecting Lung Lesion (IC=3) with AP view CXRs in CheXpert.


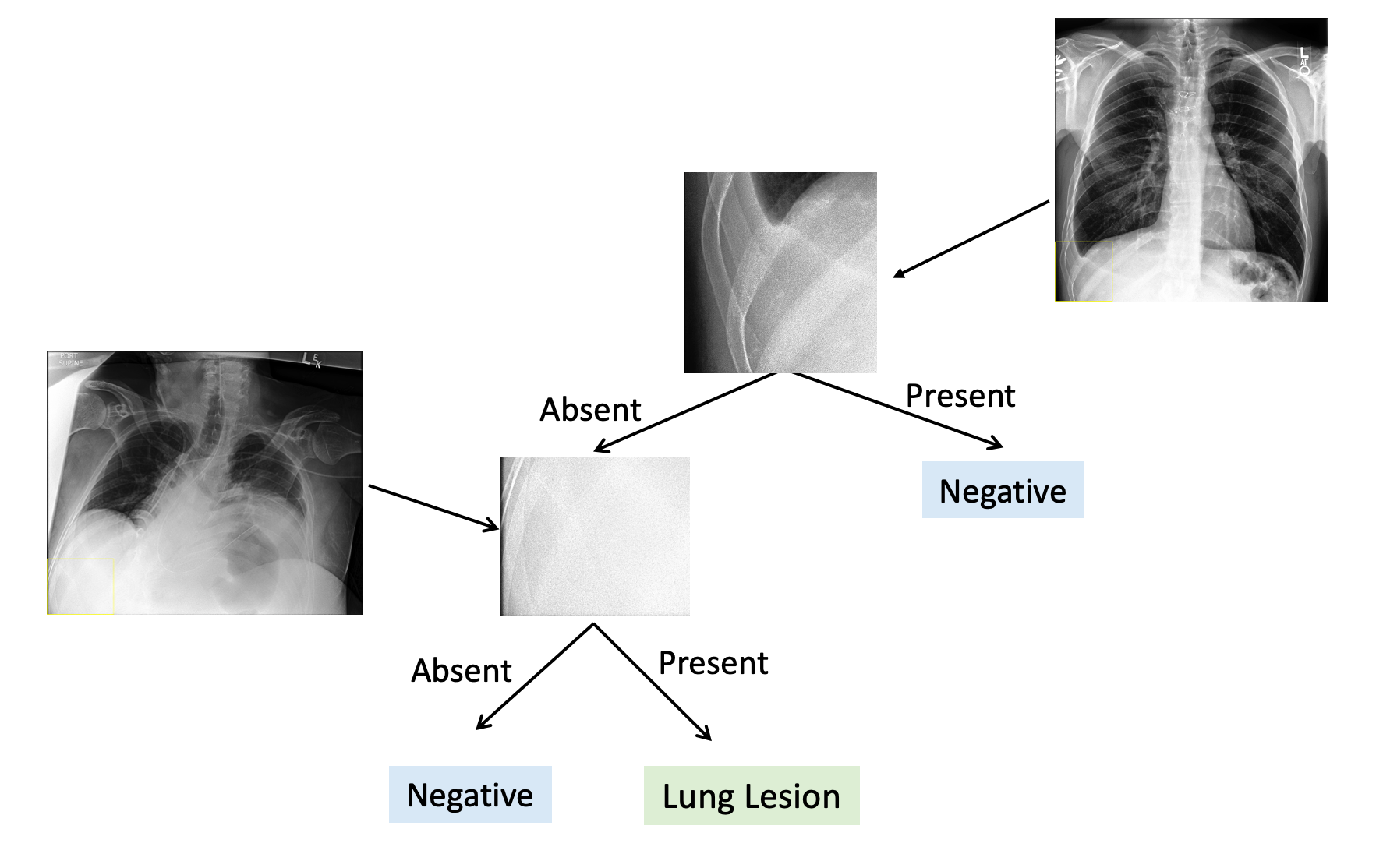


Figure 44. Global explanation of the NPT classifier’s decision-making process for detecting Lung Lesion (IC=3) with PA view CXRs in CheXpert.


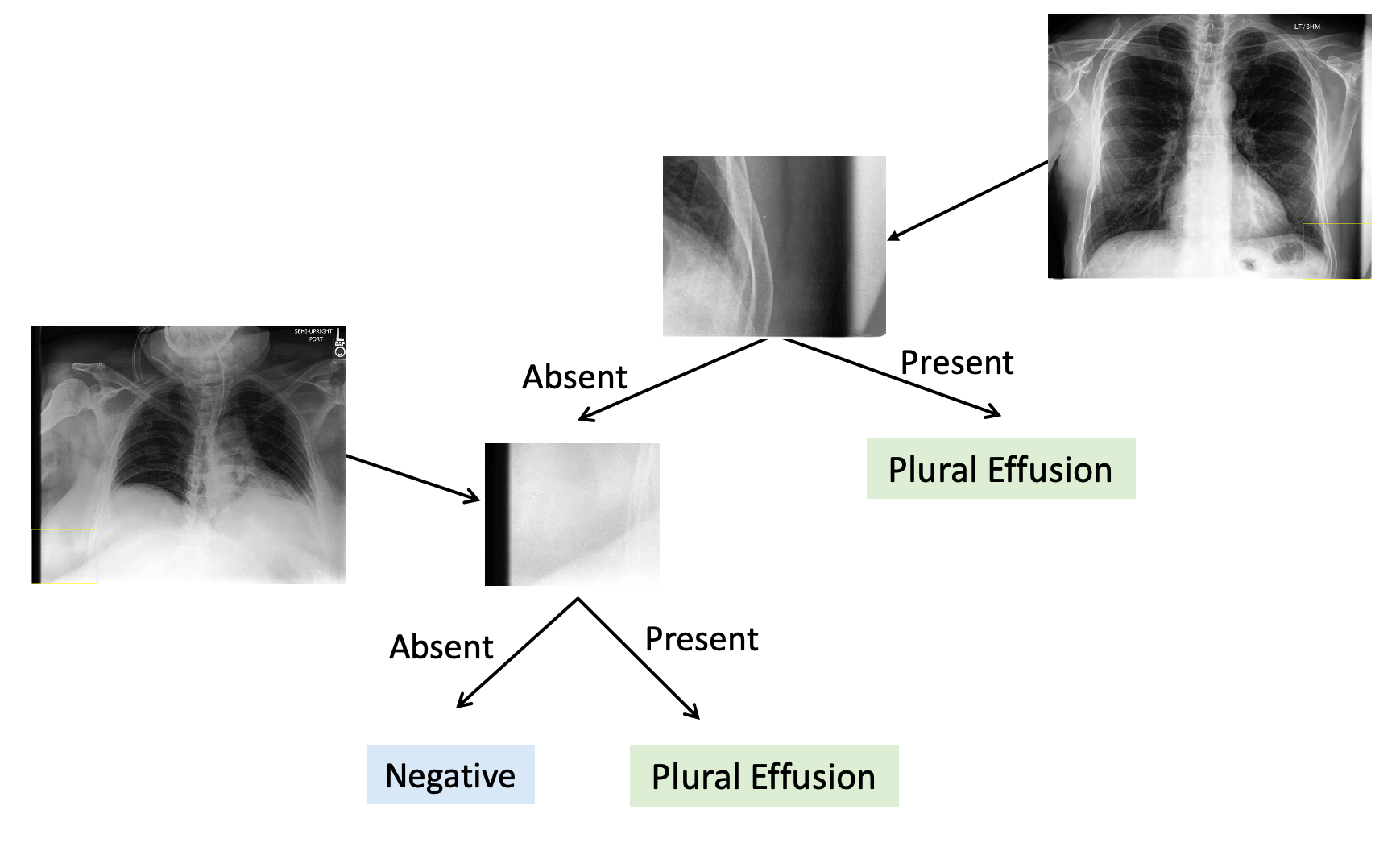


Figure 45. Global explanation of the NPT classifier’s decision-making process for detecting Plural Effusion (IC=3) with AP view CXRs in CheXpert.


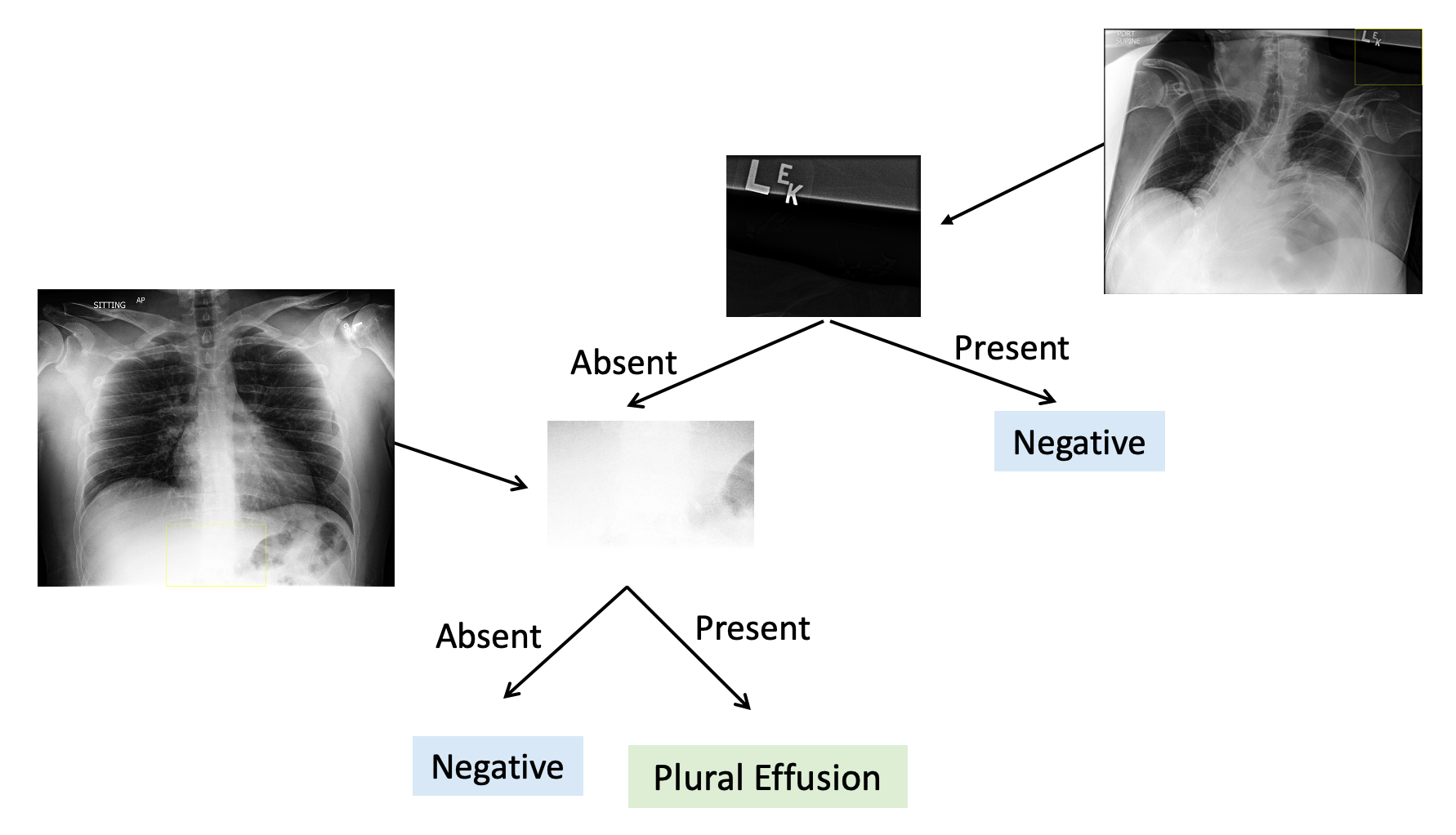


Figure 46. Global explanation of the NPT classifier’s decision-making process for detecting Plural Effusion (IC=3) with PA view CXRs in CheXpert.


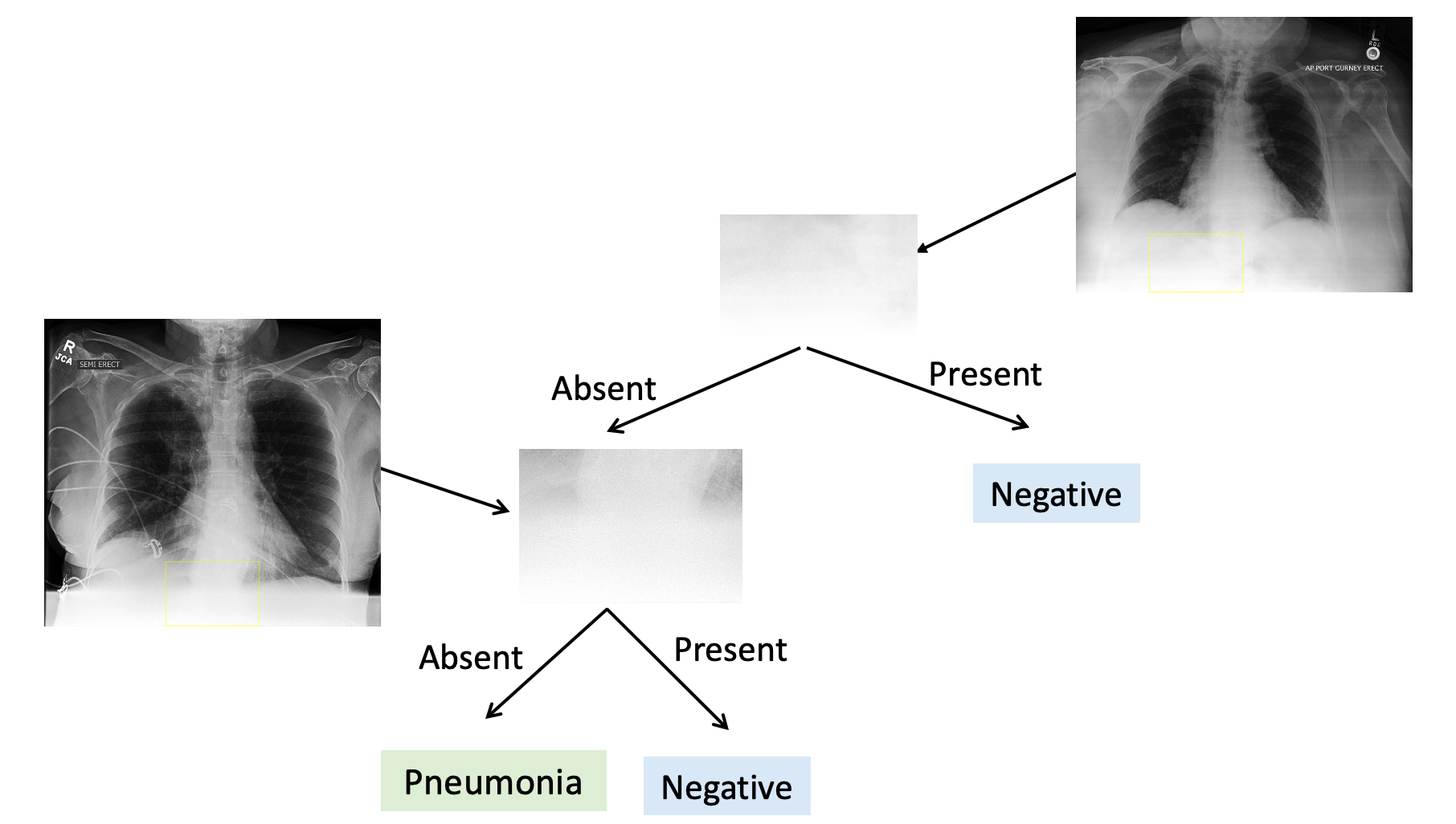


Figure 47. Global explanation of the NPT classifier’s decision-making process for detecting Pneumonia (IC=3) with AP view CXRs in CheXpert.


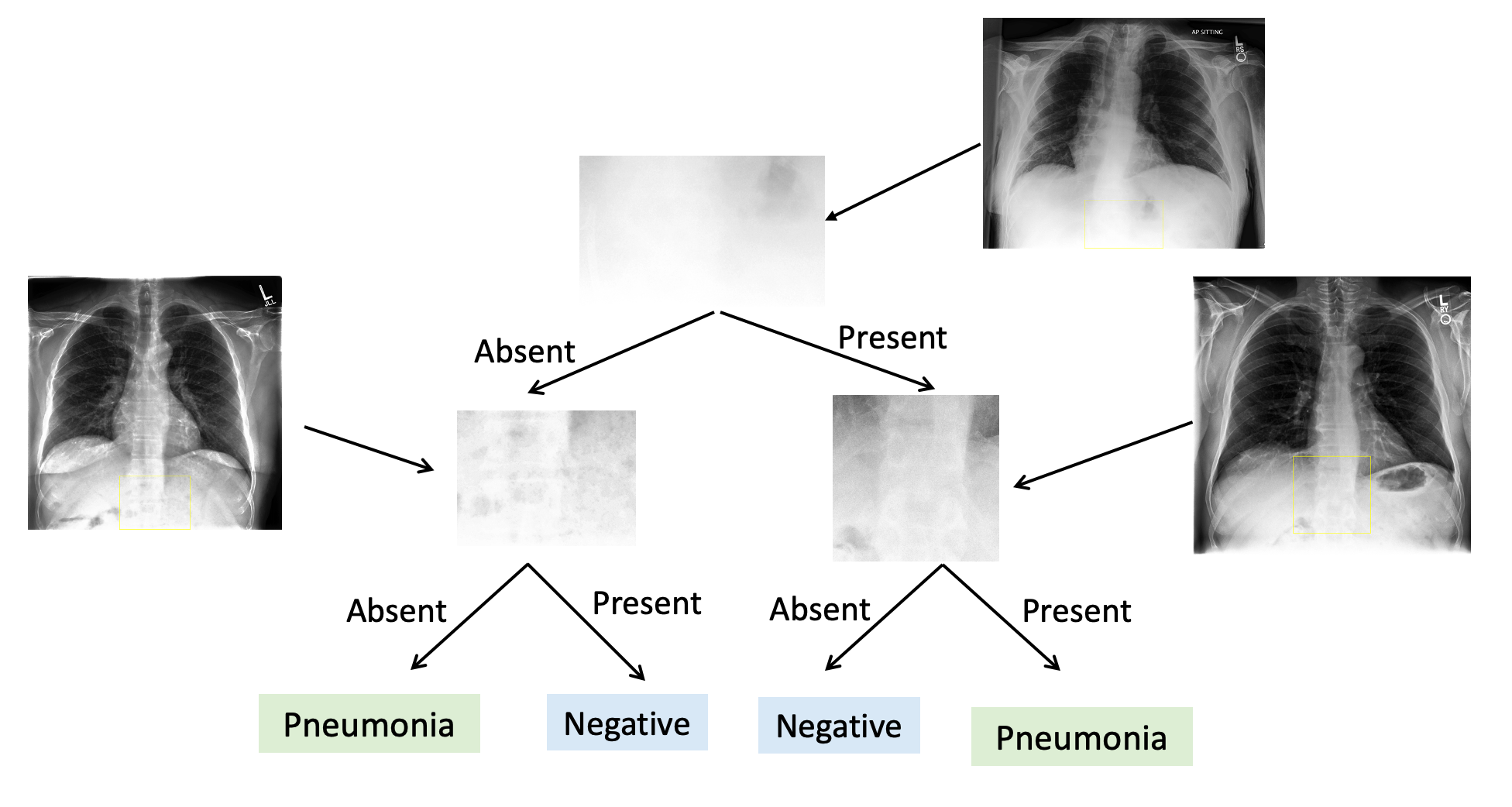


Figure 48. Global explanation of the NPT classifier’s decision-making process for detecting Pneumonia (IC=3) with PA view CXRs in CheXpert.


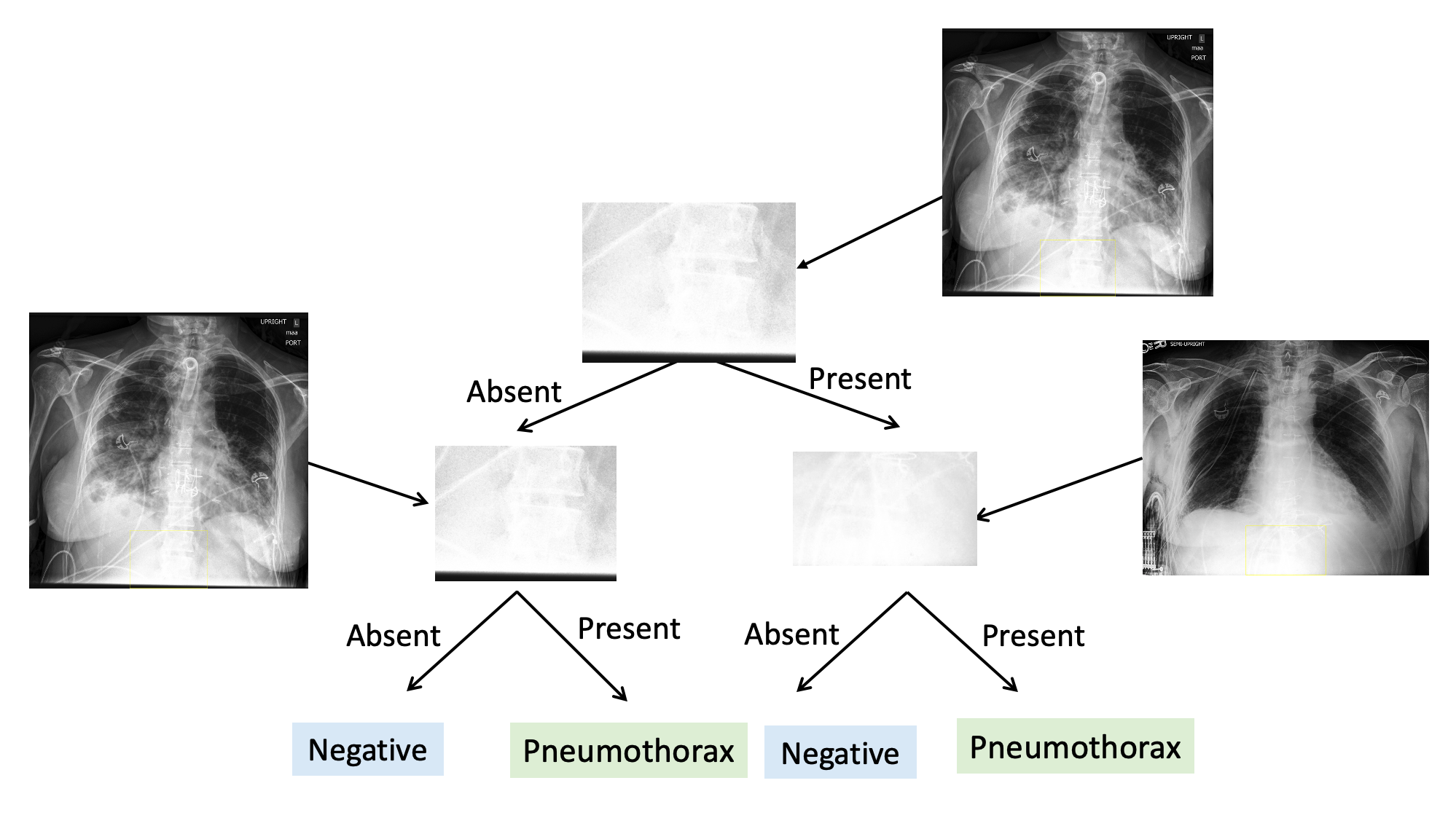


Figure 49. Global explanation of the NPT classifier’s decision-making process for detecting Pneumothorax (IC=3) with AP view CXRs in CheXpert.


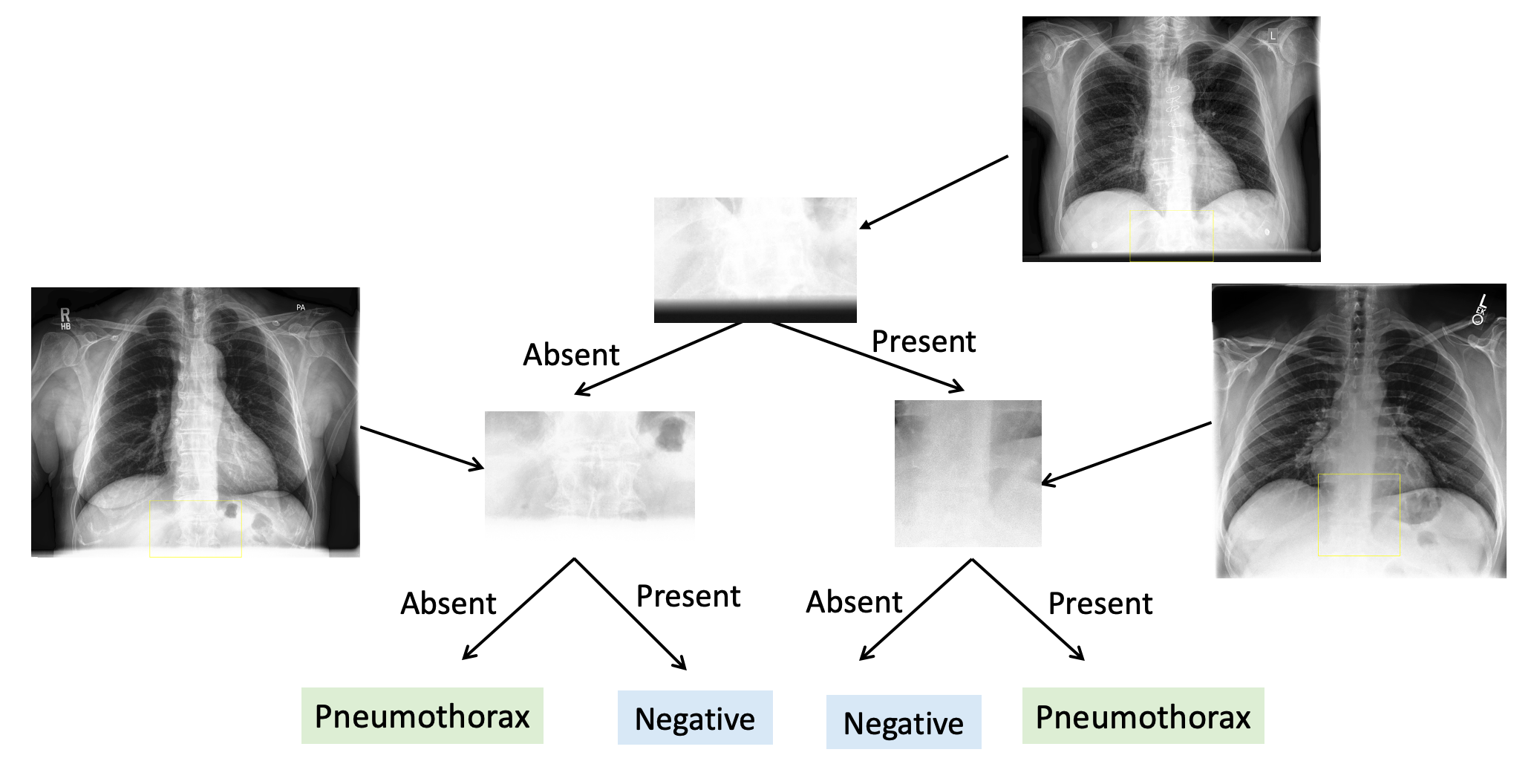


Figure 50. Global explanation of the NPT classifier’s decision-making process for detecting Pneumothorax (IC=3) with PA view CXRs in CheXpert.
